# Model based planners reflect on their model-free propensities

**DOI:** 10.1371/journal.pcbi.1008552

**Published:** 2021-01-07

**Authors:** Rani Moran, Mehdi Keramati, Raymond J. Dolan

**Affiliations:** 1 Max Planck UCL Centre for Computational Psychiatry and Ageing Research, University College London, London, United Kingdom; 2 Wellcome Centre for Human Neuroimaging, University College London, London, United Kingdom; 3 Department of Psychology, City, University of London, London, United Kingdom; Dartmouth College, UNITED STATES

## Abstract

Dual-reinforcement learning theory proposes behaviour is under the tutelage of a retrospective, value-caching, model-free (MF) system and a prospective-planning, model-based (MB), system. This architecture raises a question as to the degree to which, when devising a plan, a MB controller takes account of influences from its MF counterpart. We present evidence that such a sophisticated self-reflective MB planner incorporates an anticipation of the influences its own MF-proclivities exerts on the execution of its planned future actions. Using a novel bandit task, wherein subjects were periodically allowed to design their environment, we show that reward-assignments were constructed in a manner consistent with a MB system taking account of its MF propensities. Thus, in the task participants assigned higher rewards to bandits that were momentarily associated with stronger MF tendencies. Our findings have implications for a range of decision making domains that includes drug abuse, pre-commitment, and the tension between short and long-term decision horizons in economics.

## Author summary

Extant theories of model-based (MB) planning usually ignore whether one’s model-free (MF) tendencies cohere, or conflict, with one’s desired goal-directed actions. From the perspective of these theories, one’s MF disposition to abstinence or indulgent alcohol-drinking is irrelevant for a planner who intends to avoid alcohol consumption at a forthcoming party. However, MF tendencies can hijack one’s actions such that plans consistent with MF tendencies are often adhered to, whereas plans that conflict with MF tendencies are prone to be disregarded. Here, we show that planners consider whether their MF proclivities facilitate or hinder goal-pursuit. We show that people are disposed to design a task-environment in such a way that renders MB goals consistent (rather than conflicting) with their MF dispositions.

## Introduction

The idea that human action is controlled by multiple systems is prominent in theories of “dual-control” Reinforcement Learning (RL) [1–17], and is widespread in fields as disparate as Psychology, Neuroscience, Economics, and Artificial Intelligence. Within an RL framework behavioural control is implemented either via a rigid, retrospective and fast model-free (MF) system [18,19] or via a flexible, goal-directed, prospective, computationally demanding model-based (MB) system [18,20]. Unlike a putative MF system, which tends to repeat past successful actions, a MB system deliberates on the prospective efficiency of potential actions. While much extant research on “Multiple-control” has focused on an arbitration between these two systems, the extent to which different controllers take account of each other’s influences has largely been ignored. Here, we pose the fundamental question as to whether a MB planner considers a possibility that its future action choices might be subject to MF influences. We provide evidence that this is indeed the case.

RL theories of MB planning tend to disregard a consideration of MF dispositions. Indeed, existing treatments take it as given that future actions will be controlled solely by a MB system. For example, a Bellman planner assumes that future actions will be those that maximise MB value calculations (disregarding the MF values of these actions) i.e., they will be optimal [18,21]. Importantly, evidence that individuals rely on a mixture of MF and MB strategies [7,20] casts doubt on the utility of a ‘pure MB-horizon’ i.e., a MF-ignorant, planner, which lacks a reliable model of a MF disposed self.

**Fig 1:**
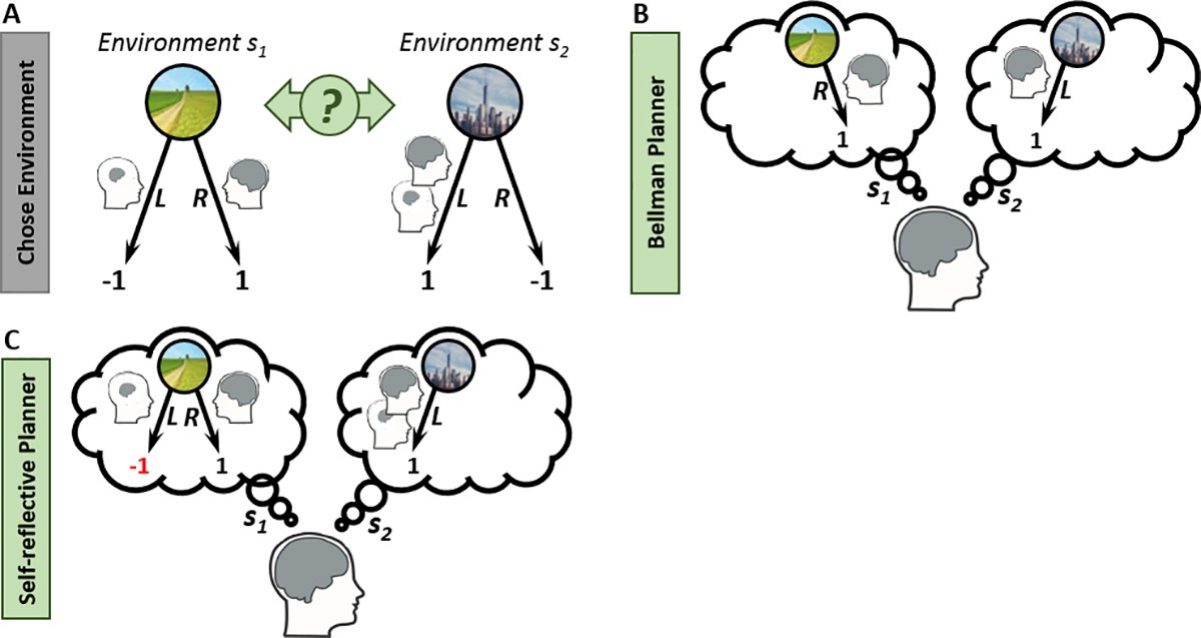
Illustrations of differences between a Bellman and a Self-Reflective MB Planner. A) Environmental Design. Consider two different environments denoted s1 and s2, each affording a ‘left’ (L) and a ‘right’ (R) action. Momentarily, the MB system state-action values are as depicted by the black numbers (the preferred MB action is depicted as a big brain). Additionally, assume that momentarily the MF system has a tendency to choose L in both environments (depicted by a small brain). Assume that the agent is asked to choose an environment for its next action. (B) A Bellman, MF-ignorant, MB-planner evaluates a choice of environment s1. From the planner’s perspective V_Bellman_(s1) = 1 because the planner assumes that in s1, the agent will choose R. Similarly, V_Bellman_(s2) = 1 (as in s2 action L will be taken). Hence, a Bellman planner is indifferent between environment s1 to s2. C) A self-reflective MB planner, who anticipates the possibility of succumbing to a MF-inclination, will prefer environment s2 over s1, because she will realize that in s1, MF-influences bears the risk that action L (which the planner believes is penalizing) will be taken. In s2, on the other hand, the MB-desirable action R will be taken even if MF-proclivities influence the action.

We propose a novel theory of a *self-reflective MB planning*. Here, the central idea is that a MB system maintains a representation of its MF dispositions and, as a consequence, takes account of these when planning future actions by anticipating how prospective world-states will trigger currently-latent MF tendencies. We focus on a common situation wherein a goal-directed RL agent can choose or design an environment within which it will later seek rewards (e.g., which of several job offers to accept). Our central proposal is that a consideration of one’s own MF tendencies will impact the design of a future reward environment. To illustrate, consider an agent who acts in two environments (s1 and s2), each affording a ‘Left’ (L) and a ‘Right’ (R) action (Fig 1A), where momentarily, the MB system assigns a value of 1 to action R in s1 and L in s2 and a value of -1 to the two other state-action pairs. Additionally, the momentary MF tendencies are to choose L is both environments. Assume that the agent is asked to choose an environment for its next action. Importantly, if the agent chooses s2 she/he will then act in an environment where the desired MB action (L) is congruent with a concurrent MF-propensity. On the other hand, if the agent chooses environment s1, she/he will then act in an environment where the desired MB action (R) conflicts with a MF proclivity (L). A Bellman, MF-ignorant, planner will assume that in both environments, the MB-preferred action will follow (Fig 1B). Hence, this planner will anticipate a reward of 1 in each environment and will be indifferent between the two environments. In contrast, a self-reflective planner will anticipate the possibility of succumbing to a MF-inclination in the chosen environment (Fig 1C). In s1, this entails the risk that action L (for which the MB system anticipates a penalty) will be taken. On the other, hand, in s2 the desirable action R will be taken, even if MF tendencies dictate behaviour. Thus this planner will prefer a choice of s2. This example illustrates that when given an opportunity to choose a future environment it can be beneficial to take account of a possibility that future states might entail a supervention of one’s MF self.

Using a novel behavioural task we show, in support of our theory, that subjects tailor their environment consistent with the operation of self-reflective planning. In the Discussion, we consider wider implications of these findings with respect to real-life problems, such as drug abuse, and in relation to influential dual process theories in Psychology and Economics, including the tension between automatic and attentive (‘controlled’) processes [22,23].

## Results

Our bandit task allowed subjects to periodically design their environment by granting them ‘reward-setting’ choices, thereby allowing them exercise control over which bandits would be more, and which less, rewarding. Critically, the offered reward-settings were constructed such that a planner ignoring its own MF tendencies would be indifferent when provided with these reward settings. By contrast a self-reflective planner would show preferences that reflect an influence from its momentary MF tendencies.

**Fig 2:**
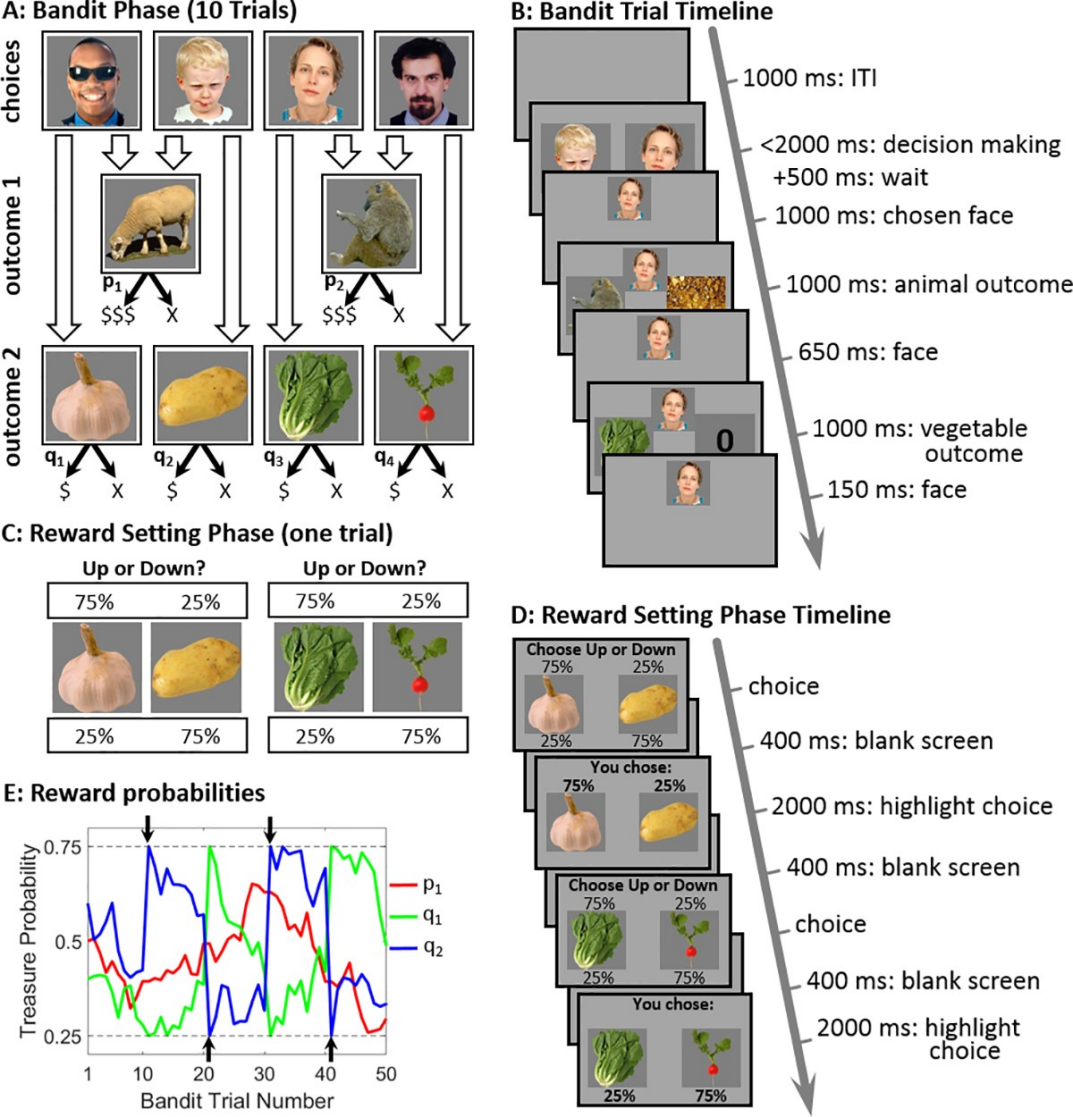
Task Structure. A) Participants were introduced to four individuals, where each had a preference for one animal and one vegetable. Thus, each vegetable was preferred by a single person while each animal was preferred by two individuals. When obtained as an outcome, animals and vegetables were probabilistically associated with a treasure worth 3-pt and 1-pt respectively (treasure probabilities denoted p_i,_ q_i_ in the figure). B) During bandit trials participants were given 2 seconds to choose one of two randomly-offered individuals, and subsequently obtained this individuals favourite animal (first) and vegetable (second). At this point, participants learnt, for each outcome, whether it provided a reward (in the current example trial the Monkey yielded a reward and Lettuce didn’t). Bandit phases consisted of 10 consecutive trials. C) Reward-setting phase. This phase iterated with bandit phases. A reward setting trial presented two vegetables preferred by individuals who shared a common animal preference (e.g., Garlic and Potato were preferred by Sheep-favouring individuals) and subjects were asked to set the associated reward-probabilities i.e., the chances of finding a treasure, to .75 for one of the vegetables and .25 for the other. D) Each reward-setting phase comprised two reward setting trials across which all 4 vegetable reward-probabilities were determined. Once these reward probabilities were set bandit trials resumed for 10 trials, and so on, until 300 bandit and 60 reward-setting trials were completed. Participants were asked to consider carefully, without time-limit, which settings best facilitate their future earnings upon resumption of bandit trials. E) Across trials, reward-probabilities for animals and vegetables drifted according to independent Gaussian random walks, with reflecting bounds at .25 and .75. Vegetable reward-probabilities were promptly adjusted according to chosen reward settings, but then continued drifting. The black arrows illustrate reward settings for Potato (the settings for the Garlic counterpart are symmetric). For clarity, only a single animal and one pair of the vegetables are presented for 50 (out of 300) trials.

We introduced participants to pictures of four different individuals and extensively trained them to learn the favourite animal and vegetable of these individuals. By design, each person favoured a unique vegetable and animal. Crucially, each animal was favoured by two of these four people (Fig 2A).

Following training, participants played 300 bandit trials each offering a 2-second limited choice between a randomly selected pair of individuals (Fig 2B). After choosing, participants received two outcomes—the chosen person’s favourite animal followed by their favourite vegetable. These outcomes were associated with gold (3 bonus pts.) and bronze (1 bonus pt.) treasure, respectively. Across the time-course of the experiment, the associated reward-probabilities (for animals and vegetables) were governed by independently drifting random walks (Fig 2E). As detailed below, common animal preferences allowed us to dissociate between MB and MF contributions to choices.

In our task design, following every set of 10 bandit trials, the bandit phase was paused and participants were asked two reward-setting questions (Fig 2C and 2D). Each question presented a pair of vegetables favoured by two individuals who favoured a common animal. Participants were asked to set the reward-probability to .75 for one of the vegetables in the pair, and .25 for the other, in a manner that could facilitate their earnings in following bandit trials (S1 and S2 Presentations for full task instructions). Thus, participants were able to determine which vegetables (but not which animals) would be more (and less) rewarding in subsequent trials. In so doing, participants could also consider (without time-limit) which reward setting was more likely to facilitate future performance on resumption of a set of bandit choices. These chosen reward-settings took immediate effect, but thereafter the associated reward-probabilities started to drift once more (Fig 2E). As explained below, reward-setting trials enabled us to probe whether chosen-settings reflected the possibility that agents took account of their MF tendencies towards the individuals (bandits) who favoured specific vegetables, a theoretical hallmark of a self-reflective planning.

### Model-free and model-based contributions to performance in bandit trials

We first assessed a MB and MF contribution to behaviour. We begin by specifying how both MF and MB systems contribute to the bandit-phase. Note that the rationale behind our analysis here is similar to the reasoning that has guided recent demonstrations of MF and MB signatures [14] (for similar approaches see also [7,20]). A MF system caches the long-term rewards associated with the previous choice of each person according to a Q-learning update scheme [24]. In the choice phase, the MF system feeds into a decision module retrieved Q^MF^-values of the persons (bandits) offered for choice. In the learning phase, the MF performs a Rescorla-Wagner [25] update with learning-rate *lr* based on a prediction error reflecting the total reward (animal + vegetable):

$$Q^{MF}(chosen\;person)\leftarrow Q^{MF}(chosen\;person)+lr*(total\;reward-Q^{MF}(chosen\;person))$$

Importantly, as the MF system lacks a model of task transition-structure its credit-assignment is restricted to the currently chosen person (bandit). Thus, for example, a sheep reward following a choice of the individual with sunglasses will update the Q^MF^-value of this person, but not that of the child, who happens also to favour the sheep (Fig 2A). The MB system, in contrast, uses a model of task transition structure to inform its choices. During the choice phase, the system calculates on-demand the Q^MB^-value for each offered individual (bandit) based on an arithmetic sum of the values of his or her preferred animal and vegetable:

$$Q^{MB}(person)=Q^{MB}(favorite\;animal)+Q^{MB}(favorite\;vegetable)$$

During the learning phase this system also uses Rescorla-Wagner [25] updates for the values of the observed animal and vegetable:

$$Q^{MB}(animal)\leftarrow Q^{MB}(animal)+lr*(animal\;reward-Q^{MB}(animal))$$

$$Q^{MB}(vegetable)\leftarrow Q^{MB}(vegetable)+lr*(vegetable\;reward-Q^{MB}(vegetable))$$

**Fig 3:**
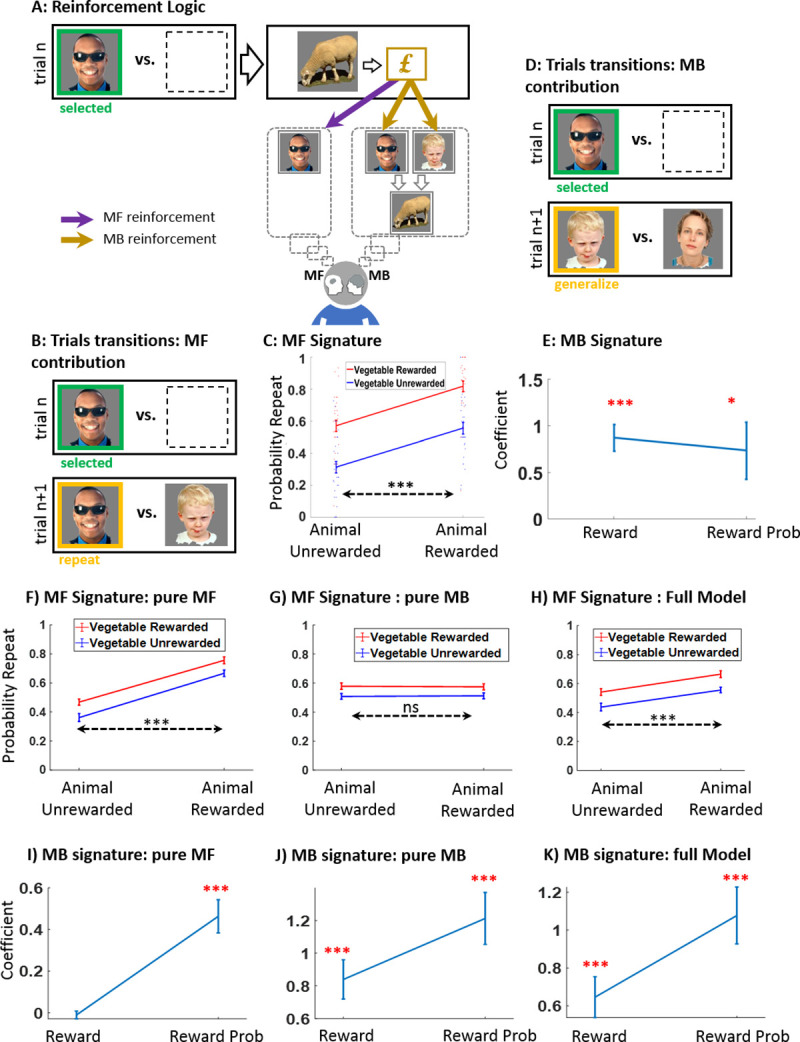
MF and MB contribution to bandit choices. A) After an individual (e.g., man with sunglasses) is selected, from a MF perspective an animal (e.g., sheep) outcome reinforces this chosen individual alone. In contrast, from a MB perspective, the animal outcome will affect equally the values of all individuals who prefer that animal, because the value of each of these individuals is the sum of the values of his (or her) preferred animal and vegetable (e.g., the MB value of the individual with sunglasses equals the sum of the values of *sheep* and garlic; similarly, the value of the child is the sum of the same *sheep*-value and potato-value). B) To test for a MF contribution to bandit choices we analysed trials that offered for choice the person chosen on the immediately preceding trial alongside the person who favoured the same animal. C) We tested the probability of repeating a choice as a function of the previous-trial animal and vegetable rewards. The effect of the trial-n animal outcome “cancels out” from MB trial n+1 calculations. Hence, an animal effect on choice-repetition isolates an MF contribution to bandit choices. For example, when the Sheep is rewarded vs. not-rewarded on trial n, the MF value of the man with sunglasses will be higher and the probability of repeating the choice will increase. D) To test for a MB contribution to bandit choices we analysed trials that excluded from choice the person chosen on the immediately preceding trial but offered the other person who favours the same animal. E) The probability of generalising a choice is higher when the animal was rewarded vs. non-rewarded on the immediately preceding trial after controlling for the animal reward probability—a signature of a MB contribution to bandit choices. F) A pure MF influence on bandit-choices sub-model predicted the main effect of animal on repetition probability. G) In contrast, a pure MB influence on bandit-choices sub-model did not predict a main effect of animal on repetition probability. H) Our full model, including mutual MF and MB influences on bandit choices predicted a main effect of animal. These simulation confirm that a MF contribution to bandit choices is necessary to account for the animal-reward effect on choice-repetition. I) A pure MF influence on bandit-choices sub-model failed to predict the positive main effect of animal reward on generalization probability after controlling for the generating reward-probability of the animal. J) In contrast, a pure MB influence on bandit-choices sub-model predicted the main effect of animal reward on generalization probability after controlling for the generating reward-probability of the animal. K) Our full model, including mutual MF and MB influences on bandit choice predicted this main effect with the control. These simulation confirm that a MB contribution to bandit choices is necessary to account for the positive animal effect or choice-generalization, controlling for the animal’s reward probability. The dots in panels C correspond to individual participants. Error bars correspond to SEM across participants calculated separately in each condition. ‘***’ and ‘*’ denote p < .001 and p < .05, respectively.

Unlike MF, MB animal credit-assignment generalizes across persons who share in common a favourite animal. Thus, when a sheep is rewarded this increases Q^MB^(sheep) and this will increase the calculated Q^MB^ -values for both the individual with sunglasses and for the child (Fig 3A).

Focusing on model-agnostic qualitative behavioural patterns we show both MF and MB contributions to bandit choices. We support these analyses with model-simulations of pure MF and pure MB contributions to bandit choices, whose main purpose is to validate the reasoning underlying our analyses. A full description of our model is provided in a later section (‘Computational Modelling’) and further details about model fitting and simulations can be found in the Methods (Model Fitting and Model Comparison; Model Simulations).

#### A model-free signature

Consider a focal trial n+1, which offers for choice the immediately preceding trial-n chosen person (e.g., man with sunglasses), alongside the other person who favours the same animal (e.g., child; Fig 3B). We tested whether the repetition probability for a choice on such trials depend on whether the animal (e.g., Sheep) was rewarded on trial n (controlling for the vegetable outcome). From the perspective of MB, the value of the animal, and in particular, whether it was rewarded previously, exerts no influence on the contrast between the Q^MB^-values of the offered individuals. This is because these individuals favour a common animal whose value affects their Q^MB^-values equally (note that a system’s contribution to choice depends on a contrast between the non-normalized values of the two persons). From the perspective of the MF system, however, an animal-reward on trial-n will preferentially reinforce the chosen person that led to that outcome alone and, as a consequence, increase the probability of repetition of that choice. A logistic mixed effects model, wherein we regressed the probability of repeating the trial-n choice on animal and vegetable trial-n rewards (Fig 3C) showed a main effect for animal-reward (b = 1.19, t(116) = 8.11, p = 6e-13), supporting a MF contribution (Fig 3F–3H for supporting model simulations). Additionally, we found a main effect for vegetable-reward (b = 1.35, t(116) = 7.69, p = 5e-12), predicted on the basis of both MF and MB contributions, but no significant interaction between reward types (b = 0.50, t(116) = 1.88, p>.05).

#### A model-based signature

Consider next a trial-n+1 which excludes the trial-n chosen person (e.g., the man with sunglasses; Fig 3D) from the choice set, but includes the other person who favours the same animal as the previously chosen person (e.g., child), a choice we label “generalization”. We examined whether the probability of generalizing the choice depended on the animal outcome (e.g., Sheep) on trial-n, controlling for the animal’s generative reward probability (we showed in previous work that this control is necessary to mitigate effects of auto-correlative reward sequences [14]). A MB contribution predicts a higher probability to generalize the choice when the animal was rewarded on trial-n, as compared to non-rewarded, because this reward increases the calculated Q-values of all persons (including the child) that favour that animal. In the case of the MF system, trial-n reward-events cannot causally affect choices on trial-n+1 because learning on trial-n was restricted to the chosen person, in the example the man with sunglasses who is not presented on trial n+1. A logistic mixed effects model showed (Fig 3E) a positive main effect for the animal-outcome (b = 0.87, t(2654) = 6.106, p = 1e-9) on choice-generalization, supporting an MB contribution to bandit choices (Fig 3I–3K for supporting simulations). Additionally, we found a significant main effect for the animal’s reward probability (b = 0.73, t(2654) = 2.396, p = .017) as predicted by both systems (Fig 3I–3K).

### Self-reflective planning in reward-setting phases

**Fig 4:**
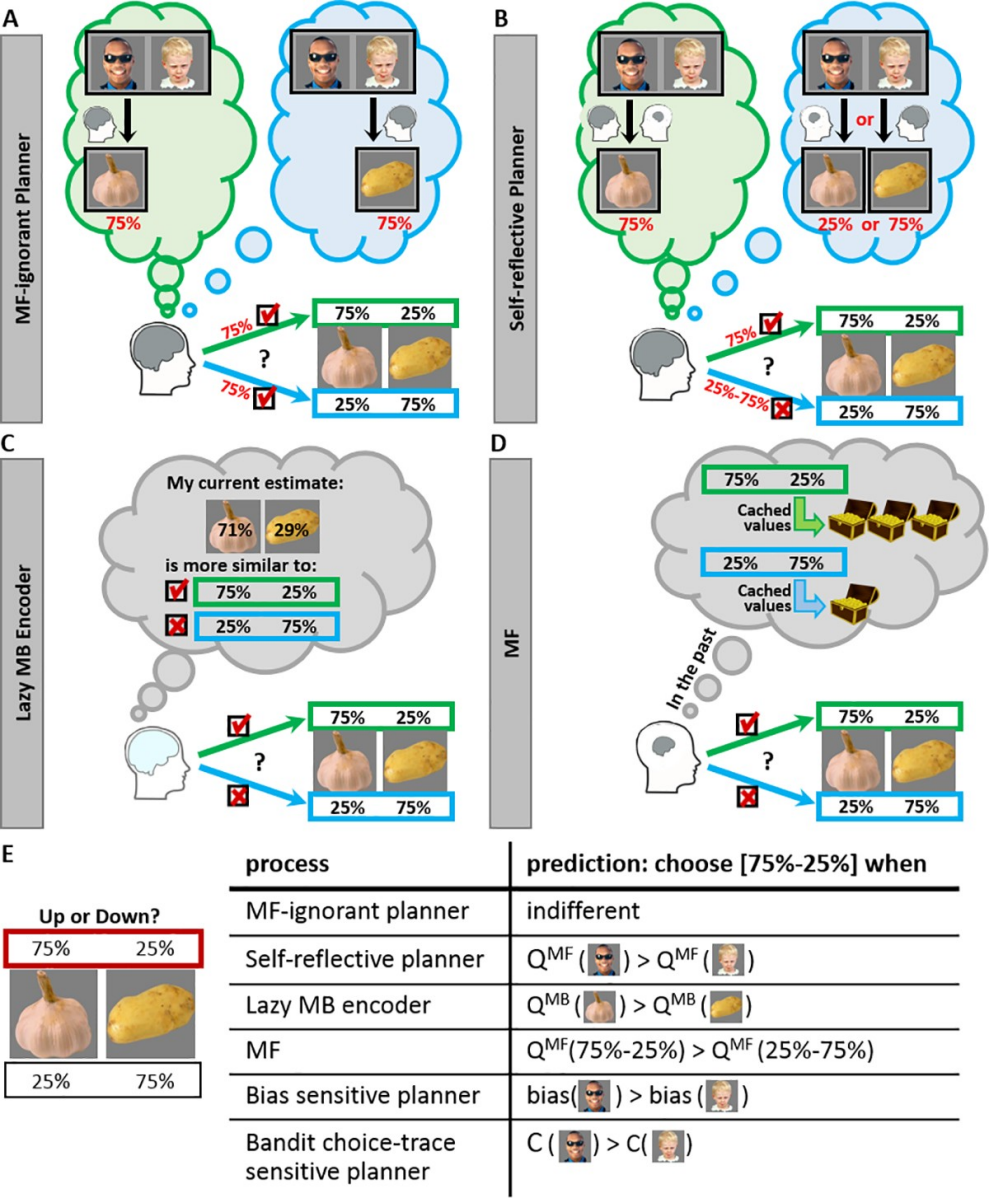
Illustration of contributions to reward setting trials. A) A MF-ignorant MB-planner evaluates the two offered reward settings. The planner simulates a prospective bandit trial offering, for example, the man with sunglasses and the child. Because both individuals prefer the very same animal, the planner assumes the agent will chose the person that provides the vegetable with the higher reward probability (Garlic or Potato), and in the example the agent will choose the man with sunglasses if Garlic is set to 75% reward-probability, or the child, if Potato is set to 75% reward-probability. Hence, the planner is indifferent between the two reward settings. B) In contrast, a self-reflective MB planners takes account of a momentary MF tendency to choose the man with sunglasses over the child. The planner realizes that if a MF system dictates an agent’s action in the aforementioned prospective bandit trial then the agent is more likely to choose the man with sunglasses and obtain the Garlic. Thus, the agent prefers a setting of 75% rather than 25% reward-probability for garlic. C) A lazy MB-encoder is also agnostic to MF tendencies but endures costs in encoding new vegetable-values and hence prefers the offered setting that is more similar to his/her current knowledge. D) A MF system prefers the reward-setting with the higher cached past value. These cached reward-setting values were learned from past experiences with these settings. E) A summary of the variables that will increase the probability that each process will tend to set Garlic to .75 and Potato to .25 reward probabilities: The self-reflective planner considers the Q^MF^-values of the individuals (bandits) who prefer the focal vegetables, the MF-ignoring planner is indifferent, the lazy encoder considers the Q^MB^-values of the focal vegetables when the setting is offered, the MF system considers the cached Q^MF^-values learned based on previous setting choices, the bias-sensitive planner considers the constant biases to choose the different bandits and finally, the bandit-choice-trace sensitive planner considers the bandit choice traces (i.e., relative recency-weighted choice frequencies for different bandits). See methods (‘computational models’) for full details pertaining to how biases and bandit choice traces were estimated.

Having shown a MF and MB contribution to bandit choices, we tested next our focal self-reflective planning hypothesis. Under our hypothesis, self-reflective MB planners consider how their momentary MF tendencies will affect their post reward-setting bandit-choices, and will choose a reward-setting that will be more profitable (contingent on MF supervening). In examining this hypothesis, we controlled for alternative hypothetical MB and MF influences on reward-setting behaviour (Fig 4).

To elaborate, consider first a benchmark Bellman planner (Fig 4A), who does not consider how their own MF-tendencies affect future bandit choices. This planner is *indifferent* in their choice between the two possible reward settings. This indifference is a consequence of our task-design wherein reward-setting questions always featured pairs of vegetables favoured by persons (bandits) with a common preference for the same animal. To illustrate, if the MB planner sets reward probabilities of Garlic to .75 and Potato to .25, then the MB values of the individuals who prefer these vegetables i.e., the man with sunglasses and the child, will update to Q^MB^ (sheep)+.75 and Q^MB^ (sheep)+.25, respectively. Similarly, if the MB planner applies the alternative setting then the values of the man with sunglasses and the child will update to Q^MB^ (sheep)+.25 and Q^MB^ (sheep)+.75, respectively. It follows that the sole difference between these two settings is an interchanging of the values of these two individuals (bandits). Because these two individuals are offered for choice in bandit trials equally often, the planner will expect equal earnings under both reward settings. Indeed, the MF-ignoring planner estimates their future earnings based on bandit values and is indifferent to bandit-identity (i.e., whether values are permuted between bandits).

Critically, the indifference between the offered reward-settings breaks down if one assumes a self-reflective planner takes account of MF contributions to their future bandit choices (Fig 4B). This reflective planner will then incorporate knowledge that subsequent bandit choices are subject to influences from an MF- bias to select the person with the higher Q^MF^-value (e.g., man with sunglasses), and hence receive their favourite vegetable (Garlic). Thus, the planner will infer that by setting the reward probability of garlic to .75, rather than .25, it can provide added insurance as to the fidelity of actions whose goal is greater expected future rewards. Thus, this planner will preferentially set the reward probability of .75 to the vegetable (Garlic) favoured by the person currently associated with a higher Q^MF^-value. Notably, this planner prefers a setting that facilitates a concordance, rather than conflict, between MB and MF tendencies towards bandit choices on subsequent trials (Fig 4B).

For completeness, we examined a possibility that, during reward settings, a planner reflects on two other potential non-MF self-biases operative at choice time (as reported in a following section, these biases were observed in our data). The first pertains to a constant bias to choose some bandits over others (e.g., a bias to choose the Child; note that because each person is associated with a unique vegetable, a bias to obtain a vegetable will manifest at choice time as a bias to choose the corresponding person). If in fact an agent is biased to choose some bandits over others, then a planner sensitive to these biases (henceforth a ‘bias sensitive planner’) will profit by setting high reward probabilities to vegetables that are associated with these favoured bandits. The second potential bias pertains to a reward-independent tendency to either perseverate or seek novelty during bandit trials (i.e. repeat or avoid recent bandit-choices irrespective of whether these choices were rewarded). If such biases are operative, then a planner can profit by reflecting on bandits’ choice-traces (a bandit’s choice trace corresponds to a recency-weighted measures of the frequency of past choices of that bandit relative to other bandits) and set rewards accordingly (henceforth a ‘bandit choice-traces sensitive planner’). Specifically, if an agent tends to repeat previous bandit choices then a planner will benefit by setting high reward probabilities to vegetables that are associated with these stronger choice-traces (and are therefore more likely to be chosen again). Vice versa, if an agent seeks novelty then a planner will profit by setting high reward probabilities to vegetables that are associated with bandits whose choice-traces are weaker (i.e., relatively ‘novel’ bandits).

In addition, we consider possible influences from a “lazy encoder’ MB planner (Fig 4C). Like a Bellman planner, a lazy encoder does not consider future MF influences on behaviour and expects to earn equally well in following bandit trials under both reward-settings. However, a lazy encoder will consider costs associated with updating the values of vegetables according to their chosen reward-setting. We assume the lazy encoder incurs a cost when encoding novel information that is proportional to the deviance between the novel to be encoded information, and the old currently encoded information. Thus, a lazy encoder prefers to set the vegetable with a currently higher Q^MB^-value to a 0.75 reward-probability. Note that lazy encoding can be construed as a planner’s attempt to maintain consistency between vegetable-values prior, and subsequent to, a reward setting, implemented by penalizing an offered reward setting based on the extent its inconsistency.

Finally, in addition to the above MB influences we considered direct MF contributions to reward settings i.e., MF tendencies towards the offered reward-settings (Fig 4D). We assume that for each reward-setting question, the MF system maintains two Q^MF^ values for the two possible settings, where rewards awarded in past bandit trials that followed a previously chosen setting served to reinforce it. For example, suppose a participant set the reward- probabilities of Garlic and Potato to .75 and .25 respectively. The higher the rewards earned on the following 10 bandit trials, the higher the MF tendency to repeat the setting rather than switch to the alternative setting, the next time settings are offered. In the following section we present model agnostic analysis as well as computational modelling that converge to show the contribution of a self-reflective planner in reward-setting trials.

### The effects of the preceding bandit-phase on chosen reward settings

**Fig 5:**
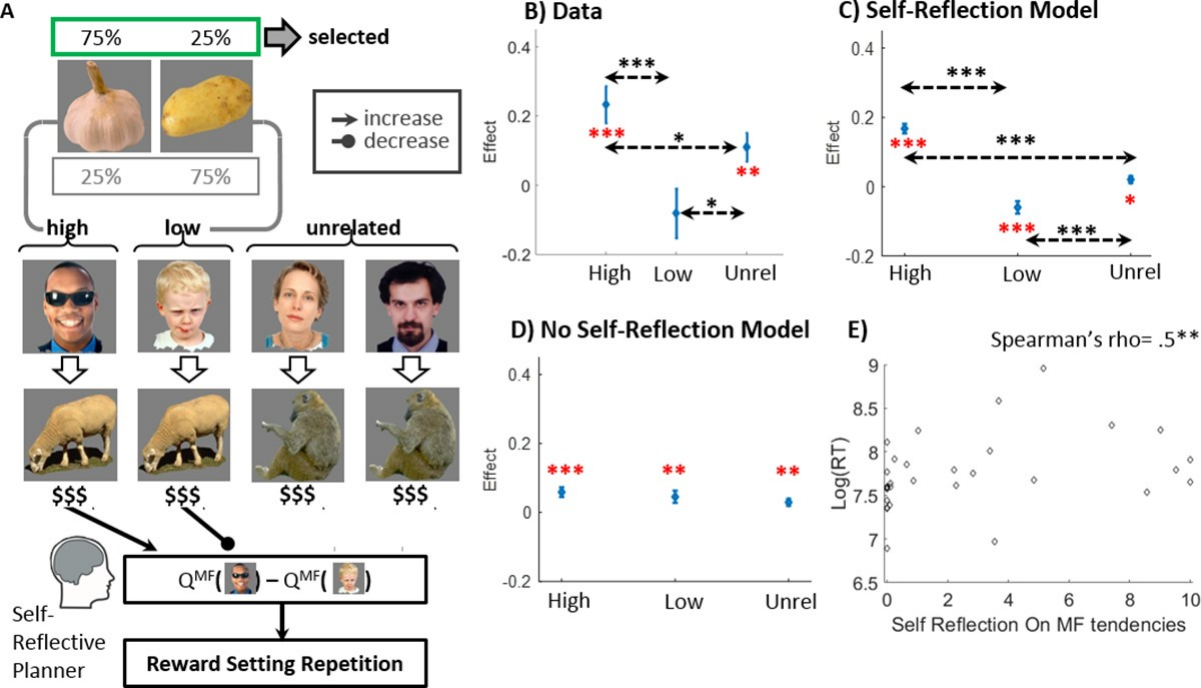
Testing a contribution from a self-reflective MB planner. A) Following a focal reward setting, bandit-trials are classified as high, low (the chosen person favours the vegetable that was set to reward probabilities .75 and .25, respectively) and unrelated (the chosen person favours a vegetable external to the focal setting). Animal rewards in high, low and unrelated trials increase, decrease and do not affect, the Q^MF^-value contrast between the persons favouring the setting-featured vegetables respectively. Consequently, from the perspective of a self-reflective planner (See Fig 4B and 4E), they increase, decrease or do not affect the self-reflective planner’s tendency to repeat the setting. Note that additionally, High, Low and Unrelated rewards all increase the tendency to repeat the reward setting via a non-displayed MF pathway (Fig 4D and 4E). B) Fixed effects for animals for our regression analysis. C) The full model, including contributions from a self-reflective planning process predicted a higher effect for Animal High than for Animal Unrelated and for Animal Low, and a higher effect for Animal Unrelated as compared to Animal Low. D) The ‘No self-reflective MB planning’ sub-model, which ablated contributions from a self-reflective planning process, did not predict any significant difference between the three animal effects. These model simulations confirm that a self-reflective MB component is necessary to account for our ‘model-agnostic’ signatures of self-reflective planning. E) Scatter plot of the contributions of a self-reflective planner to rewards settings, against log-median reward-setting RT (RT measured in msec). In B-D, Red ‘*’ denotes significant effects and black ‘*’ denotes significant tested contrasts. ‘*’,’**’, and ‘***’ correspond to p < .05, p < .01 and p < .001, respectively. If no asterisks appear, the animal effect is non-significant. Error bars correspond to standard errors.

In our model-agnostic analysis, we examined the effects animal-rewards, during the entire 10-trial bandit-phases, exert on an ensuing reward-setting-phase, specifically the decisions to repeat a previous reward setting or to switch it (Fig 5A). Following a reward setting, we classified the 10 following bandit trials into three types: either “High” and “Low”, wherein the choice (of a person) led to the vegetable set to .75 and .25 reward probability respectively, or “Unrelated” where the choice led to either of the other two vegetables external to this focal reward setting. For example, if Garlic and Potato were set to .75 and .25 reward-probabilities respectively, then in the following 10 bandit trials those that yielded Garlic (i.e., choices of man with sunglasses) were classified as “High”, those trials that yielded Potato (i.e., choice of child) were classified as “Low”, and trials that yielded either of the other two vegetables were classified as “Unrelated”.

Note that because vegetable reward probabilities drift after an enactment of a reward setting, it is possible that sometime during the 10-bandit trial phase following a reward setting, the vegetable that was previously set to a low reward-probability becomes superior (higher reward probability) to the vegetable previously set to a high reward probability. However, our bandit-trial taxonomy is not intended to imply that the “High” trials feature vegetables that are necessarily more rewarding than the “Low” trial vegetable during the entire 10-trial phase following a reward setting. Rather, our rationale for separating bandit trials into these three categories is that rewards obtained on these trial types exert different effects on a self-reflective planner’s tendency to repeat the previous reward setting. Indeed, as we now detail, rewards in high, low and unrelated trials increase, decrease or have no impact, respectively, on a self-reflective planner’s tendency to repeat the setting (Fig 5A).

To illustrate, and continuing our previous example, rewards obtained on High trials reinforce the MF value of the chosen person i.e., the man with sunglasses, alone (they do not affect the MF value of the child). Consequently, these rewards act to increase the contrast between the MF values of the man with sunglasses and the child. This in turn, increases the self-reflective planner’s tendency to set Garlic, rather than Potato, to a high reward probability on the following reward-setting (Fig 4B and 4E) i.e., repeat the previous reward setting. In contrast, rewards obtained on Low-trials, reinforce the MF value of the Child alone, hence decreasing the MF value contrast between the man with sunglasses and the child. This, in turns, decreases the self-reflective planner’s tendency to set garlic to a high reward-probability (i.e., repeat the previous reward setting) on the following reward setting. Finally, reward that are obtained on unrelated trials reinforce the MF value of neither the man with sunglasses nor the child. Thus, such rewards do not contribute to a tendency to repeat the previous Garlic/Potato reward setting.

Considering other pathways of influence on reward-settings trials, from a MF perspective (pertaining to reward-settings values; Fig 4D and 4E), *any* reward (as compared to non-reward) reinforces the last reward setting, thus increasing the probability of its repetition. This pathway, therefore, does not dissociate reward influences from high, low and unrelated trials. Furthermore, by focusing on rewards obtained for *animals* we mute contributions from a lazy encoder. Indeed, a lazy encoder’s tendencies towards reward settings are based on MB values of vegetables (Fig 4C and 4E), which are not affected by animal rewards (vegetable reward effects, in contrast, can be attributed to lazy encoding, see S1 Fig). Finally, as explained in the previous section, by task design, a Bellman planner is always indifferent between the offered reward settings (Fig 4A and 4E). In sum, there are two potential pathways via which High/Low/Unrelated animal reward can influence reward-setting repetitions: pure MF reward-setting values and self-reflective planning. Critically, only the latter dissociates these three effects predicting a High > Unrelated > Low effect-ordering.

Thus, we examined how the probability of repeating a previous reward setting depended on rewards gathered from animals, from these three trial-types (Fig 5B). Using a logistic mixed-effects main-effect model, we regressed the probability of repeating a reward-setting on the total reward received for animals in high, low and unrelated trials (Fig 5B). We found positive effects for both the high (b = 0.23, t(1736) = 4.34, p = 1e-5) and unrelated animal rewards (b = 0.11, t(1736) = 2.75, p = .006), but no significant effect for the low-animal rewards (b = -0.08, t(1736) = -1.13, p = .258). The positive unrelated-animal effect suggests a pure-MF contribution to reward-settings, whereby rewards accumulated during post-setting bandit trials reinforce the previous settings. Critically, we also found a significant difference between the three animal rewards (F(2,1736) = 5.51, p = .004). This difference was due to a larger effect for high vs. low animal-rewards (F(1,1736) = 10.97, p = 4.7e-4, one-sided), a larger effect for high vs. unrelated animal rewards (F(1,1736) = 3.47, p = .031, one-sided) and a smaller effect for low vs. unrelated animal rewards (F(1,1736) = 5.29, p = .011, one-sided), all of which support our self-reflective planning hypothesis. Importantly, our model simulations confirmed that a self-reflective planning process is necessary to account for the observed effect: whereas a model that included contributions from a self-reflective planner predicted the focal difference between animal effects related to different trial types (Fig 5C), a model without such contributions (but including contributions from a lazy MB encoder and MF tendencies) did not (Fig 5D). This validates both the rationale for our analysis and our full model [26].

In sum, our model agnostic analysis provided a signature of self-reflective planning. However, one caveat in this analysis pertains to its relative coarseness in aggregating rewards across an entire 10-trial bandit phase. Recall, during a reward setting choice, a self-reflective planner considers MF values of individuals corresponding to the focal vegetables. Notably, these MF values are affected to a larger extent by temporally recent rather than earlier choices of these individuals (because learning entails a decay of past values). Thus, rewards obtained closer to a reward setting will exert higher influence on a tendency to repeat a previous reward setting. However, to account for such influences necessitates an estimation of the rates that govern value-learning, using computational modelling. This limitation, therefore, is addressed in our following analyses.

### Computational modelling and model-comparisons

We formulated a computational model (henceforth the ‘full model’), which specified the likelihood of both bandit and reward-setting choices. We modelled bandit-choices as susceptible to a weighted mixture of MB-MF contributions, perseveration (vs. novelty seeking) tendencies and constants biases, and reward-setting choices as being affected by pure MF influences (i.e., cached reward-setting values), lazy encoding, a perseverance (vs. novelty seeking) tendency toward reward-setting, a self-reflective planning process, and sensitivity to constant bandit biases and bandits’ choice-traces. (Fig 4E). Our full model and sub-models of interest are fully described in the Methods (S1 Table for best-fitting parameters).

**Fig 6:**
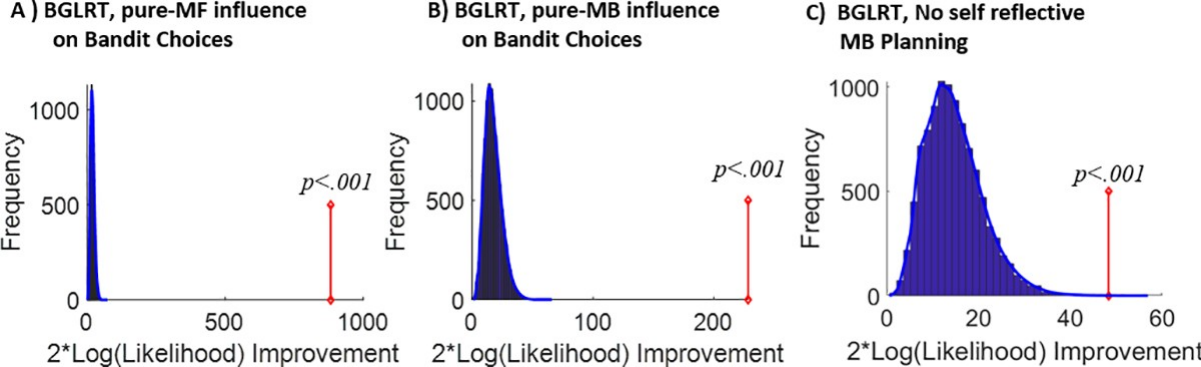
Model-comparison results. A) Results of the bootstrap-GLRT model-comparison for the pure MF-influence on bandit choices sub-model. The blue bars show the histogram of the group twice log-likelihood improvement (model vs. sub-model) for synthetic data that was simulated using the sub-model (10001 simulations). The blue line displays the smoothed null distribution (using Matlab’s “ksdensity”). The red line shows the empirical group twice log-likelihood improvement. 0 out of the 10001 simulations yielded an improvement in likelihood that was at least as large as the empirical improvement. Thus, the sub-model can be rejected with p < .001. B) Same as (A), but for the pure MB-influence on bandit choices sub-model. This sub-model was rejected with p < .001. C) Same as (A) but for the ‘No self-reflective MB planning’ sub-model. This sub-model was rejected with p < .001.

In testing the impact of the various potential factors we performed a set of model comparisons. For brevity, we report in the main text main findings related to our focal hypotheses (additional results are reported in S2 and S3 Figs). First, we formulated 2 sub-models of interest pertaining to contributions to bandit choices: 1) a ‘pure MB-influence on bandit choices’ sub-model, obtained by setting the contribution of the MF system to bandit choices to zero (i.e., *β*_*MF*_ = 0), and 2) and a ‘pure MF-influence on bandit choices’ sub-model, obtained by setting the contribution of the MB system to bandit choices to 0 (i.e., *β*_*MB*_ = 0). Our model comparisons were all based on a bootstrap generalized-likelihood ratio test [14,27] (BGLRT) between the full model and each of its sub-models in turn (Methods; S5 Fig). In brief, this method is based on classical statistics hypothesis testing, where in each of our model comparisons a sub model serves as the H0 null hypothesis and the full model as the alternative H1 hypothesis. We rejected both sub-models in favour of the full model (both p < .001) (Fig 6A and 6B). These results support a conclusion that both MB and MF systems contribute to bandit choices in our task. Additional model comparisons revealed that bandit choices were also influenced by both a novelty-seeking proclivity and constant (i.e., related to neither rewards nor choice-traces) biases (S2 Fig).

Next, we formulated an additional ‘no self-reflective planning’ sub-model, which excluded contributions of a self-reflective planner to reward-settings (*γ*_self ref_ = 0). Critically, this model was rejected in favour of the full model (p < .001; Fig 6C), supporting our main self-reflective MB-planning hypothesis. Remarkably, this analysis implicates a self-reflective MB-planning contribution after controlling for all the other considered modelled influences on reward-setting choices (i.e., pure MF-strategy, lazy encoding, etc.). To validate our full-model, we supplemented these model-comparison results by confirming based on model simulations that whereas our full model, which included a contribution from a self-reflective MB planner, accounted for empirical model-agnostic signatures (Fig 5B), the ‘No self-reflective MB planning’ sub-model did not [26] (Fig 5C and 5D). Further model validation simulations are reported in S6 Fig.

Additional model comparisons, showed that reward settings were also contributed by pure-MF tendencies, lazy-encoding and a tendency to choose novel reward settings (S3A–S3C Fig). Strikingly, model comparisons revealed that reward-settings reflected a sensitivity to non-MF bandit-related biases, i.e., constant and novelty-seeking biases (S3D and S3E Fig). Indeed, participants tended to set high reward-probabilities to vegetables, which they were biased to obtain (by choosing the corresponding bandits), because these vegetables were associated with bandits, which were in turn associated with stronger (constant) biases and/or weaker choice traces (higher novelty). One interpretation of these findings is that planners reflect, in addition to their own MF propensities, on other self-related choice-biases. Thus, planners consider their own constant or novelty-related biases to choose some bandits over others and profit by setting the corresponding vegetables to high reward settings. These exploratory findings expand the scope of self-reflective planning processes.

### Converging support for self-reflective planning based on reward-setting latencies

We reasoned that self-reflecting on one’s MF propensities would be a temporally demanding process, leading to a slowing in the latency of reward settings. As predicted, we found such a positive correlation (Spearman’s rho = .5, p = .005; Fig 5E) between participants’ median reward-setting RTs and the extent to which self-reflective planning contributed to one’s reward settings (as quantified by the full-model’s parameter γ_self ref_). Note that our estimation of the latter was based on choice data alone, and not on RTs. This finding provides converging support for a hypothesis that participants reflected on their MF tendencies when they set rewards.

### The relationship between MF and MB contributions to bandit choices and self-reflective MB contributions to reward-settings

In our task a hallmark of self-reflective planning is a consideration of MF tendencies towards bandits. A theoretical prediction is that a greater reliance on MF-tendencies in bandit choices entails a greater incentive to self-reflectively plan in reward-setting phases. Indeed, the more influential one’s MF-proclivities the more important it is to consider their likely influence while planning. To test this prediction, we quantified the absolute [28] (rather than relative) contributions of both the MF and MB systems to bandit choices as *β*_MF_ and *β*_MB_, respectively, based on each participants best fitting parameters (methods). In support of our hypothesis, we found a positive correlation (Spearman’s rho = .56, p = .002; S4A Fig) between the MF contribution to bandit choices and the extent of self-reflective planning (as quantified by the full-model’s parameter *γ*_self ref_). Additionally, we found a positive correlation between MB contributions to choices and self-reflective planning (Spearman’s rho = .40, p = .028; S4B Fig).

The above analyses raise a potential concern that spurious correlations could occur due to trade-offs in estimating different parameters (rather than real correlations in the population). To control for possible trade-offs between parameter estimates we performed a bootstrap parameter-trade-off simulation (methods) and found that the extent of self-reflective planning still correlated with MF contributions to bandit choices (p = .002), but not with MB contributions to choice (p = .06). We note the relationship between MB bandit-contributions and self-reflective planning may reflect the aggregated influence of two opposing contributions. On the one hand, as MB contributions to bandit choices increase (controlling for MF contributions) they dilute the relative influence of MF contribution to the same choices; thus the importance of self-reflection diminishes. On the other hand, a greater reliance on MB contributions in bandit choices may reflect more efficient MB-processes and hence a greater ability for MB self-reflection.

Finally, we found a positive correlation (S4C Fig; Spearman’s rho = .54, p = .002; after controlling for parametric trade-offs, p = .008; methods) between novelty seeking proclivities during bandit choices and a planner’s sensitivity to bandits’ choice-traces during reward settings (as quantified by the full-model’s parameters −*β*_C_ and -*γ*_*bandit trace*_, respectively; Methods). Thus, the stronger one’s tendency to choose novel bandits, the stronger his or her tendency to endow vegetables associated with novel-bandits with higher reward probabilities. These findings cohere with a notion that planners reflect also on their own novelty-seeking biases.

## Discussion

Previous evidence showing that biological agents rely on dual MF-MB systems [7,20] raises questions as to the nature and extent of system-interactions that govern overt behaviour. An extensive RL literature suggests these interactions are governed by diverse processes including a speed accuracy trade-off [29], trainer-actor dichotomy (or DYNA architecture)[8,30], MF reinforcement of MB-goals [13], reliability-based arbitration [31] and retrospective MB inference guiding MF credit assignment [14]. These varied perspectives on dual system-interactions concur on the importance of MF tendencies in shaping behaviour. Thus, it is surprising that theories of planning generally envision MB planners as agnostic to their own MF propensities. To the best of our knowledge, our study is the first to provide empirical evidence for a self-reflective planning process, wherein an agent’s MB system takes account of its MF tendencies during forward planning. More extensive research is needed to elucidate the formation, content and maintenance of such a self-model, as well as the cognitive and brain mechanisms that support it.

Self-reflective planning requires a detailed model of the self-as-actor, the forces that shape its behaviour, and the external (e.g., time pressure) and internal (e.g., cognitive load [32]) variables that operate on these forces. To evaluate current actions, a self-reflective planner consults its MF action-values and estimates the extent to which, and how, such contributions might influence its future actions. One hypothesis that emerges from our theory is that the extent of deployment of self-reflective planning is context-dependent. Thus, self-reflective planning can be expected to be enhanced whenever an agent anticipates future actions where these involve state characteristics that boost the relative contribution of MF-propensities to behaviour, for example stress [33] or cognitive load [32]. Understanding these potential contextual influences on self-reflective planning is an important direction for future studies. Such studies could usefully examine whether, and how, self-reflective planning relates to metacognitive abilities [34,35], or whether humans deploy simple heuristics [36] that bypass more complex processes so as to enable execution of self-reflective behaviours faster and using fewer cognitive resources [37].

An important question pertaining to self-reflective planning is whether a MB system has access to MF action-values. Recent evidence indicates this might be the case. For example, it has been shown that a MB system simplifies a complex planning process by restricting its depth, relying on surrogate MF evaluations for deeper states [38]. Our notion of a self-reflective planner offers a strikingly different view by proposing that a MB planner avails of MF action-values to calculate the potential consequences of MF-induced deviations from otherwise preferred plans. Thus, MF-considerations complicate, rather than simplify, the planning process as compared to a MF-agnostic planner. Importantly, rather than being mutually exclusive, these approaches could be combined in a self-reflective planning process within a limited-depth.

Evidence that people design their decision environment so that it aligns with their MF-proclivities is predicted by a “MF-MB conflict-agreement” principle. Choices between environmental-designs, as is the case for plans in general, should be penalized or prioritized based on the extent to which desired future MB actions conflict or align with MF tendencies. The utility of this design principle derives from a perspective of plan-reliability. A self-reflective planner realizes that a greater opposition between a plan and current MF proclivities increases the probability that a plan will not be followed. An alternative possibility is that a conflict between a desired MB-plan and MF-propensities will signal a need to recruit cognitively-costly executive control resources to ensure adherence to the plan. MB-MF consistency, on the other hand, can signal a greater reliability in plan implementation freeing control-processes for other needs. This view is reminiscent of an account of the “discipline of the will” in resolving a tension between instrumental actions and Pavlovian tendencies [39]. Future studies could usefully examine whether, and how, self-reflective planning is mediated by conflict-signals and the availability of control resources [40].

Dual control-process notions are ubiquitous in psychology and behavioural economics, where decisions are proposed to reflect a tension between automatic and attentive (‘controlled’) processes [22,23], deliberative and affective processes [41], long and short-term selves [42], or hot and cold modes of processing [43]. Similar self-reflective hypotheses can be derived in these frameworks (e.g., that a controlled planning process will take account of inferred automatic behaviour tendencies). Importantly, there are differences in the characteristics of the control processes that pertain to these diverse frameworks. Thus, deriving evidence in favour of self-reflective planning within these frameworks will require alternative task-designs and operationalisations. For example, within the framework of automatic vs. controlled processes, establishing an automatic habit requires substantial training, rendering the time-varying payoff structure implemented here (wherein bandits are chosen and rejected in rapid succession) inadequate.

In the present study, we focused on the MF system as an MB-external driver of behaviour, and we acknowledge additional influences on behaviour that can lead to plan-breaching, such as perseveration and novelty-seeking. Our exploratory findings show that planners reflected on a tendency to choose novel bandits and accordingly, designed their environment such that novel choices will be profitable. Pavlovian tendencies constitute another prevalent external influence [39]. We suggest that a self-reflective planner will also model an agent’s Pavlovian tendencies—an exciting hypothesis for future studies.

Future studies might usefully examine the developmental trajectory of self-reflective planning processes, including its relation to a social ability to deploy a theory of mind that allows agents to reliably predict the behaviour of others. A recent study has indeed shown that when observers predict the behaviour of other actors they take account of the actor’s habits, as well as the tension between the actor’s goals and habits [44]. Additionally, an influential theory in social psychology [45] suggests that people derive self-knowledge by “self-perceiving” themselves in a fashion similar to how they perceive others. An integration of these notions raises an intriguing possibility that self-reflective planning processes are based on an internalization of the notion that habits affect actors.

The theory of a self-reflective planner has importance for real-life problems such as substance abuse. Relapse, a major challenge to recovery from drug abuse, is defined by a return to drug use after a period of abstinence. Critically, the environment in which drug consumption occurs often triggers later reinstatement [46]. To prevent such cue-triggered relapse, former addicts are recommended to avoid exposure to drug-related contexts [47]. According to our theory, context-induced relapse can be understood as a combination, or mixture of, strong substance-related tendencies and a weak self-reflective planning process [48] that fails to take full account of maladaptive self- tendencies. In principle, a better understanding of conditions that foster self-reflective planning could lead to novel treatment approaches to addiction. Similarly, pre-commitment, i.e., the binding of one’s own behaviour, is a process by which the “cold” or “long term” self takes steps to prevent a “hot” or “myopic” self from making bad decisions [49]. This can be reinterpreted in our framework as an environmental design that minimizes anticipated conflicts between MF-tendencies and desired goals. We predict that manipulations that facilitate self-reflective planning would improve control over goal-thwarting tendencies through pre-commitment (e.g., controlling procrastination by setting deadlines [50]).

Finally, we acknowledge potential limitations of the current work. Our findings rely on a single experiment whose sample-size was not particularly large and utilised a relatively complex, and novel, reward-setting task. Thus, it will be important for future studies to both replicate current findings and provide converging evidence using alternative paradigms.

## Methods

### Ethics statement

The study was approved by the University College London Research Ethics Committee (Project ID 4446/001). Subjects gave written informed consent before the experiment.

### Experimental design

Forty one participants were recruited form the SONA subject pool (https://uclpsychology.sona-systems.com/Default.aspx?ReturnUrl=/) with the restrictions of having normal or corrected vision, being a London-based university student and being born after 1981.

Four participants reported repeating the same reward setting throughout the experiment to facilitate memory and were consequently excluded from analysis. Out of the rest, 7 participants were not significantly above chance performance on bandit trials (i.e., 55% accuracy over 300 choices; A bandit choice was defined as “correct” if the bandit with the objectively higher expected total reward was chosen) and were excluded from the analysis. We believe that this is a conservative criterion for exclusion, given that participant controlled the reward-probabilities in their reward-setting. The remaining 30 participants were the targets for our analysis.

These criteria for data exclusion (Performance not above chance; Always repeating the same reward setting) were pre-specified prior to data collection. Additionally, we pre-specified the desired sample size after exclusions (n = 30) and data collection terminated once this target number was obtained. Since our study is the first study to test for self-reflective planning in dual-system RL settings we collected a sample size considered large for such behavioural tasks. Our experiment was performed once.

The main objective of the study was to assess the contribution of a self-reflective MB system to reward-setting choices (see below). We hypothesized mutual MB and MF contributions in bandit-choices as well as self-reflective MB contributions in reward-setting choices. All these hypotheses were pre-specified prior to data collection.

### Experimental procedures

Participants were first introduced to four pictures of unique individuals and learned each person’s favourite animal and vegetable (the pictures of the individuals and their favourite animals and vegetables were adopted from previous studies [51,52]). Each person preferred a unique vegetable but preferred two animals. In turn each animal was preferred by two individuals. The mapping between persons and favourite objects was created randomly anew for each participant and remained stationary throughout the task. After learning, participants were quizzed about each person’s favourite animals and vegetable. Participants iterated between the learning and quiz stages until they achieved perfect quiz performance (100% accuracy and RT<3000 ms for each question).

After learning participants played 4 practice bandit trials, to verify their understanding of the task. These practice trials proceeded as described below with the sole difference that no time limit was imposed on a choice. They next played a single block of 48 bandit trials. On each trial, a pair of the four individuals were offered for choice (6 possible pairs were each repeated 8 times in a random order) and participants had 2 seconds to choose between one of these. Following a choice a favoured animal was displayed alongside a golden treasure (3 pts) or a “0” symbol (0 pts). Next, the favourite vegetable was displayed alongside a bronze treasure (1 pt.) or a “0”. The reward probabilities of the six animals and vegetables evolved across trials according to six independent Gaussian-increment random walk with reflecting boundaries at p = .25 and p = .75.

On completion of the first block participants were instructed about the reward-setting questions (S1 and S2 Presentations for full task instructions) and they then played a total of 300 bandit trials, divided into 6 blocks of 50 trials each. Following each sub-block of 10 bandit trials the bandit phase was paused and participants were asked two reward-setting questions. Each question featured a pair of vegetables that were favoured by individuals who favoured a common animal (the two questions thus covered the four vegetables). Participants were asked to choose (without time limit) whether they wanted to set the reward-probability of one of the vegetables to .25 and of the other to .75, or vice versa. After answering the 2 reward-setting questions the bandit trials resumed. The chosen reward settings were applied immediately but from that point onward continued to drift (independently) during the bandit phase until the next reward-setting phase. Participants had no control over animal reward-probabilities.

The task lasted about an hour. Participants were paid £7.5 per hour plus a performance based bonus (between £0–6). When the task was completed, 3 of the bandit trials were randomly selected and participants were paid £0.5 per bonus point earned on those trials.

### Statistical analysis

#### Model agnostic analysis

Our model-agnostic analyses were conducted using logistic mixed effect models (implemented with MATLAB’s function “fitglme”) with participants serving as random effects with a free covariance matrix. For the MF-contribution to bandit choices analysis (Fig 3B and 3C), we analysed only bandit trials n+1 that offered for choice the trial-n chosen person alongside the individual who favoured the same animal. We excluded trials (n+1) from the analysis if a reward-setting immediately preceded them (thus separating them from bandit trial n). Our regressors A (animal) and V (vegetable) coded whether the object provided a reward on the preceding trial-n (coded as +0.5 for reward and -0.5 for non-reward) and the regressed variable REPEAT indicated whether the choice on the focal trial n+1 was repeated. PART coded the participant contributing each trial. The model, in Wilkinson notation, was: REPEAT~ A*V + (A*V|PART).

For the MB-contribution to bandit choices analysis (Fig 3D–3F), we analysed only trials n+1 that excluded from choice the trial-n chosen person but offered the other individual who favoured the same animal. The regressors A and PART were coded as in the previous analysis and one additional regressor P coded the generating reward probability of the animal (we centralized this regressor by subtracting .5). The regressed variable GENERALIZE indicated whether the choice on the focal trial n+1 was generalized. The model, in Wilkinson notation, was: GENERALIZED~ A+P + (A+P |PART).

For our reward-setting analysis (Fig 5B), each reward setting split the following 10 bandit trials into 3 categories: High, Low and Unrelated, featuring the vegetable that was set to reward-probability .75, the vegetable that was set to reward-probability .25 and one of the two vegetables external to the setting, respectively (Fig 5A). We summed the rewards provided by animals (A) in the following 10 bandit trials for H/L/U trials to obtain 3 predictors (A_H/A_L/A_U). Rewards on single trials were coded prior to summation as -0.5 or +0.5. The dependent variable of interest REPEAT indicated whether the same reward setting was repeated following the 10 bandit trials. The mixed effect model we ran was REPEAT~ AH+AL+AU+(1|PART). We also ran a similar model but for the predictors (V_H/V_L/V_U) which summed vegetable rewards over the three trial types. Comparisons between the fixed effects related to H/L/U reward types were conducted using an F-test (using the ‘fixedEffects’ object function of the GeneralizedLinearMixedModel class in Matlab).

### Computational models

We formulated a reinforcement learning model to account for the series of bandit and reward setting choices for each participant. Bandit choices are contributed by both the MB and MF systems. The MF system caches a Q^MF^-value for each person, which is retrieved when the person is offered for choice. Following a choice the total reward (in points) from both the animal and vegetable is used to update the Q^MF^-value for the chosen person, based on a prediction error (with a free learning rate parameter 0≤*lr*≤1):

$$Q^{MF}(chosen\;person)\leftarrow Q^{MF}(chosen\;person)+lr*(total\;reward-Q^{MF}(chosen\;person))$$

the Q^MF^-values of the three non-chosen persons (the one that was rejected and the two who didn’t appear in the trial) decay to the default value of 2 (which is the expected total reward for choosing a person if animals and vegetables provide a treasure with probability .5; recall than an animal and vegetable treasures yield 3 and 1 point, respectively) with a free forgetting parameter 0≤*f*≤1

$$Q^{MF}(non\;chosen\;person)\leftarrow Q^{MF}(chosen\;person)+f*(2-Q^{MF}(chosen\;person))$$

The MB system maintains Q^MB^ values for the various animals and vegetables. During choice the Q^MB^ value for each offered person is calculated based on the transition structure

$$Q^{MB}(person)=Q^{MB}(favorite\;animal)+Q^{MB}(favorite\;vegetable)$$

Following a choice, the system updates the values of the observed animal and vegetable:

$$Q^{MB}(animal)\leftarrow Q^{MB}(animal)+lr*(animal\;reward-Q^{MB}(animal))$$

$$Q^{MB}(vegetable)\leftarrow Q^{MB}(vegetable)+lr*(vegetable\;reward-Q^{MB}(vegetable))$$

The values of the animal and three vegetables that were not observed decay to the default values of 1.5 and .5 (i.e., the expected value if reward probability is .5), respectively:

$$Q^{MB}(non\;obtained\;animal)\leftarrow Q^{MB}(non\;obtained\;animal)+f*(1.5-Q^{MB}(non\;obtained\;animal))$$

$$Q^{MB}(non\;obtained\;vegetable)\leftarrow Q^{MB}(non\;obtained\;vegetable)+f*(.5-Q^{MB}(non\;obtained\;vegetable))$$

Additionally, following a reward-setting choice the MB vegetable values for both vegetables featured in the setting are updated:

$$Q^{MB}(vegetable)\leftarrow set\;reward\;probability$$

Where "set reward probability" is .75 for one vegetable and .25 for the other.

We also included a gradual choice-perseveration (or novelty seeking) process in our model [53,54]. Each person (bandit) was associated with a choice-trace value *C*(person). Following a bandit-trial, all four (three non-chosen and one chosen) traces were updated according to:

C(nonchosenperson)←(1−τ)*C(nonchosenpersonperson)

C(chosenperson)←(1−τ)*C(chosenpersonperson)+τ

where 0≤*τ*≤1 is a parameter representing the decay rate of choice traces. These choice-traces affected choices as elaborated below.

Finally, we also model constant biases towards the four bandit. Since only bias- difference between pairs of bandits affected choices (see below), we set the bias towards one bandit to be *b*_1_ = 0, and biases to choose the 3 other bandits were free parameters, *b*_2_, *b*_3_, *b*_4_.

When a pair of persons are offered for choice the net Q value of each person is calculated as

$$Q_{net}(person)=\beta_{MB}*Q^{MB}(person)+\beta_{MF}*Q^{MF}(person)+\beta_{C}*C(person)+b(person)$$

Where *β*_*MB*_,*β*_*MF*_≥0 are free parameters that quantify the contribution of the MB and MF system, respectively to bandit choices. *β*_*C*_ is a free choice-trace weight parameter, which quantifies a general tendency to perseverate (*β*_*C*_>0) that is, select persons who were selected frequently on recent trials, or to seek novelty (*β*_*C*_<0; select persons who were not selected frequently on recent trials). Finally, *b*(person) is the bias parameters corresponding to that person.

The *Q*_*net*_ values for the offered person-pair are then injected into a softmax choice rule so that the probability to choose a person from the pair is:

$$Prob(person)=\frac{e^{Q_{net}(person)}}{e^{Q_{net}(person)}+e^{Q_{net}(other\;person)}}$$

Reward-setting choices, too, are contributed by MB and MF process. First, we assume that for each (of the 2) reward-setting questions, the MF system maintains a Q value (denoted *Q*^*MF*,*rs*^) for each of the possible two settings (e.g., V1:.75, V2:.25 or vice versa). Bandit trials that follow a chosen setting reinforce the setting. Following the bandit trial indexed *i* = 1,2,…,10 (after the last setting), a prediction error is calculated as *PE*_*i*_ = total reward−*Q*^*MF*,*rs*^(chosen setting) and is used to update the *Q*^*MF*,*rs*^ value of the last chosen setting:

*Q*^*MF*,*rs*^(chosen setting)←*Q*^*MF*,*rs*^(chosen setting)+lr*λ^*i*−1^*PE_*i*_ where 0≤*λ*≤1 is a free parameter describing the decay in learning rates as post-setting bandit trials progress. Note that the same 10 bandit trials are used to update the two chosen reward-settings (one for each of the two questions). Additionally, reward setting were also associated with choice-trace values denoted *C*_*rs*_. Following a reward setting trial choice traces values were updates for the chosen and rejected settings as:

$$C_{rs}(non\;chosen\;setting)\leftarrow (1-\tau )*C_{rs}(non\;chosen\;setting)$$

$$C_{rs}(chosen\;setting)\leftarrow (1-\tau )*C_{rs}(chosen\;setting)+\tau$$

When a reward setting question featuring two vegetable (V1,V2; favoured by persons P1 and P2, respectively) is offered, the net *Q* value for each setting is calculated based on the contribution of the different processes:

$$Q_{net}(V1:.75,V2:.25)=\gamma_{rs,MF}*Q^{MF,rs}(V1:.75,V2:.25)+\gamma_{lazy\;enc}*Q^{MB}(V1)+\gamma_{self\;ref}*Q^{MF}(P1)+\gamma_{C}*C_{rs}(V1:.75,V2:.25)+{\gamma_{bandit\;trace}*C(P1)+\gamma }_{bandit\;bias}*b(P1)$$

$$Q_{net}(V1:.25,V2:.75)=\gamma_{rs,MF}*Q^{MF,rs}(V1:.25,V2:.75)+\gamma_{lazy\;enc}*Q^{MB}(V2)+\gamma_{self\;ref}*Q^{MF}(P2)+\gamma_{C}*C_{rs}(V1:.25,V2:.75)+{\gamma_{bandit\;trace}*C(P2)+\gamma }_{bandit\;bias}*b(P2)$$

Where *γ*_*rs*,*MF*_≥0 is a free-parameter quantifying the contribution of the MF system to reward settings, *γ*_lazy enc_≥0 is a free-parameter quantifying the contribution of a lazy encoder to reward settings (note that a lazy encoder prefers setting the vegetable with the higher Q^MB^ value to reward-probability .75), *γ*_self ref_≥0 is a free-parameter quantifying the contribution of a self-reflective planner to the reward settings (note that a self-reflective planner prefers setting reward-probability .75 to the person who favours the vegetable with the higher Q^MF^-value), *γ*_C_ is a free reward-setting trace weight parameters which quantifies the tendency to perseverate (*γ*_C_>0) of seek novelty (*γ*_C_<0) in reward setting choices, *γ*_*bandit trace*_ is a free parameter quantifying the tendency to set high reward-probabilities to vegetables whose corresponding bandits are associated with strong (*γ*_*bandit trace*_>0) or weak (*γ*_*bandit trace*_<0) choice traces and *γ*_*bandit bias*_≥0 is a free parameter that quantifies a tendency to set high reward-probabilities to vegetables whose associate bandits are associated with stronger choice biases. (Note that the contribution of a pure MB-horizon (i.e., MF-ignorant) planner is not modelled, because they are indifferent between the reward settings). The *Q*_*net*_ values are then injected into a softmax choice rule so that:

$$Prob(V1:.75,V2:.25)=\frac{e^{Q_{net}(V1:.75,V2:.25)}}{e^{Q_{net}(V1:.75,V2:.25)}+e^{Q_{net}(V1:.25,V2:.75)}}$$

Q^MF^-values where initialized to 2 for each person and Q^MF,rs^-values were initialized to 0. Q^MB^ values were initialized to 0.5 for each vegetable and to 1.5 for each animal. Choice trace values were initialized to 0.25 for each bandit and to 0.5 for each reward setting.

### Model fitting and model comparison

We fit our models to the data of each individual using MATLAB’s ‘fmincon’, with 200 random starting points per participant. Because our models included many parameters (and were therefore prone to local minima and took considerable amount of time to fit) then rather than search for ML parameters over the entire parameter space we simplified the fitting method, fitting parameters in two waves. On Wave 1 we fitted the 9 “bandit” parameters (*β*_*MB*_,*β*_*MF*_,*β*_*C*_,*f*,*lr*,τ,*b*_2_,*b*_3_,*b*_4_) maximising likelihood over bandit trials. On wave 2 we fitted the 7 “reward-setting” parameters (*λ*, *γ*_self ref_, *γ*_*rs*,*MF*_, *γ*_lazy enc_, *γ*_C_, *γ*_*bandit trace*_, *γ*_*bandit bias*_) maximising likelihood over reward setting trials conditional on the Wave 1 fitted parameters.

Our full (Wave 1) model for bandit served as a super-model in a family of four nested “bandit” sub-models. The first, a ‘pure MB-contribution to bandit-choices’ sub-model constrained the MF contribution to bandit choices to 0, imposing *β*_*MF*_ = 0. The second, a ‘pure MF-contribution to bandit-choices’ sub-model, constrained the MB contribution to bandit choices to 0 imposing, *β*_*MB*_ = 0. The third, a ‘no bandit related perseveration’ sub-model constrained the bandit-trace weight parameter to 0, imposing *β*_*C*_ = 0. The fourth, a ‘no constant bias towards bandits’ sub-model constrained the 3 bias parameters towards bandits to 0 imposing, *b*_2_ = *b*_3_ = *b*_4_ = 0. Each of these models were tested against the full model using a BGLRT method (see below) based on likelihood in bandit choices. Because each of the sub-models were rejected in favour of the full model we augmented it at Wave 2 (with parameters *λ*, *γ*_self ref_, *γ*_*rs*,*MF*_, *γ*_lazy enc_, *γ*_C_, *γ*_*bandit trace*_, *γ*_*bandit bias*_).

The full reward-setting model also served as a super-model in a family of six nested sub-models: 1) a ‘no self-reflective MB planning’ sub-model constrained the contributions of the self-reflective MB planning process to 0, imposing *γ*_self ref_ = 0, 2) a ‘no lazy MB encoding’ sub-model constrained the lazy encoding contribution to 0, *γ*_lazy enc_ = 0. 3) a ‘no MF contributions towards reward-settings’ sub-model constrained the contributions of MF-tendencies towards reward-settings to 0, *γ*_*rs*,*MF*_ = *λ* = 0, 4) a ‘no perseveration towards reward setting” sub-model constrained the contributions the reward-setting trace weight to 0: *γ*_C_ = 0, 5) ‘bandit traces do not affect reward setting” sub-model constrained the contributions the reward-setting trace weight to 0: *γ*_*bandit trace*_ = 0. 6) a ‘constant biases towards bandit do not affect reward setting” sub-model constrained the reward-setting contributions of biases towards bandits to 0: *γ*_*bandit bias*_ = 0. Each of these models were tested against the full model using a BGLRT method (see below) based on likelihood to reward setting choices.

The bootstrapped generalized likelihood ratio test (BGLRT [27]) was conducted separately for each of the sub-models (vs. the super-model) (Fig 6). BGLRT is based on the generalized-likelihood ratio test (GLRT) but it computes the distributions for twice log-likelihood improvement (super vs. sub models) under the null hypothesis (defined by a sub-model) using a parametric bootstrap method in lieu of reliance on asymptotic Chi-squared distributions (as in GLRT). We preferred this bootstrap approach as we were uncertain as to the accuracy of the asymptotic approximation with a Chi-square distribution (especially for the case of reward settings for which we had ~60 ratings for participants). Each of our model comparisons postulated a sub model as the null (H0 hypothesis) with the full model serving as the alternative H1 hypothesis.

For each participant, we generated 1001 novel synthetic trial sequences (as in the actual experimental sessions). Simulating the sub-model on each of these sequences using the participant’s best-fitting ML parameters, we obtained 1001 synthetic datasets per participant. Each of these synthetic datasets were next fitted using both the super-model and the sub-model (when fitting reward-setting models we used the two-wave method as for the empirical data) and we calculated the improvement in twice the logarithm of the likelihood for the full model. When fitting a sub-model we use the generating parameters as starting point, and when fitting the full model- we converted the best fitting parameters of the sub-model to a starting point of the full model (in two wave fitting, the generating bandit-model parameters of the full model where used as starting point for the first wave). Thus, we obtained 1001 (twice log) likelihood improvements per participant. Next, we repeated the following 10,000 times. We sampled randomly, and uniformly, one synthetic twice log-likelihood super-model improvement per participant (from the set of 1001 improvements) and these samples were summed across participants. These 10,000 obtained values comprise the null distribution of group super-model likelihood improvement. Finally, we calculated the p-value for rejecting H0 at the group level as the proportion (of these 10,000) values that were at least as large as the empirical aggregate twice log-likelihood improvement (super vs. sub-model).

### Model recovery

Because our model-comparisons are based on BGLRT, model recovery questions are tantamount to assessing type-I error rates and power. We used a type I error rate of .05 meaning that by design, if a null model (one of our sub-models) generated the data it will not be rejected (i.e., it will be “recovered”) with a probability of .95. To estimate the power of our design, we repeated the same steps as in our BGLRT analysis, but this time using synthetic data simulated from the full model instead of the sub-models. For each simulated data set, we examined whether each of the null models were rejected, according to BGLRT, in favour of the full model. We found that for all 10,000 simulations, all bandit-trial sub models were rejected in favour of the full model (i.e., the full model was “recovered”) at the group level. Thus the estimated power is very close to 1. For the reward setting models the estimated power was > = .92 for all sub-models (S5 Fig).

We also examined model recovery using other model-comparison methods. The first was traditional GLRT, which is identical to BGLRT but where significance is estimated based on the Chi-square asymptotic distribution assumption (with degrees of freedom = number of participants x difference in number of free parameter between the full model and a sub-model) instead of the bootstrap method. As seen in S5 Fig, this method provided excellent power for rejecting the bandit sub-models, but for some of the sub-models it yielded very high type-I error rates. Furthermore, this method provided either poor power or high type I error rates for 3 out of the 6 of the reward-setting sub-models. We also examined AIC and BIC, by repeating our method (i.e., fitting both a sub-model and super model to synthetic datasets generated by either model) but summing AIC or BIC differences (between the full model and a sub-model) across participants rather than twice the log-likelihood, and selecting the model with the lower aggregate score. As seen in S5 Fig, AIC provided excellent model recovery for the bandit trial models, but for 3 of the reward-setting models it was too conservative, i.e., it recovered the sub-model with high probability even when the full model generated data. Finally, BIC was clearly too conservative, especially for reward-setting models: All sub-models were selected on a majority of simulations even when the super-model generated the data. In fact, for 5 out of the 6 sub-models the super model was recovered with probability < = .0005.

### Model simulations

To generate model predictions (Figs 3 and 5 and S1), we simulated for each participant, 15 synthetic experimental sessions (novel trial sequences which were generated as in the actual experiment), based on his or her best fitting parameters obtained from the corresponding model fits (the models are described above). We then analysed these data in the very same way as the original empirical data (but with datasets that were 15 times larger, as compared to the empirical data, per participant).

For the model-validation simulation (S6 Fig) we used the same method to simulate data from the full and from the no self-reflection models but with one experimental session per participants. We next subjected the 30 synthetic datasets to the mixed effects model (for Animals) used for Fig 5B to obtain synthetic High/Low/Unrelated fixed effects. We repeated this entire procedure 1001 times.

### Parametric trade-off bootstrap simulation

Our correlation calculations in the section ‘The Relationship between MF and MB Contributions to Bandit Choices and Self-Reflective MB Contributions to Reward-Settings’ are subject to a concern that spurious correlation will occur due to trade-offs in estimating different parameters (rather than real correlations in the population).To control for parameter trade-offs we performed a bootstrap simulation as follows. We constructed a group of 1200 pseudo-participant by cloning each of our 30 participants (with his or her estimated parameters) 40 times. Next, we permuted separately each of the model parameters across all 1200 pseudo participants. This ensured that there were no correlations between any parameters in this pseudo-population. We then generated a simulated data set (choice and reward settings) for each pseudo participant based on his/her parameters. Next, we repeated the following procedure 100,000 times: 1) we sampled without replacement a group of 30 pseudo participants, 2) We fitted our full model to each participant’s simulated choices and reward-settings (in the same way as we fitted the empirical data) and 3) we calculated pairwise correlation of interest (a correlation between MB (or MF) contributions to bandit-choices and the extent of self-reflective planning; a correlation between novelty seeking proclivities during bandit choices and a planner’s sensitivity to bandits’ choice-traces during reward settings). These 100,000 correlations (for each pair of contributions) defined a null distribution for evaluating the statistical significance of the corresponding empirical correlation. Specifically, the p-value of the empirical correlation was calculated as the proportion (of 100,000) pseudo groups, for which the correlation was larger or equal to the empirical correlation. If the p value was larger than .5 we replaced it by 1-p. Finally, we multiplied the p-values by 2 to obtain 2 sided p-val.

## Supporting information

S1 Fig Related to Fig 5A. The findings for vegetables were similar to the findings for animal reported in the main text: we found a positive effect for the high-vegetable reward (b = 0.24, t(1736) = 4.33, p = 2e-5) and non-significant effects for the low (b = -0.11, t(1736) = -1.42, p = .156) and unrelated (b = 0.08, t(1736) = 1.88, p = .06) vegetable-reward. The effects for the three vegetable types differed (F(2,1736) = 6.97, p = 9.6–4) and were driven by a stronger effect for high vs. low (F(1,1736) = 13.75, p = 1e-4, one sided) and unrelated (F(1,1736) = 4.89, p = .014, one-sided), and a stronger effect for unrelated vs. low (F(1,1736) = 4.92, p = .013, one-sided). While supporting the self-reflective hypothesis, these results could potentially be attributed to a lazy MB encoder because high, low and unrelated vegetable rewards increase, decrease or do not affect, respectively, the relative Q^MB^-values of the vegetable that was last set to high reward-probability and its counterpart (Fig 4C and 4E). B) Our full model, including a self-reflective planning process predicted the empirical difference between the 3 vegetable conditions. C) Differences between the High and Low and between the High and Unrelated vegetables were also predicted the ‘no self-reflective MB-planning’ sub-model (which included a lazy encoder component). The structure of this Figure is same as Fig 5B.
(TIF)

S2 Fig Related to Fig 6. We formulated 2 additional sub-models of interest with respect to bandit choices: 1) a ‘no bandit related perseveration (or novelty-seeking)’ sub-model which allowed for neither perseveration nor novelty-seeking tendencies towards bandits and, 2) a ‘no constant bias towards bandits’ sub-model, which excluded constant biases to choose bandits (See Methods for full details). A) The ‘no bandit related perseveration (or novelty seeking)’ sub-model was rejected (p < .001). Furthermore, the full-model’s parameter quantifying the perseveration vs. novelty-seeking tendency to choose bandits (*β*_*C*_), was significantly negative (Wilcokxon signed rank test, median = -1.62, z = -2.66, p = .008). These results show that in addition to MB and MF contributions, bandit choices were influenced by a novelty-seeking proclivity. B) Same as A but for the ‘no constant biases sub-model (p < .001). Thus, bandit choices were influenced by both constant biases The structure of this figure is similar to Fig 6.
(TIF)

S3 Fig Related to Fig 6. We formulated 5 additional sub-models of interest pertaining to putative contributions to reward settings. Each of these sub-models eliminated in turn one of these putative influences. Thus we obtained a 1) a ‘no MF influences on reward settings’ (*γ*_*rs*,*MF*_ = 0), 2) a ‘no-lazy encoder’ (*γ*_*lazy enc*_ = 0), 3) a ‘no perseveration towards reward settings’ (*γ*_*C*_ = 0), 4) a ‘no sensitivity to bandit related constant biases’ (*γ*_*bandit bias*_ = 0), and 5) a ‘no sensitivity to bandit choice-traces’ sub-model (*γ*_*bandit trace*_ = 0). See Methods for full details. A) The ‘no MF tendencies towards reward-settings’ sub-model was rejected (p = .032) in favour of the full model, showing that reward settings were influenced by cached MF reward-setting values. B) Same as (A) but for the ‘no lazy encoding’ sub-model (p = .024). These results showed that reward-settings were influenced by lazy encoding. C) Same but for the ‘no perseveration (or novelty-seeking) towards reward-setting’ sub-model (p < .001). Additionally, the full-model’s parameter, quantifying the perseveration vs. novelty-seeking tendency towards reward settings (*γ*_C_) was significantly negative (Wilcokxon signed rank test, median = -3.34, z = -3.10, p = .002), showing that participants were novelty seeking in their reward-setting choices (i.e., they tended to choose reward setting which were chosen less frequently in the recent past). D) Same but for the ‘no sensitivity to bandit related constant biases’ sub-model (p < .001), E) same but for the ‘no sensitivity towards bandit-related choice traces’ sub-model (p = .0051). The structure of this figure is similar to Fig 6.
(TIF)

S4 Fig Scatter plots for correlations between model parameters. A) The correlation between MF contributions to bandit choices (*β*_MF_; abscissa) and the self-reflective planner’s contributions to reward-setting choices (*γ*_self ref_; ordinate). B) The correlation between MB contributions to bandit choices (*β*_MB_; abscissa) and the self-reflective planner’s contributions to reward-setting choices (*γ*_self ref_; ordinate). C) The correlation between perseveration tendencies towards bandit choices (*β*_*C*_; abscissa) and the contributions of self-reflection on perseveration tendencies towards bandits (*γ*_*bandit trace*_; ordinate). Note that negative values of perseveration correspond to novelty seeking and negative values of reflection correspond to the tendency to assign high reward probability to vegetables associated with novel bandits. Each diamond corresponds to an individual participant.
(TIF)

S5 Fig Comparison of Model Comparison Methods. A) The proportion of simulations (out of 10,000; Methods) in which the full bandit model is selected at the group level over each sub-model, when data is generated either from the corresponding sub-model (blue asterisks) or from the full model (red diamonds) for different model-comparison methods. Note that for BGLRT and GLRT, selection probabilities corresponds to type I errors (when a sub-model generated data) or to power (when the full model generated data). For AIC and BIC, selection probabilities corresponds to a lower sum of the corresponding information criterion across participants. The sub-model label refers to the component that was ablated from the full model. MB: pure MF contributions to bandit choices sub-model; MF: pure MB contributions to bandit choices sub-model; B: no constant Bias towards bandits sub-model; P: no bandit related Perseveration sub-model. B) Same as (A) but for the reward-setting models. SF: No Self Reflective MB planning sub-model; P: no Perseveration towards reward setting sub-model; LE: no Lazy MB Encoding sub-model; MF: no MF contributions towards reward-settings; BT: Bandit Traces do not affect reward settings sub-model; B: constant Biases towards bandit do not affect reward settings sub-model.
(TIF)

S6 Fig Model Validation Simulation. In the main text figures (Fig 5C and 5D) we generated model predictions based on 15 synthetic data sets per subject. While this methods allows noise reduction in calculations of model predations it renders a comparison between empirical and simulated effects difficult because it does not allow an assessment of how likely empirical effects will be generated by the models. Thus, we examined in greater detail the distribution of model predictions when a single dataset is generated per participant. A) We simulated for each participant a single synthetic experimental session (novel trial sequences which were generated as in the actual experiment), based on his or her best fitting parameters from the full model. We then repeated the mixed effects model reported in the section “The Effects of the Preceding Bandit-Phase on Chosen Reward Settings” for these synthetic data (i.e., for 30 synthetic participants). Thus, we obtained synthetic fixed effects for High/Low/Unrelated trials (as in Fig 5B). This entire procedure was repeated 1,001 times. Left panels present scatter plots for each pair of fixed effects (blue circles) and the empirical fixed effects (black *). Right panels present distributions for corresponding synthetic effect-contrasts (blue; estimated using Matlab’s routine “ksdensity”) and the empirical contrast (black). The titles reports the proportion of simulations which generated a contrast that is at least as large as the empirical contrast. The full model tends to generate positive contrasts and the empirical results fall comfortably within the range of model-simulations. B) Same but for the “No self-reflection sub-model”. This model predicts contrasts that are distributed around 0, and the empirical contrasts (especially High vs. Low, Other vs. Low) are very unlikely to emerge from this sub-model.
(TIF)

S1 Table Best fitting parameters for the RL model. The table shows the 25, 50 and 75 percentiles across participants. (See ‘Computational Models’ in methods for a full description of the model and its parameters).
(PDF)

S1 Presentation Task instructions part 1. (PPTX)

S2 Presentation Task instructions part 2. (PPTX)
