# Supplementary figures and images for: Model based planners reflect on their model-free propensities
Source: PLoS Comput Biol. 2021 Jan 7;17(1):e1008552. doi: 10.1371/journal.pcbi.1008552 (PMC7817042; doi:10.1371/journal.pcbi.1008552)

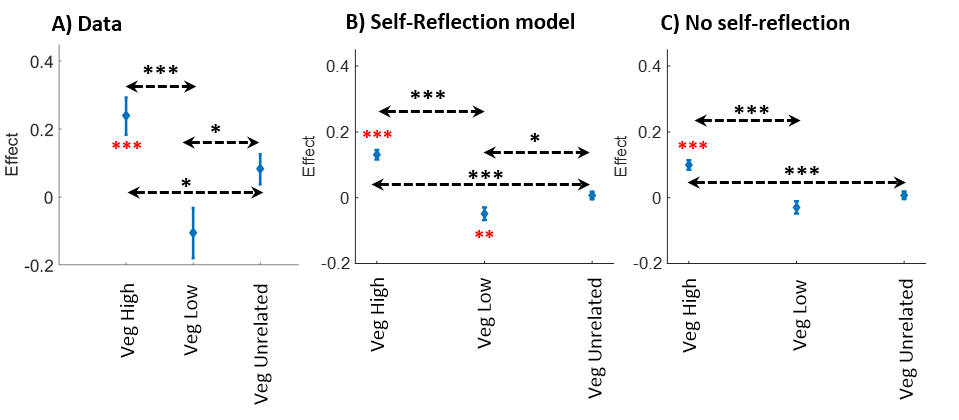

Supplement: S1 Fig — The findings for vegetables were similar to the findings for animal reported in the main text: we found a positive effect for the high-vegetable reward (b = 0.24, t(1736) = 4.33, p = 2e-5) and non-significant effects for the low (b = -0.11, t(1736) = -1.42, p = .156) and unrelated (b = 0.08, t(1736) = 1.88, p = .06) vegetable-reward. The effects for the three vegetable types differed (F(2,1736) = 6.97, p = 9.6–4) and were driven by a stronger effect for high vs. low (F(1,1736) = 13.75, p = 1e-4, one sided) and unrelated (F(1,1736) = 4.89, p = .014, one-sided), and a stronger effect for unrelated vs. low (F(1,1736) = 4.92, p = .013, one-sided). While supporting the self-reflective hypothesis, these results could potentially be attributed to a lazy MB encoder because high, low and unrelated vegetable rewards increase, decrease or do not affect, respectively, the relative QMB-values of the vegetable that was last set to high reward-probability and its counterpart (Fig 4C and 4E). B) Our full model, including a self-reflective planning process predicted the empirical difference between the 3 vegetable conditions. C) Differences between the High and Low and between the High and Unrelated vegetables were also predicted the ‘no self-reflective MB-planning’ sub-model (which included a lazy encoder component). The structure of this Figure is same as Fig 5B. (TIF) [file pcbi.1008552.s001.tif]

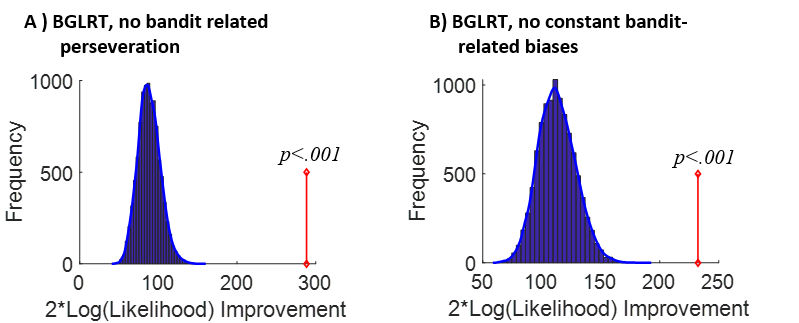

Supplement: S2 Fig — We formulated 2 additional sub-models of interest with respect to bandit choices: 1) a ‘no bandit related perseveration (or novelty-seeking)’ sub-model which allowed for neither perseveration nor novelty-seeking tendencies towards bandits and, 2) a ‘no constant bias towards bandits’ sub-model, which excluded constant biases to choose bandits (See Methods for full details). A) The ‘no bandit related perseveration (or novelty seeking)’ sub-model was rejected (p < .001). Furthermore, the full-model’s parameter quantifying the perseveration vs. novelty-seeking tendency to choose bandits (βC), was significantly negative (Wilcokxon signed rank test, median = -1.62, z = -2.66, p = .008). These results show that in addition to MB and MF contributions, bandit choices were influenced by a novelty-seeking proclivity. B) Same as A but for the ‘no constant biases sub-model (p < .001). Thus, bandit choices were influenced by both constant biases The structure of this figure is similar to Fig 6. (TIF) [file pcbi.1008552.s002.tif]

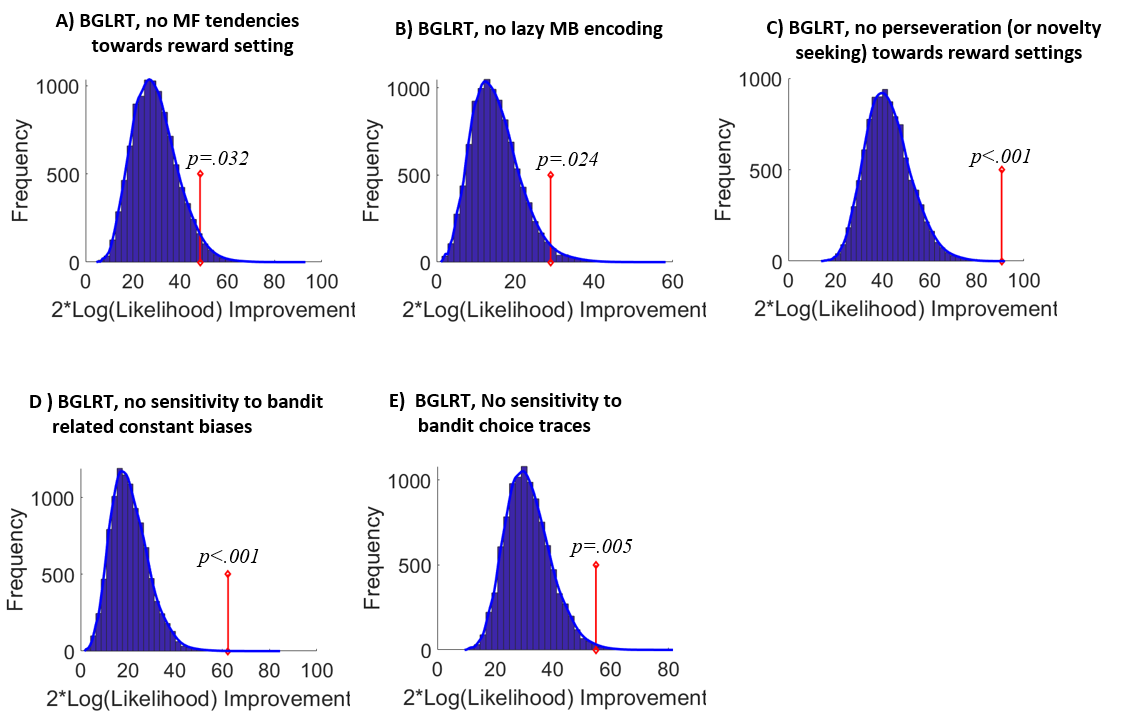

Supplement: S3 Fig — We formulated 5 additional sub-models of interest pertaining to putative contributions to reward settings. Each of these sub-models eliminated in turn one of these putative influences. Thus we obtained a 1) a ‘no MF influences on reward settings’ (γrs,MF = 0), 2) a ‘no-lazy encoder’ (γlazy enc = 0), 3) a ‘no perseveration towards reward settings’ (γC = 0), 4) a ‘no sensitivity to bandit related constant biases’ (γbandit bias = 0), and 5) a ‘no sensitivity to bandit choice-traces’ sub-model (γbandit trace = 0). See Methods for full details. A) The ‘no MF tendencies towards reward-settings’ sub-model was rejected (p = .032) in favour of the full model, showing that reward settings were influenced by cached MF reward-setting values. B) Same as (A) but for the ‘no lazy encoding’ sub-model (p = .024). These results showed that reward-settings were influenced by lazy encoding. C) Same but for the ‘no perseveration (or novelty-seeking) towards reward-setting’ sub-model (p < .001). Additionally, the full-model’s parameter, quantifying the perseveration vs. novelty-seeking tendency towards reward settings (γC) was significantly negative (Wilcokxon signed rank test, median = -3.34, z = -3.10, p = .002), showing that participants were novelty seeking in their reward-setting choices (i.e., they tended to choose reward setting which were chosen less frequently in the recent past). D) Same but for the ‘no sensitivity to bandit related constant biases’ sub-model (p < .001), E) same but for the ‘no sensitivity towards bandit-related choice traces’ sub-model (p = .0051). The structure of this figure is similar to Fig 6. (TIF) [file pcbi.1008552.s003.tif]

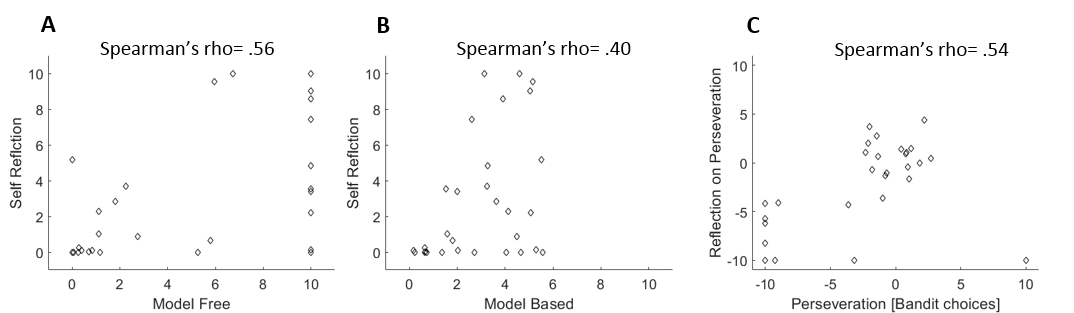

Supplement: S4 Fig — A) The correlation between MF contributions to bandit choices (βMF; abscissa) and the self-reflective planner’s contributions to reward-setting choices (γself ref; ordinate). B) The correlation between MB contributions to bandit choices (βMB; abscissa) and the self-reflective planner’s contributions to reward-setting choices (γself ref; ordinate). C) The correlation between perseveration tendencies towards bandit choices (βC; abscissa) and the contributions of self-reflection on perseveration tendencies towards bandits (γbandit trace; ordinate). Note that negative values of perseveration correspond to novelty seeking and negative values of reflection correspond to the tendency to assign high reward probability to vegetables associated with novel bandits. Each diamond corresponds to an individual participant. (TIF) [file pcbi.1008552.s004.tif]

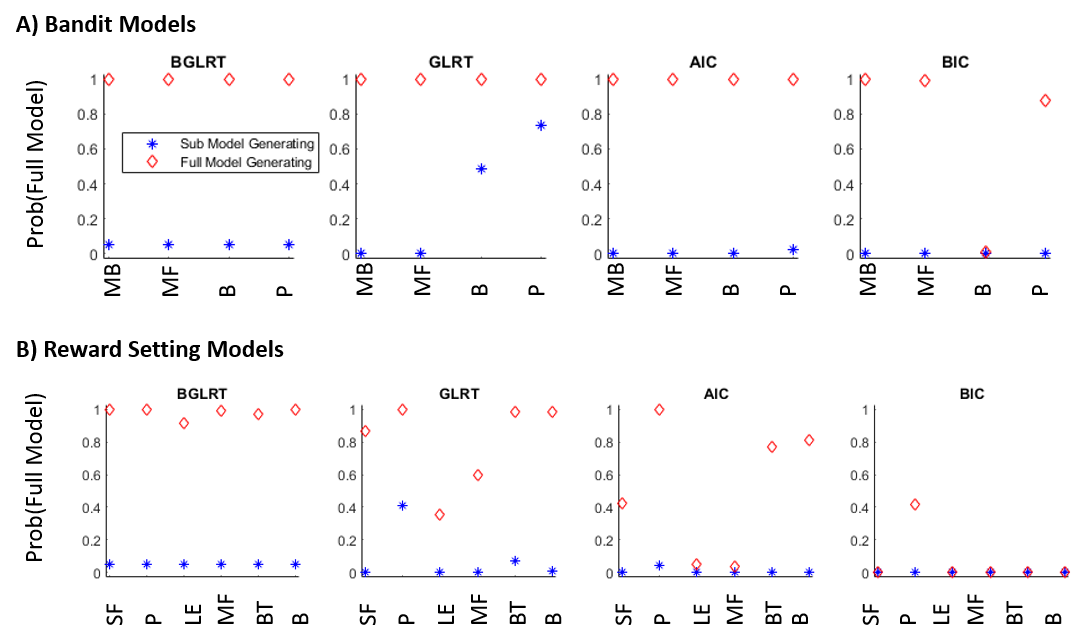

Supplement: S5 Fig — A) The proportion of simulations (out of 10,000; Methods) in which the full bandit model is selected at the group level over each sub-model, when data is generated either from the corresponding sub-model (blue asterisks) or from the full model (red diamonds) for different model-comparison methods. Note that for BGLRT and GLRT, selection probabilities corresponds to type I errors (when a sub-model generated data) or to power (when the full model generated data). For AIC and BIC, selection probabilities corresponds to a lower sum of the corresponding information criterion across participants. The sub-model label refers to the component that was ablated from the full model. MB: pure MF contributions to bandit choices sub-model; MF: pure MB contributions to bandit choices sub-model; B: no constant Bias towards bandits sub-model; P: no bandit related Perseveration sub-model. B) Same as (A) but for the reward-setting models. SF: No Self Reflective MB planning sub-model; P: no Perseveration towards reward setting sub-model; LE: no Lazy MB Encoding sub-model; MF: no MF contributions towards reward-settings; BT: Bandit Traces do not affect reward settings sub-model; B: constant Biases towards bandit do not affect reward settings sub-model. (TIF) [file pcbi.1008552.s005.tif]

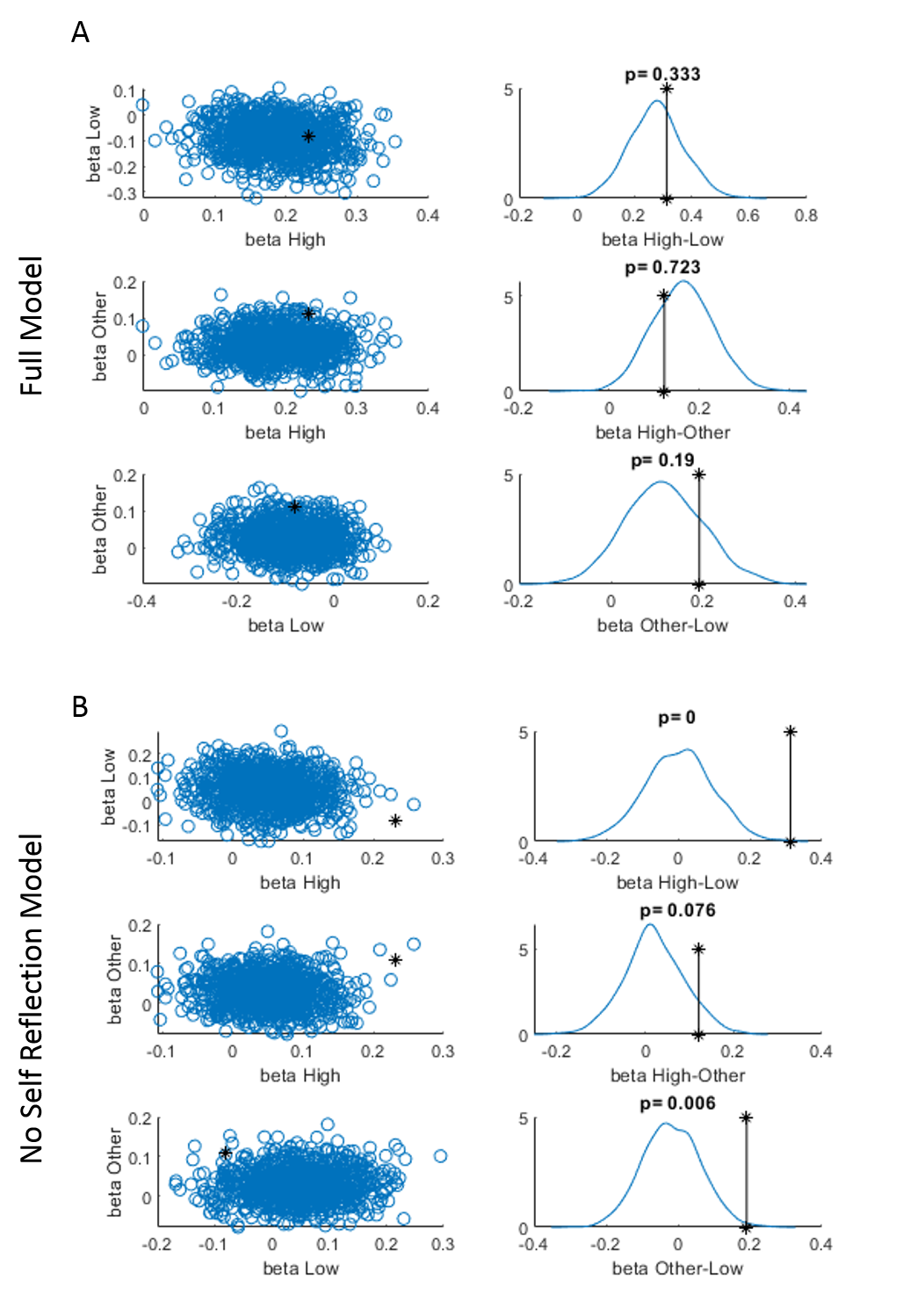

Supplement: S6 Fig — In the main text figures (Fig 5C and 5D) we generated model predictions based on 15 synthetic data sets per subject. While this methods allows noise reduction in calculations of model predations it renders a comparison between empirical and simulated effects difficult because it does not allow an assessment of how likely empirical effects will be generated by the models. Thus, we examined in greater detail the distribution of model predictions when a single dataset is generated per participant. A) We simulated for each participant a single synthetic experimental session (novel trial sequences which were generated as in the actual experiment), based on his or her best fitting parameters from the full model. We then repeated the mixed effects model reported in the section “The Effects of the Preceding Bandit-Phase on Chosen Reward Settings” for these synthetic data (i.e., for 30 synthetic participants). Thus, we obtained synthetic fixed effects for High/Low/Unrelated trials (as in Fig 5B). This entire procedure was repeated 1,001 times. Left panels present scatter plots for each pair of fixed effects (blue circles) and the empirical fixed effects (black *). Right panels present distributions for corresponding synthetic effect-contrasts (blue; estimated using Matlab’s routine “ksdensity”) and the empirical contrast (black). The titles reports the proportion of simulations which generated a contrast that is at least as large as the empirical contrast. The full model tends to generate positive contrasts and the empirical results fall comfortably within the range of model-simulations. B) Same but for the “No self-reflection sub-model”. This model predicts contrasts that are distributed around 0, and the empirical contrasts (especially High vs. Low, Other vs. Low) are very unlikely to emerge from this sub-model. (TIF) [file pcbi.1008552.s006.tif]
