# Supplementary material for: Model based planners reflect on their model-free propensities
Source: PLoS Comput Biol. 2021 Jan 7;17(1):e1008552. doi: 10.1371/journal.pcbi.1008552 (PMC7817042; doi:10.1371/journal.pcbi.1008552)
Supplement: S1 Table — The table shows the 25, 50 and 75 percentiles across participants. (See ‘Computational Models’ in methods for a full description of the model and its parameters). (PDF) [file pcbi.1008552.s007.pdf]

Parameter	β_{MB}	β_{MF}	lr	f	τ	β_C	b_2	b_3	b_4
25%	1.54	0.70	0.02	0.17	0.02	-9.24	-0.45	-0.58	-0.42
50%	3.18	4.01	0.05	0.22	0.13	-1.62	-0.05	-0.03	0.05
75%	4.60	10.00	0.22	0.40	0.31	0.84	0.42	0.14	0.33

Parameter	γ_{C}	$\gamma_{lazy\ enc}$	$\gamma_{\mathrm{self\ ref}}$	λ	$\gamma_{rs,MF}$	$\gamma_{bandit\ trace}$	$\gamma_{bandit\ bias}$
25%	-10.00	0.00	0.00	0.28	0.00	-6.24	0.45
50%	-3.34	0.21	0.95	0.50	0.39	-1.19	1.06
75%	-0.25	0.41	4.83	0.64	1.58	1.03	1.55
