# Supplementary material for: Model based planners reflect on their model-free propensities
Source: PLoS Comput Biol. 2021 Jan 7;17(1):e1008552. doi: 10.1371/journal.pcbi.1008552 (PMC7817042; doi:10.1371/journal.pcbi.1008552)
Supplement: S1 Presentation — (PPTX) [file pcbi.1008552.s008.pptx]

## Slide 1
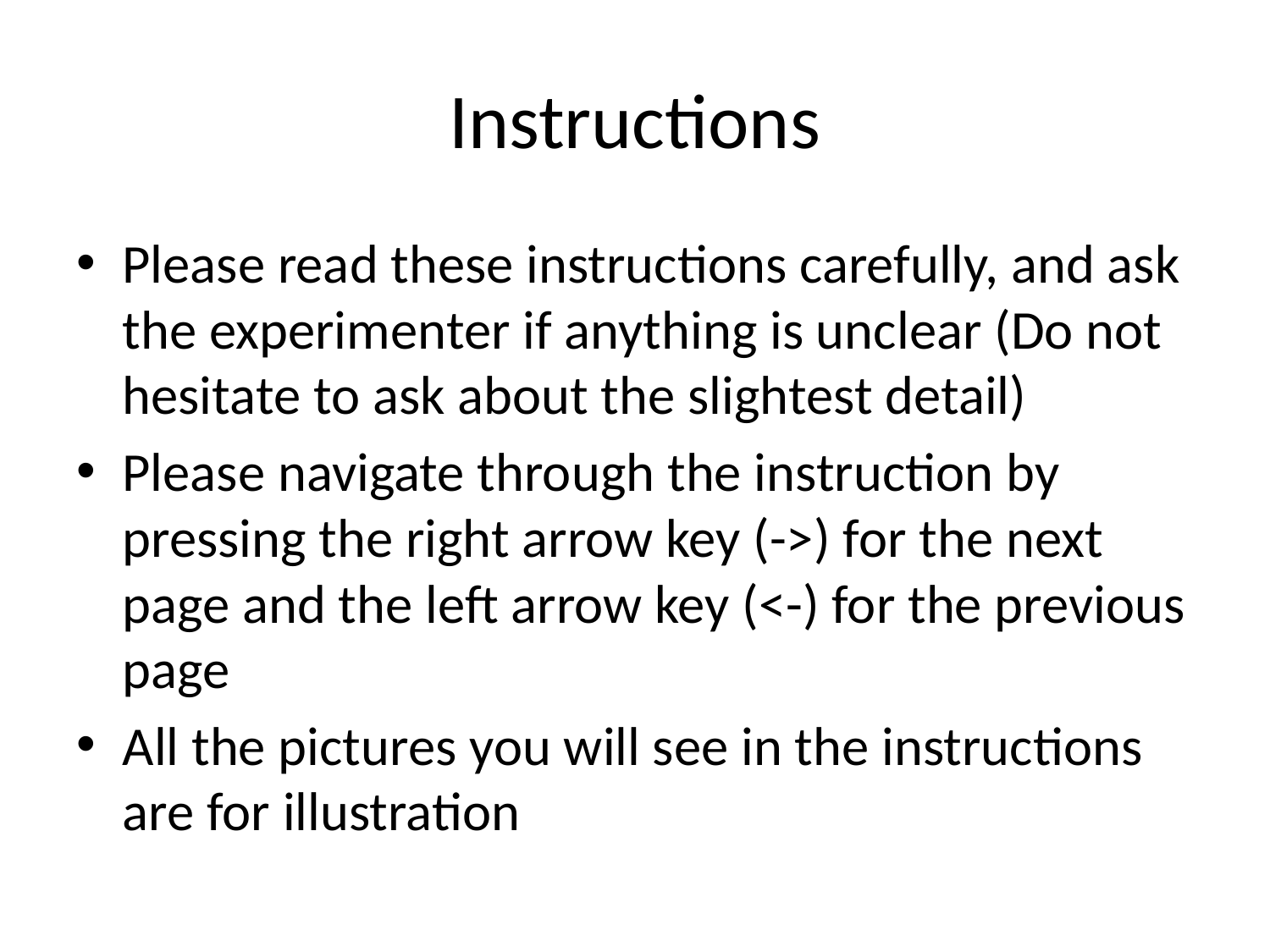

# Instructions
Please read these instructions carefully, and ask the experimenter if anything is unclear (Do not hesitate to ask about the slightest detail)
Please navigate through the instruction by pressing the right arrow key (->) for the next page and the left arrow key (<-) for the previous page
All the pictures you will see in the instructions are for illustration

## Slide 2
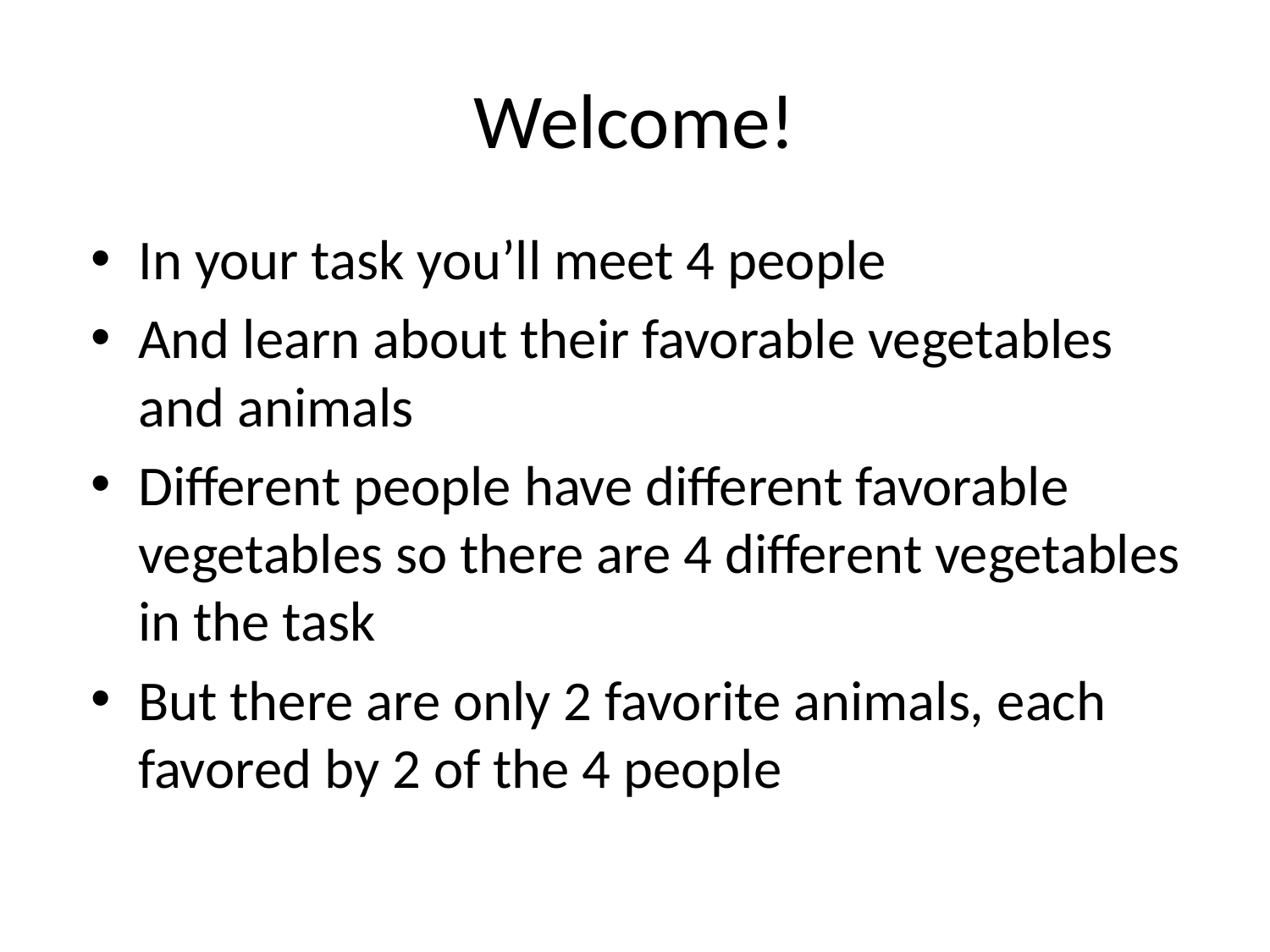

# Welcome!
In your task you’ll meet 4 people
And learn about their favorable vegetables and animals
Different people have different favorable vegetables so there are 4 different vegetables in the task
But there are only 2 favorite animals, each favored by 2 of the 4 people

## Slide 3
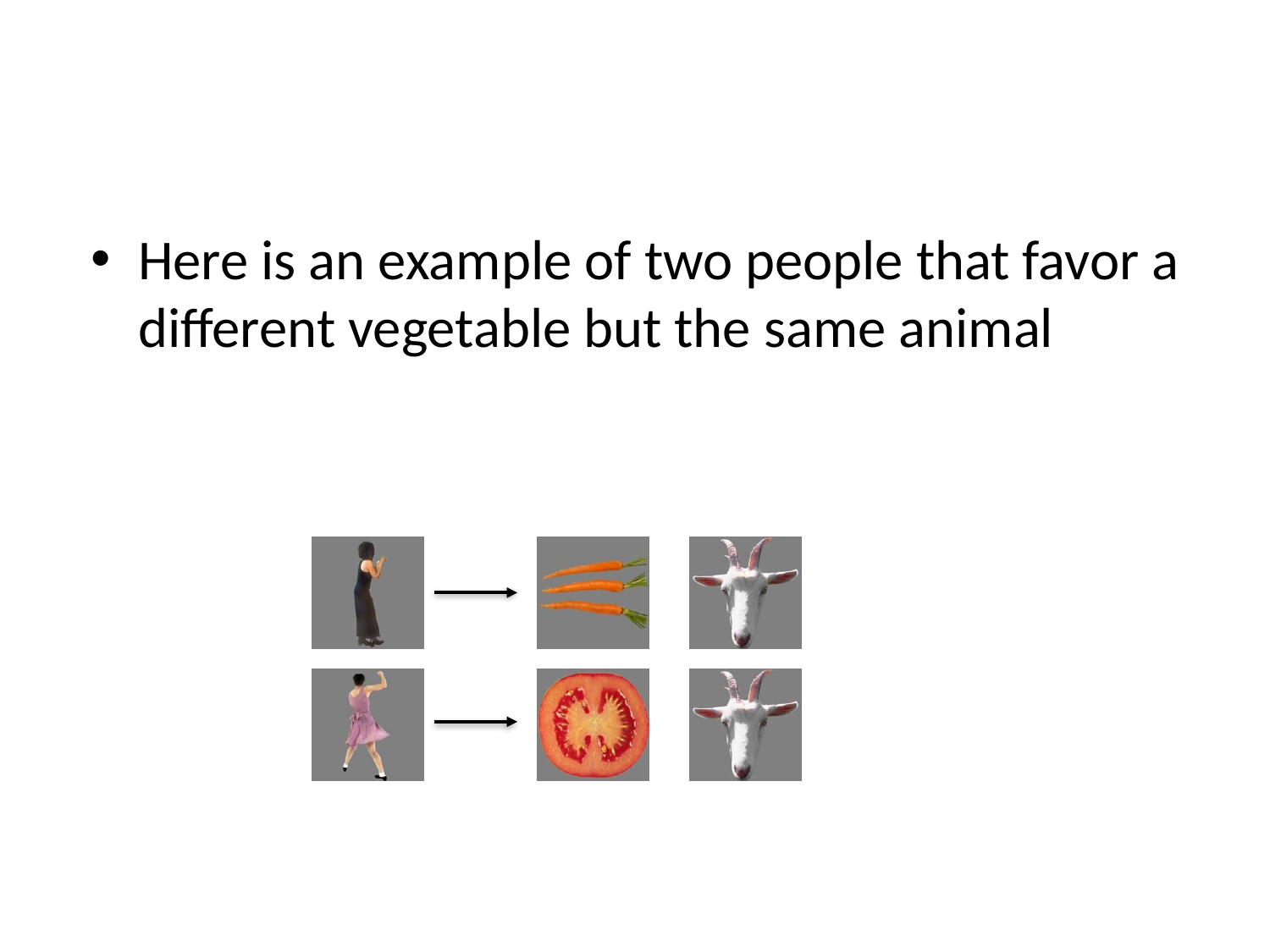

Here is an example of two people that favor a different vegetable but the same animal

## Slide 4
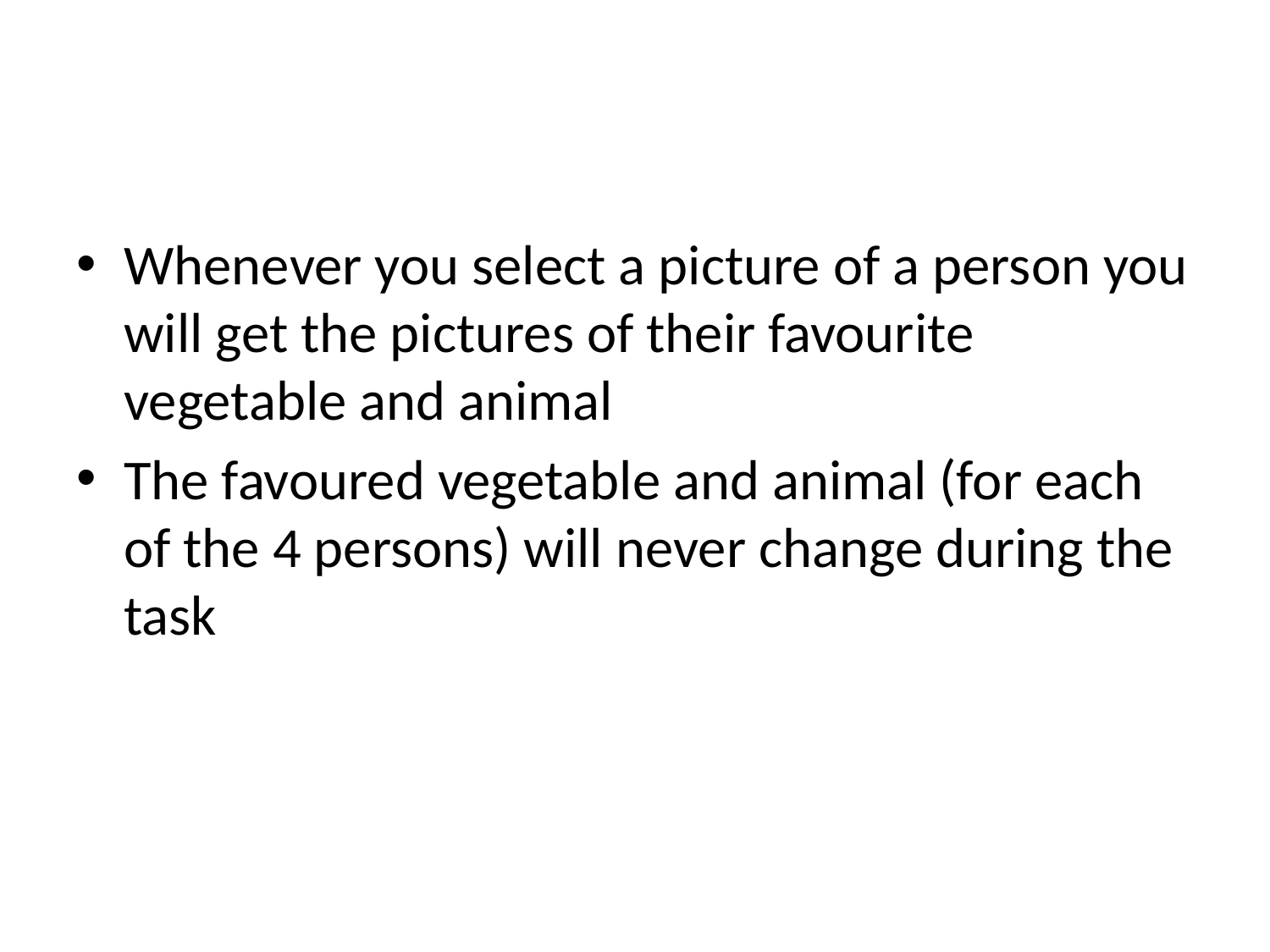

Whenever you select a picture of a person you will get the pictures of their favourite vegetable and animal
The favoured vegetable and animal (for each of the 4 persons) will never change during the task

## Slide 5
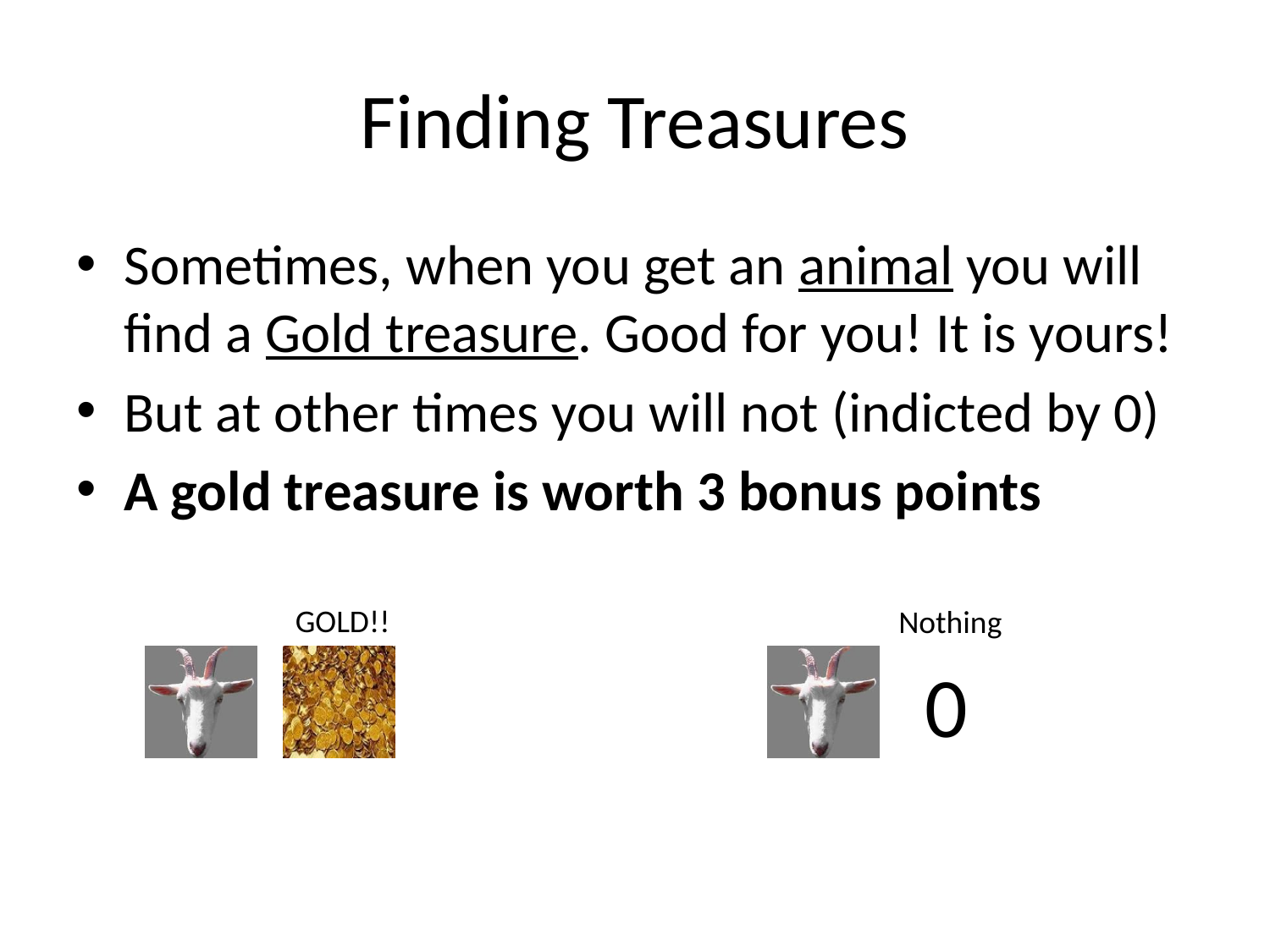

# Finding Treasures
Sometimes, when you get an animal you will find a Gold treasure. Good for you! It is yours!
But at other times you will not (indicted by 0)
A gold treasure is worth 3 bonus points
GOLD!!
Nothing
0

## Slide 6
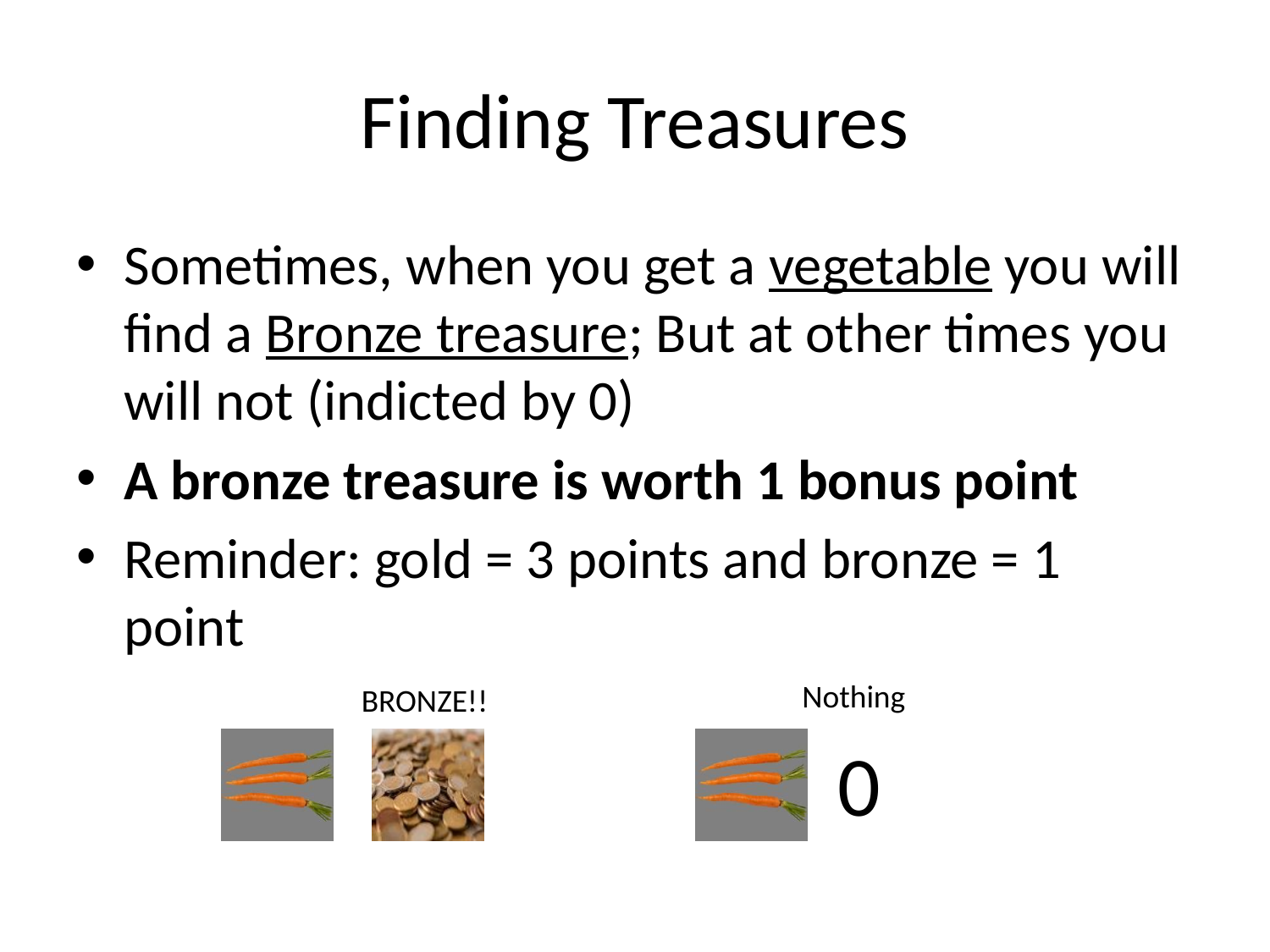

# Finding Treasures
Sometimes, when you get a vegetable you will find a Bronze treasure; But at other times you will not (indicted by 0)
A bronze treasure is worth 1 bonus point
Reminder: gold = 3 points and bronze = 1 point
Nothing
BRONZE!!
0

## Slide 7
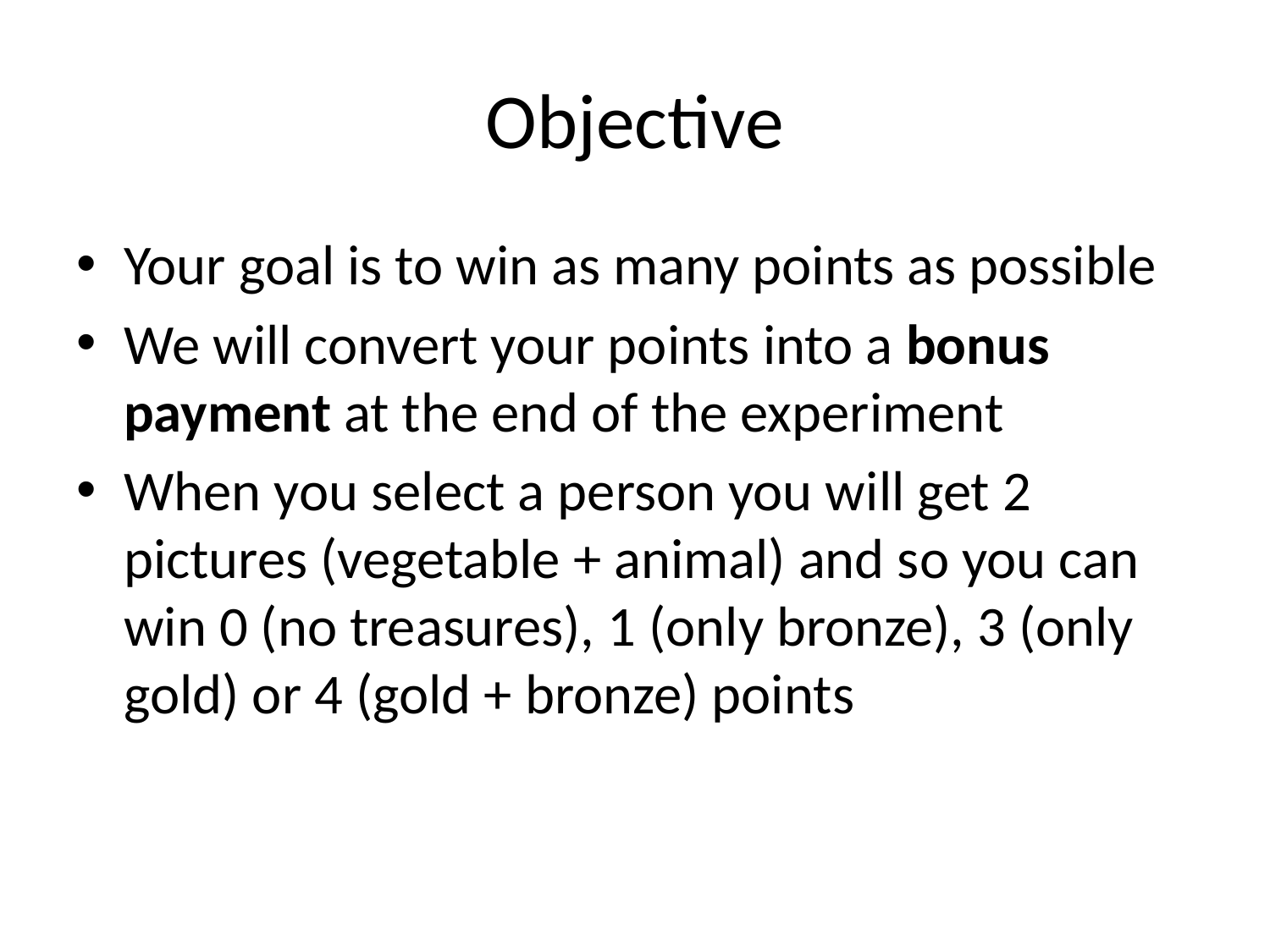

# Objective
Your goal is to win as many points as possible
We will convert your points into a bonus payment at the end of the experiment
When you select a person you will get 2 pictures (vegetable + animal) and so you can win 0 (no treasures), 1 (only bronze), 3 (only gold) or 4 (gold + bronze) points

## Slide 8
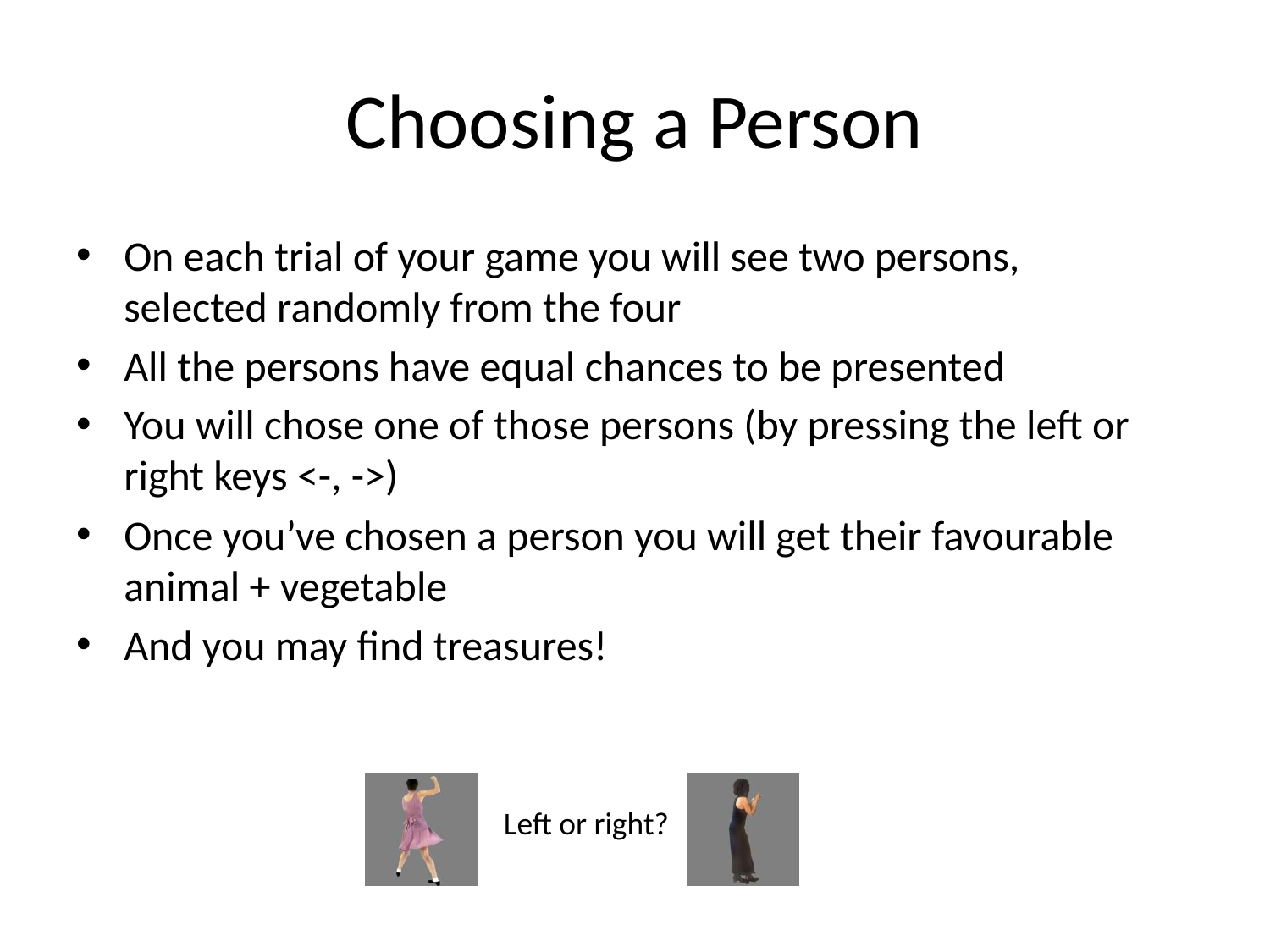

# Choosing a Person
On each trial of your game you will see two persons, selected randomly from the four
All the persons have equal chances to be presented
You will chose one of those persons (by pressing the left or right keys <-, ->)
Once you’ve chosen a person you will get their favourable animal + vegetable
And you may find treasures!
Left or right?

## Slide 9
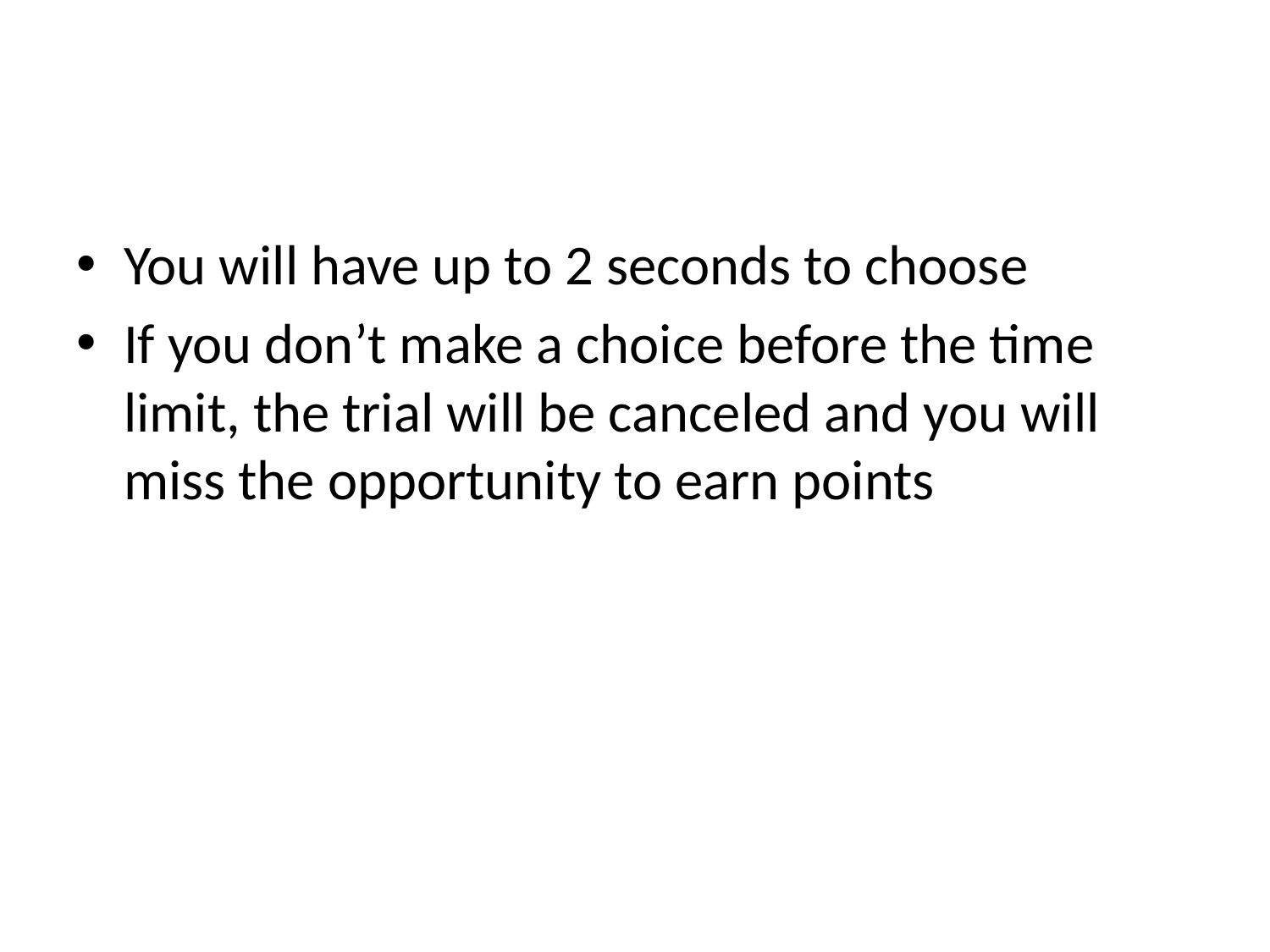

You will have up to 2 seconds to choose
If you don’t make a choice before the time limit, the trial will be canceled and you will miss the opportunity to earn points

## Slide 10
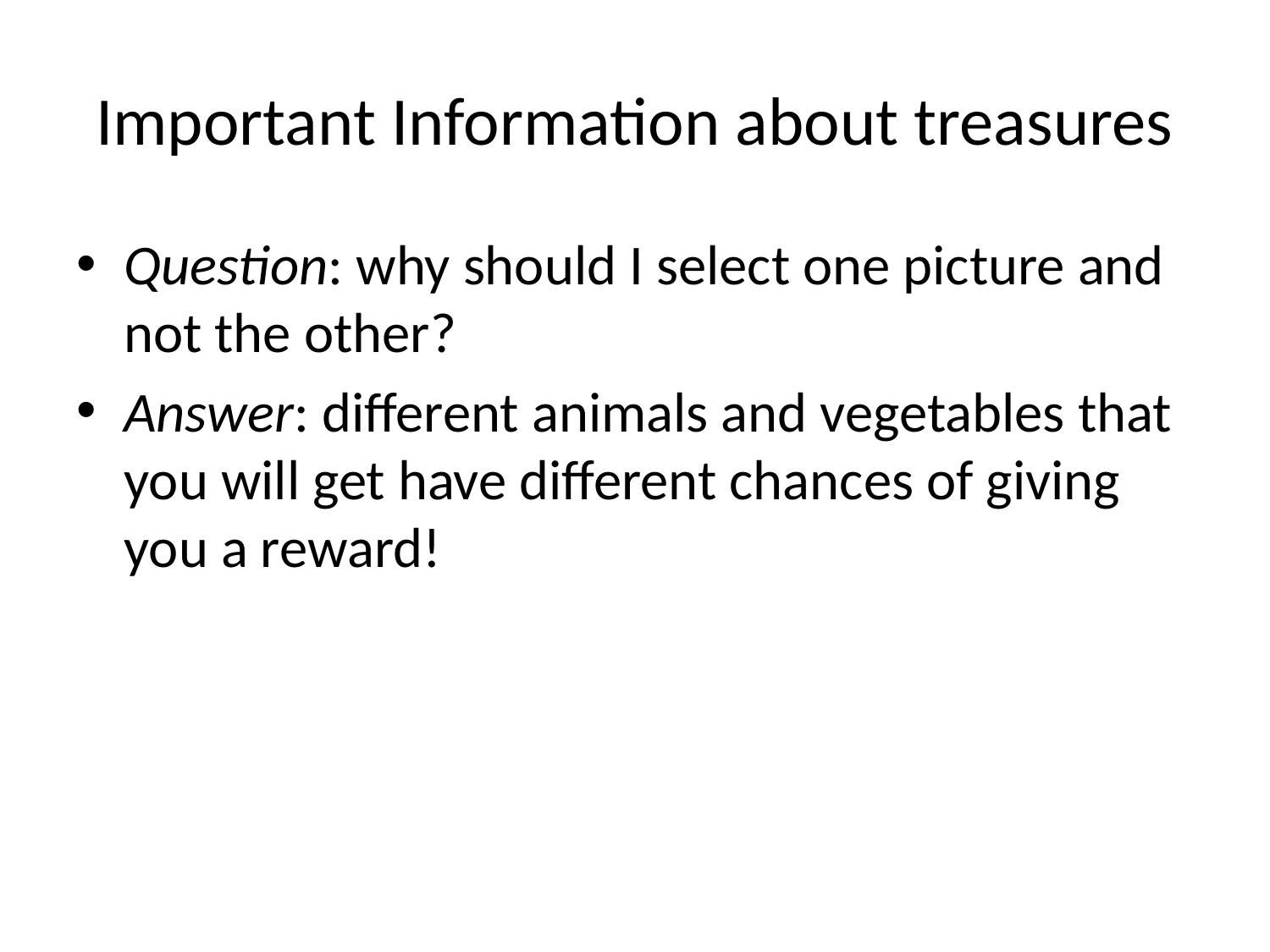

# Important Information about treasures
Question: why should I select one picture and not the other?
Answer: different animals and vegetables that you will get have different chances of giving you a reward!

## Slide 11
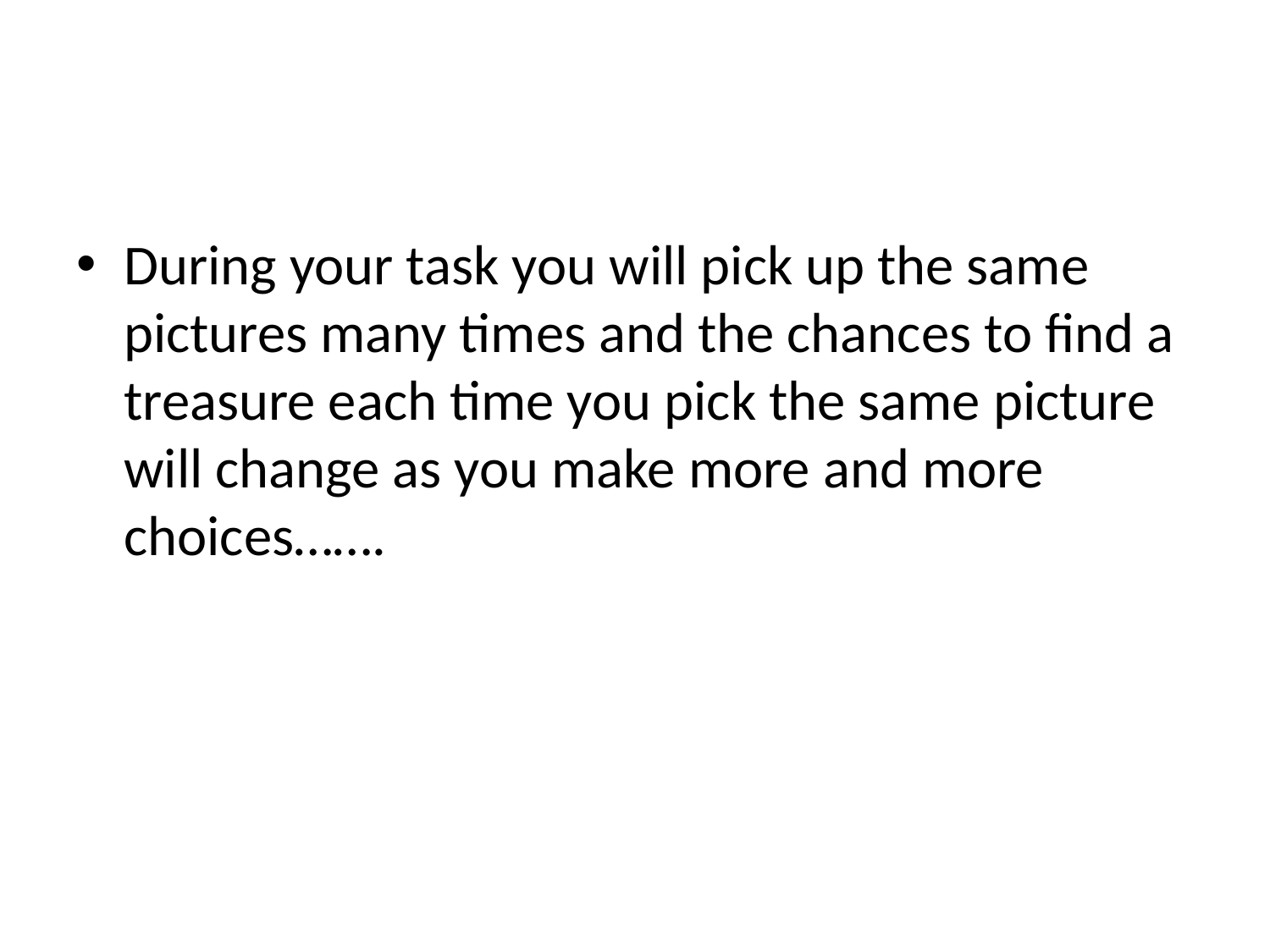

During your task you will pick up the same pictures many times and the chances to find a treasure each time you pick the same picture will change as you make more and more choices…….

## Slide 12
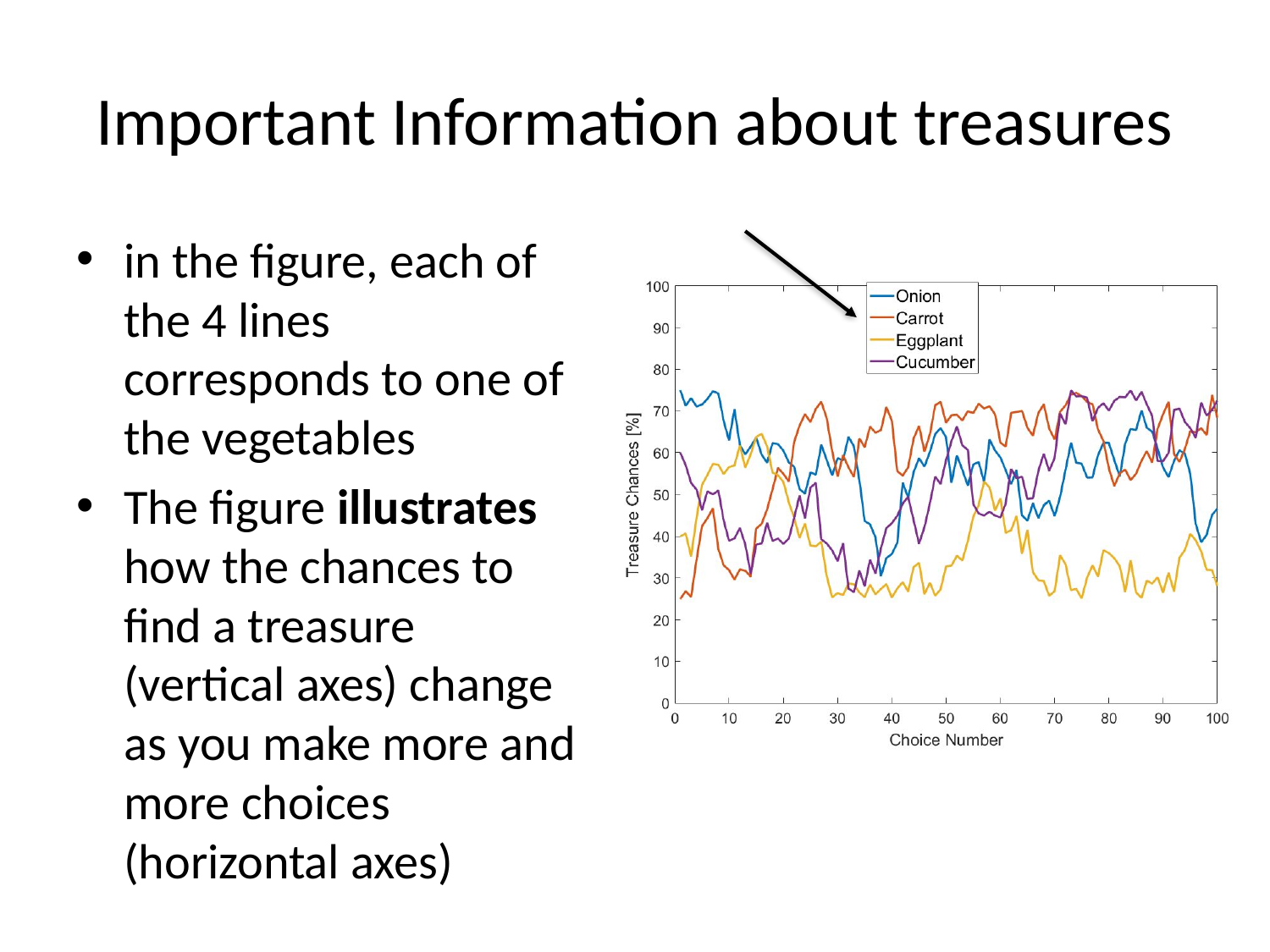

# Important Information about treasures
in the figure, each of the 4 lines corresponds to one of the vegetables
The figure illustrates how the chances to find a treasure (vertical axes) change as you make more and more choices (horizontal axes)

## Slide 13
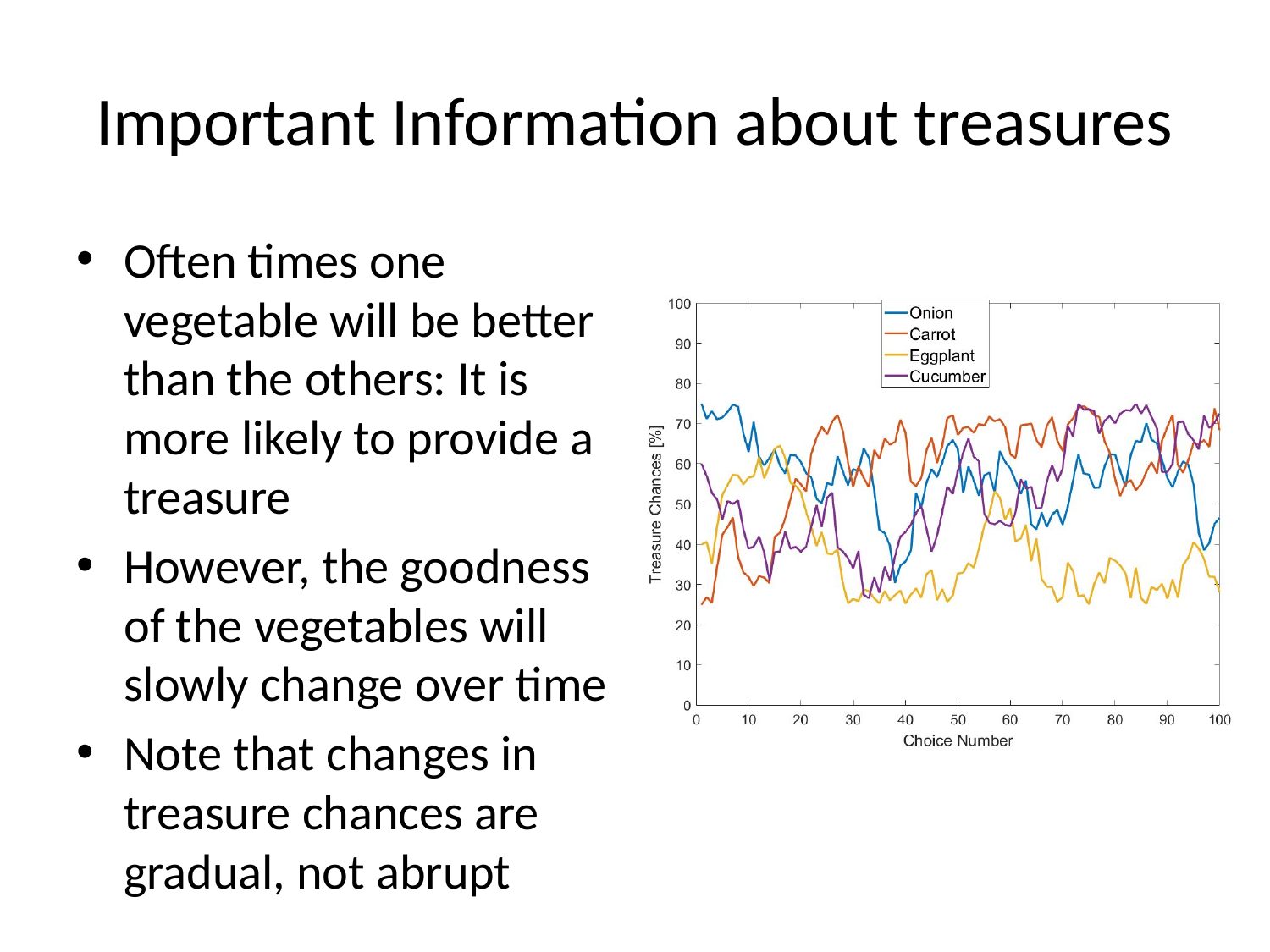

# Important Information about treasures
Often times one vegetable will be better than the others: It is more likely to provide a treasure
However, the goodness of the vegetables will slowly change over time
Note that changes in treasure chances are gradual, not abrupt

## Slide 14
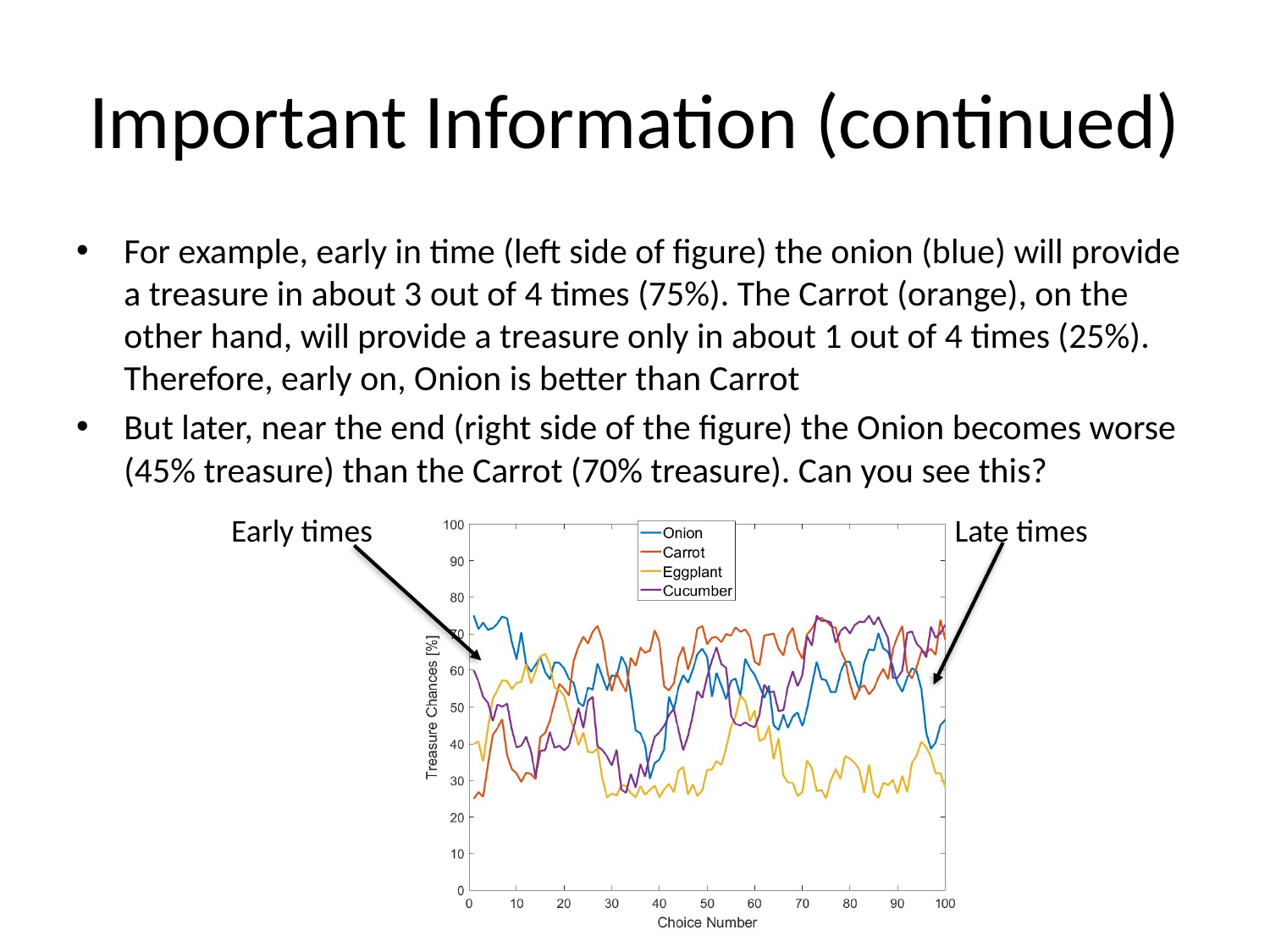

# Important Information (continued)
For example, early in time (left side of figure) the onion (blue) will provide a treasure in about 3 out of 4 times (75%). The Carrot (orange), on the other hand, will provide a treasure only in about 1 out of 4 times (25%). Therefore, early on, Onion is better than Carrot
But later, near the end (right side of the figure) the Onion becomes worse (45% treasure) than the Carrot (70% treasure). Can you see this?
Early times
Late times

## Slide 15
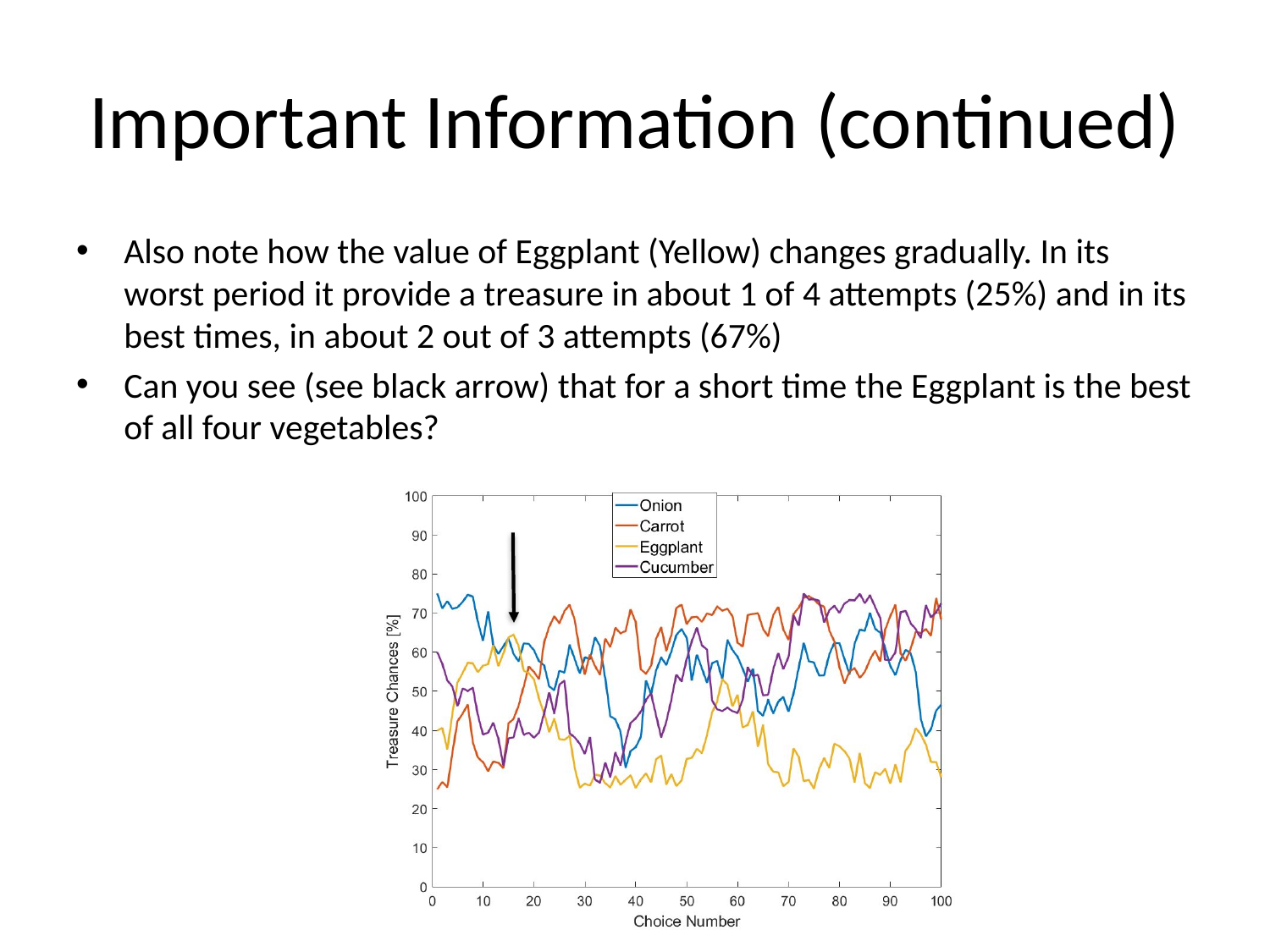

# Important Information (continued)
Also note how the value of Eggplant (Yellow) changes gradually. In its worst period it provide a treasure in about 1 of 4 attempts (25%) and in its best times, in about 2 out of 3 attempts (67%)
Can you see (see black arrow) that for a short time the Eggplant is the best of all four vegetables?

## Slide 16
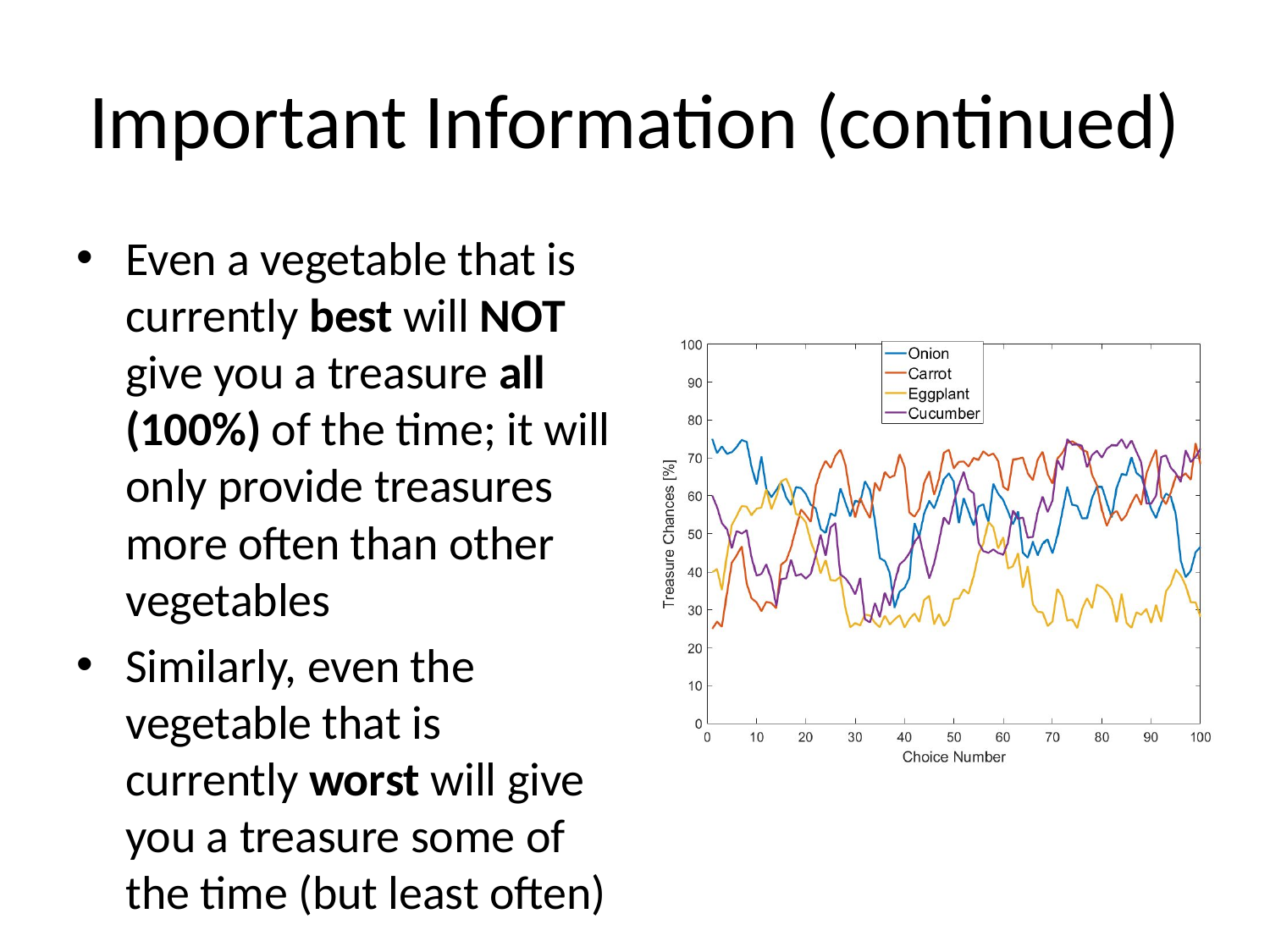

# Important Information (continued)
Even a vegetable that is currently best will NOT give you a treasure all (100%) of the time; it will only provide treasures more often than other vegetables
Similarly, even the vegetable that is currently worst will give you a treasure some of the time (but least often)

## Slide 17
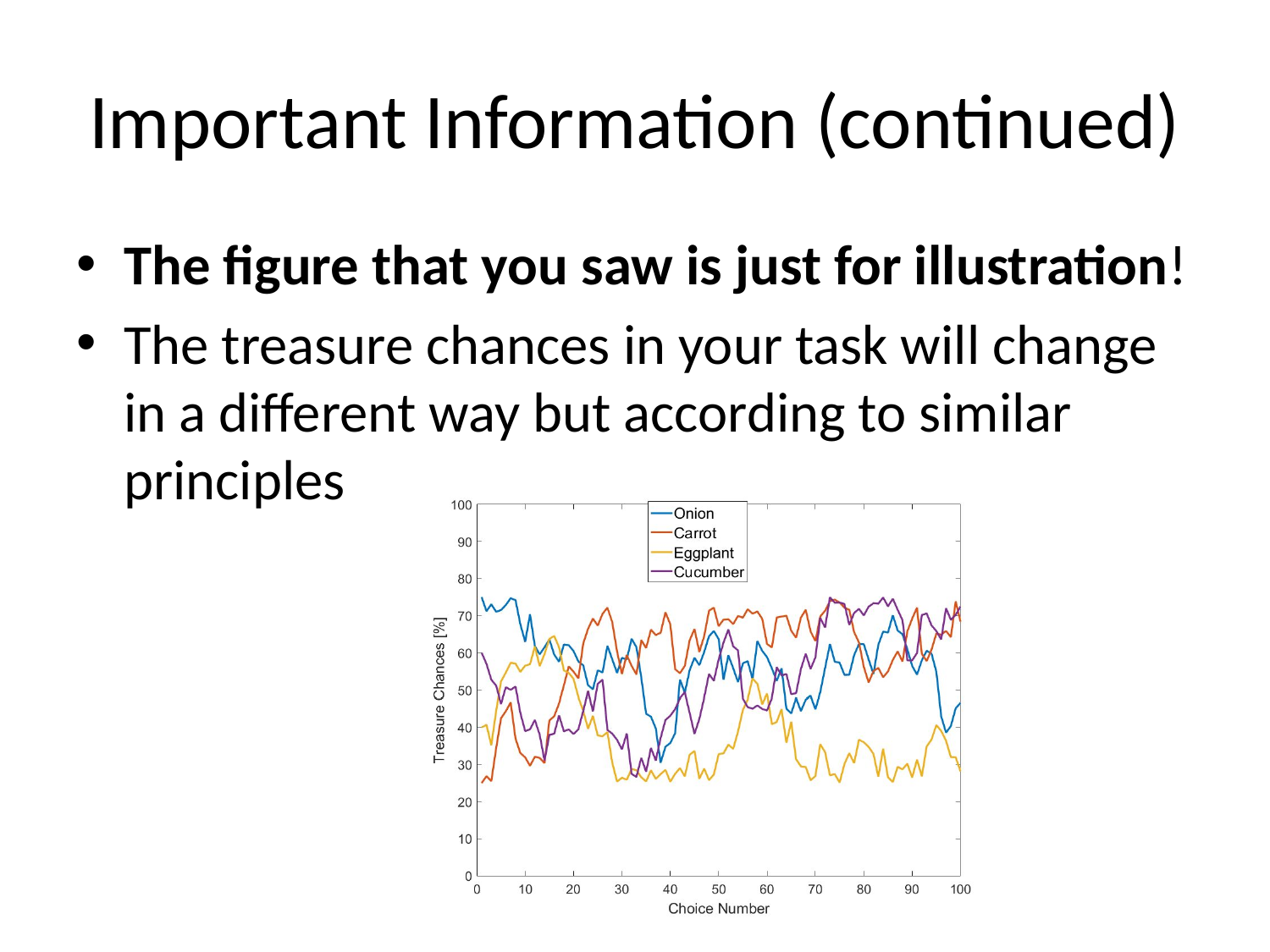

# Important Information (continued)
The figure that you saw is just for illustration!
The treasure chances in your task will change in a different way but according to similar principles

## Slide 18
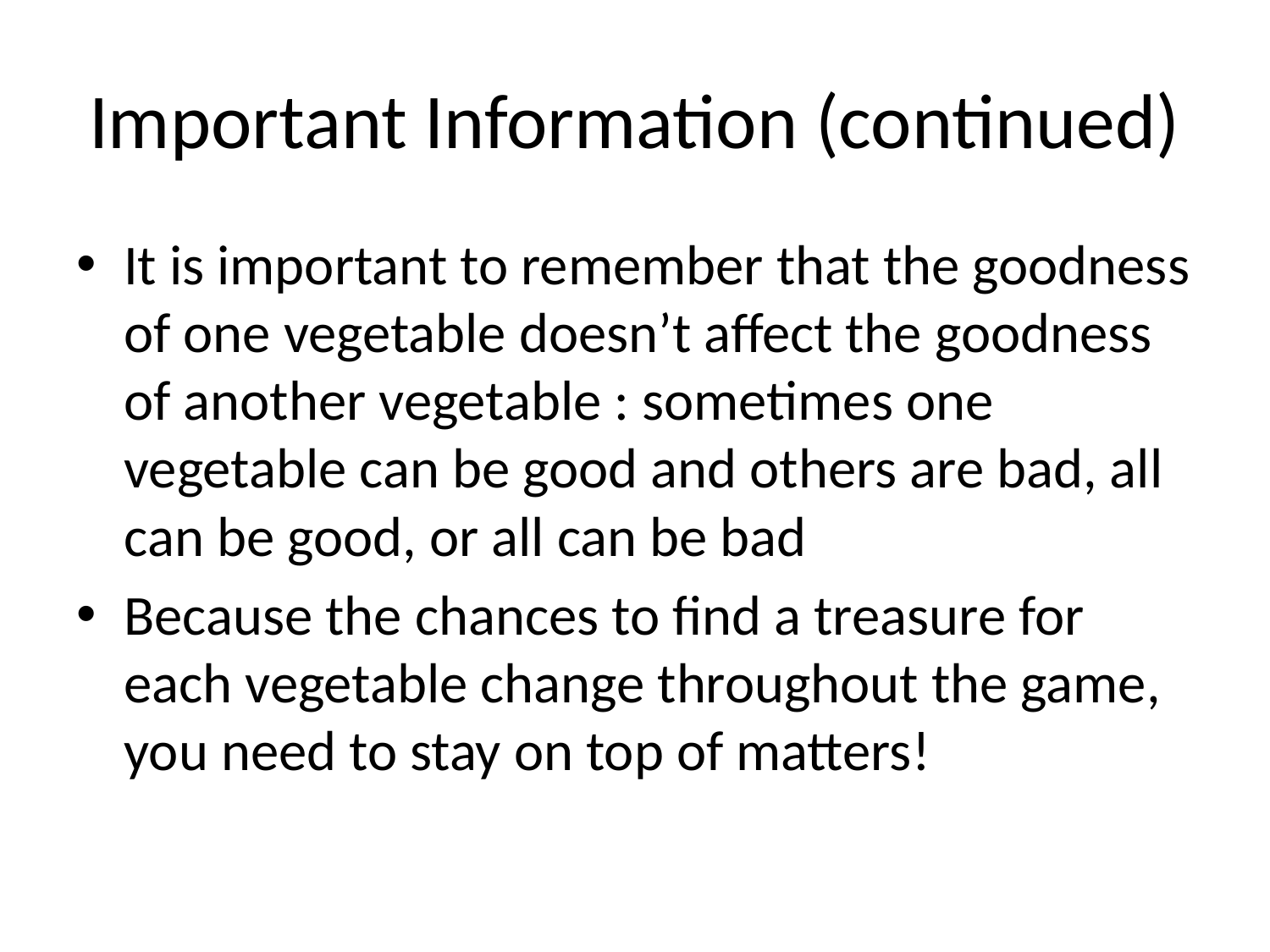

# Important Information (continued)
It is important to remember that the goodness of one vegetable doesn’t affect the goodness of another vegetable : sometimes one vegetable can be good and others are bad, all can be good, or all can be bad
Because the chances to find a treasure for each vegetable change throughout the game, you need to stay on top of matters!

## Slide 19
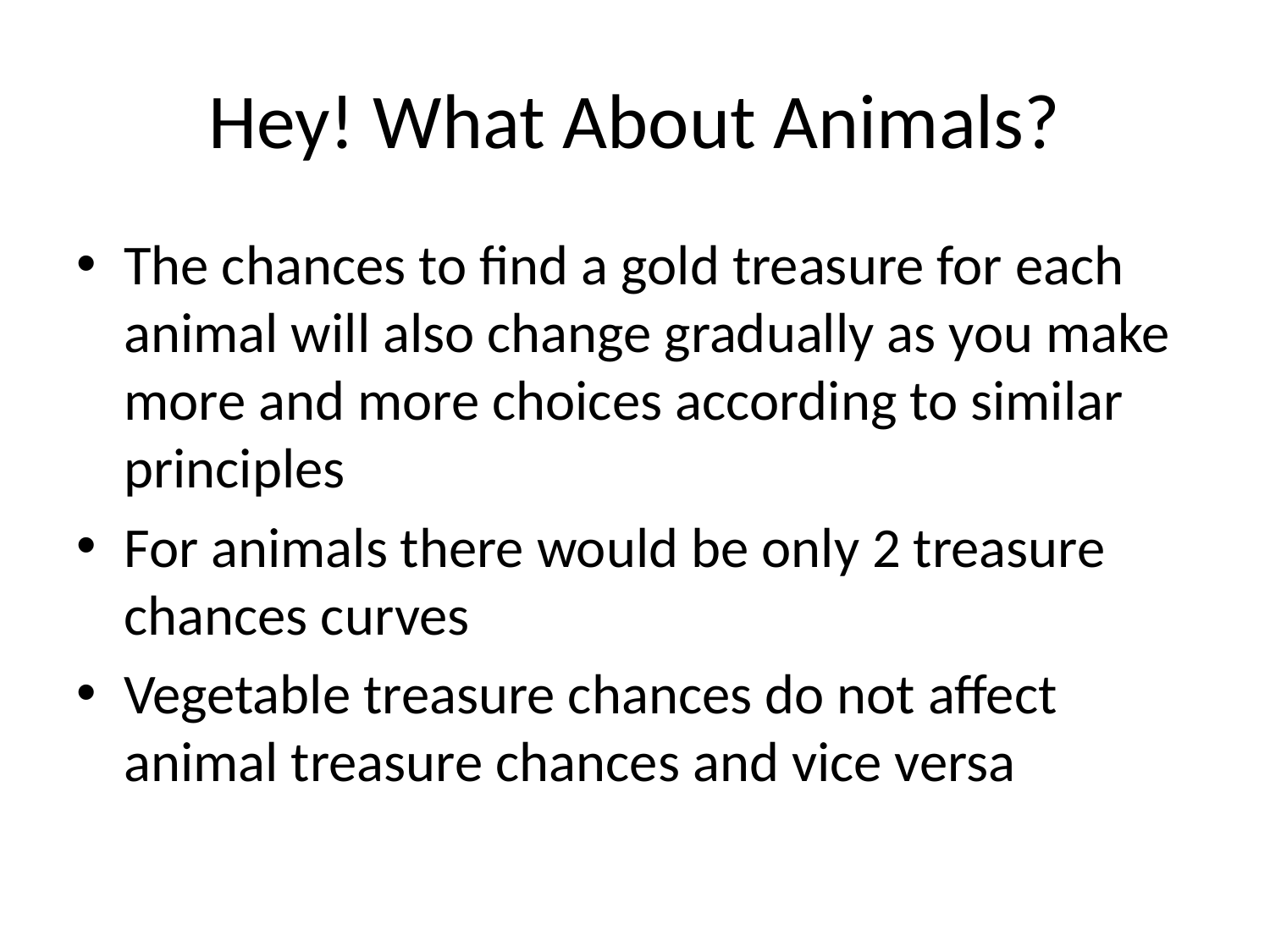

# Hey! What About Animals?
The chances to find a gold treasure for each animal will also change gradually as you make more and more choices according to similar principles
For animals there would be only 2 treasure chances curves
Vegetable treasure chances do not affect animal treasure chances and vice versa

## Slide 20
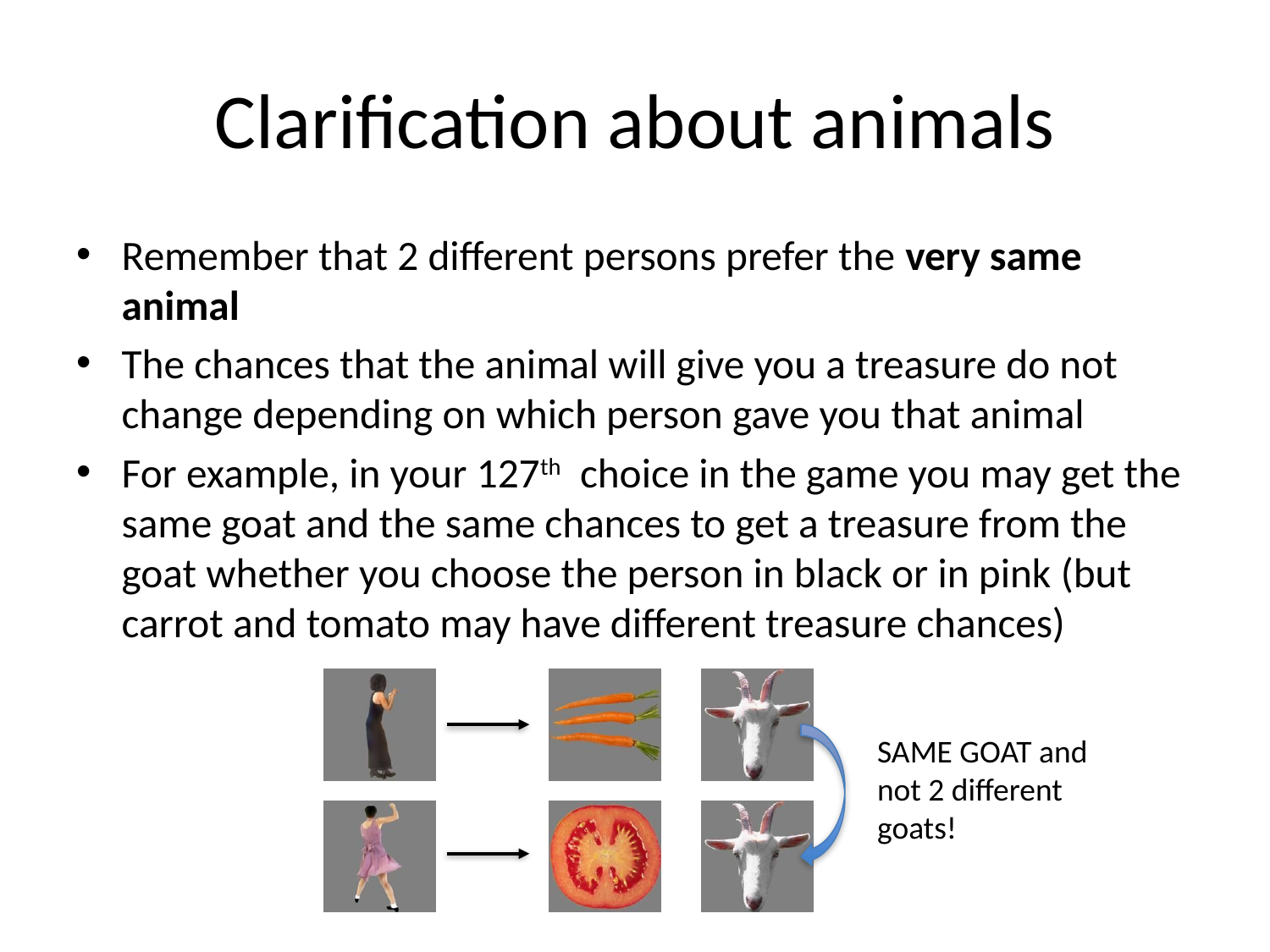

# Clarification about animals
Remember that 2 different persons prefer the very same animal
The chances that the animal will give you a treasure do not change depending on which person gave you that animal
For example, in your 127th choice in the game you may get the same goat and the same chances to get a treasure from the goat whether you choose the person in black or in pink (but carrot and tomato may have different treasure chances)
SAME GOAT and not 2 different goats!

## Slide 21
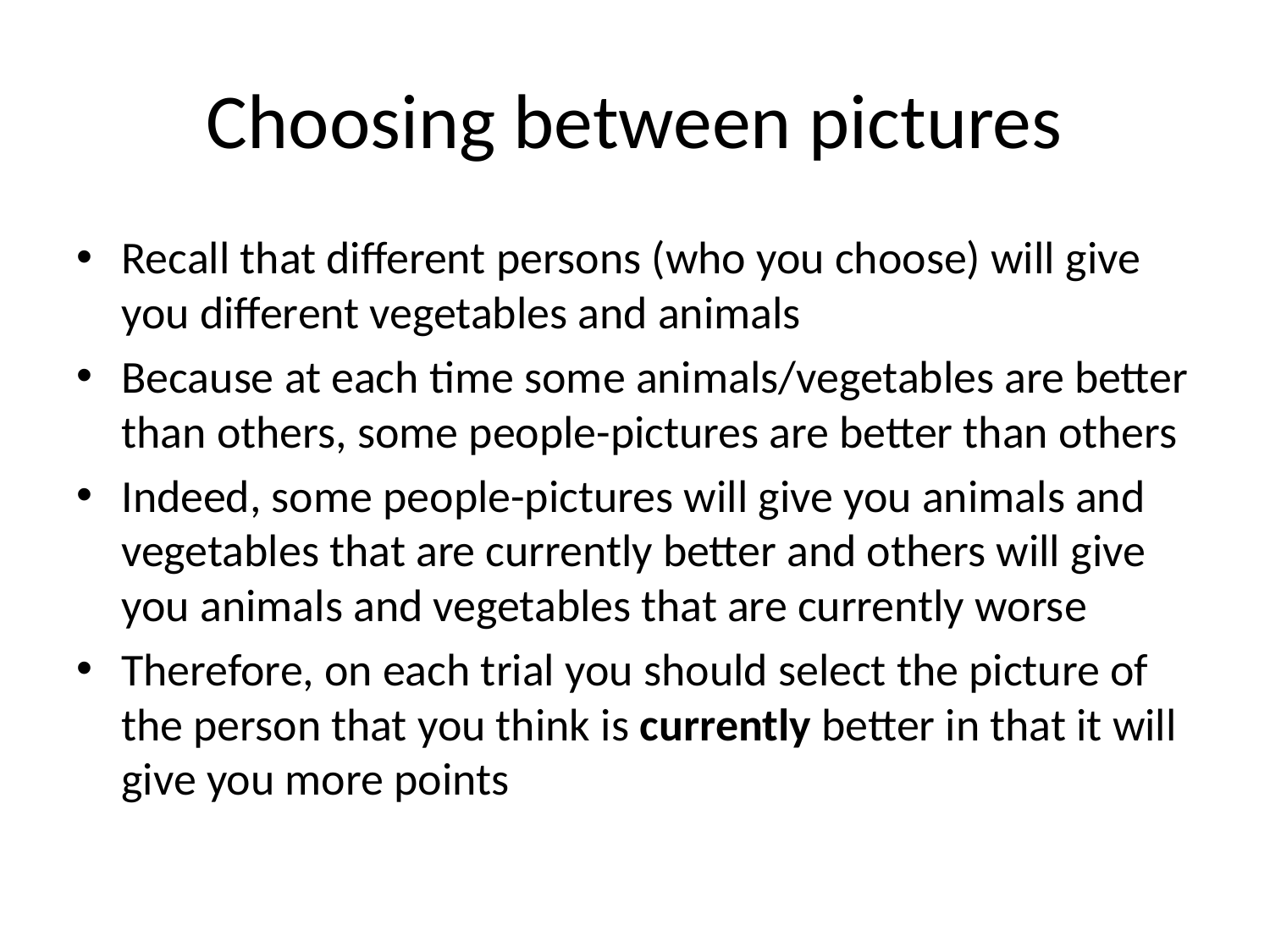

# Choosing between pictures
Recall that different persons (who you choose) will give you different vegetables and animals
Because at each time some animals/vegetables are better than others, some people-pictures are better than others
Indeed, some people-pictures will give you animals and vegetables that are currently better and others will give you animals and vegetables that are currently worse
Therefore, on each trial you should select the picture of the person that you think is currently better in that it will give you more points

## Slide 22
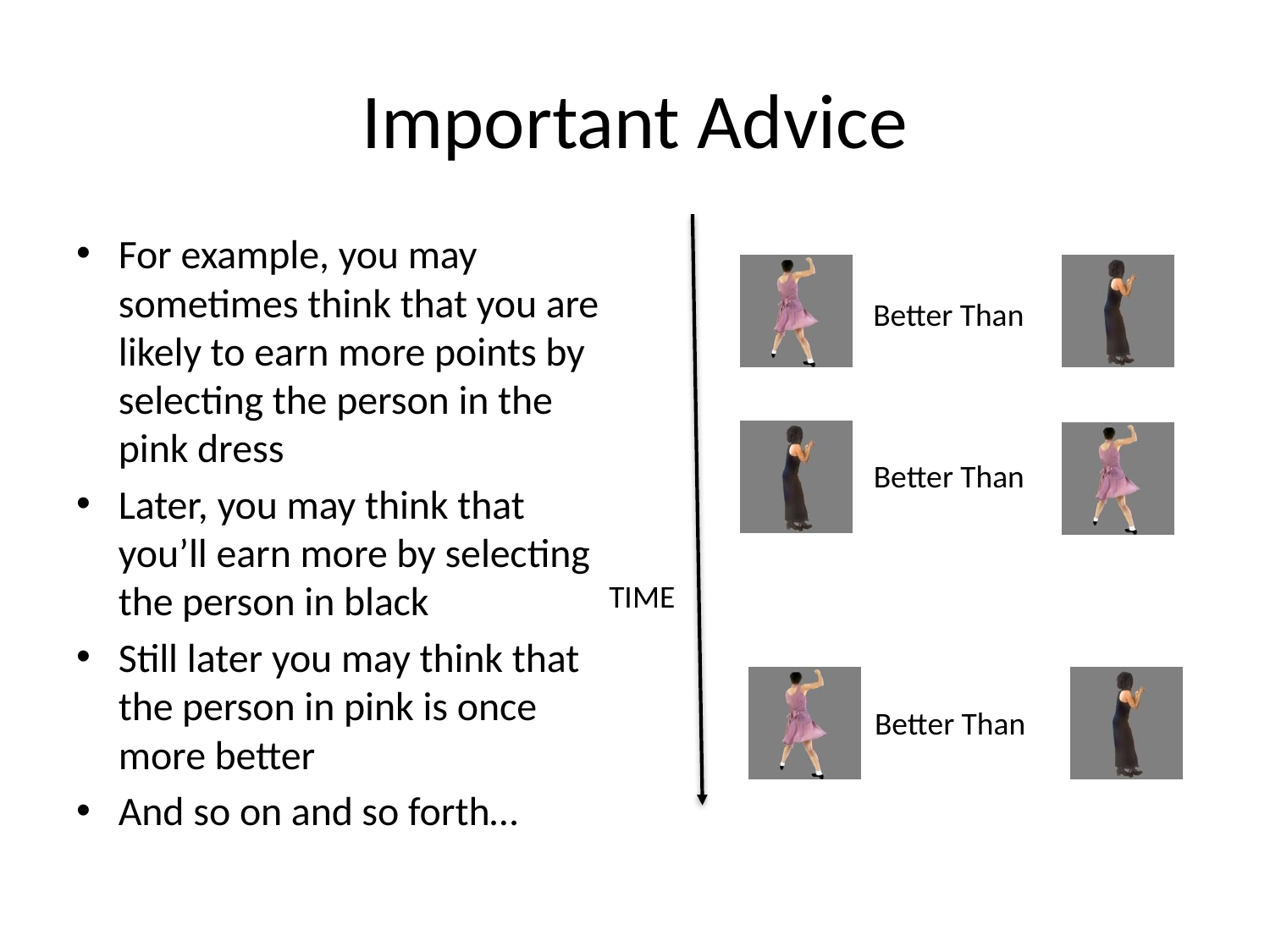

# Important Advice
For example, you may sometimes think that you are likely to earn more points by selecting the person in the pink dress
Later, you may think that you’ll earn more by selecting the person in black
Still later you may think that the person in pink is once more better
And so on and so forth…
Better Than
Better Than
TIME
Better Than

## Slide 23
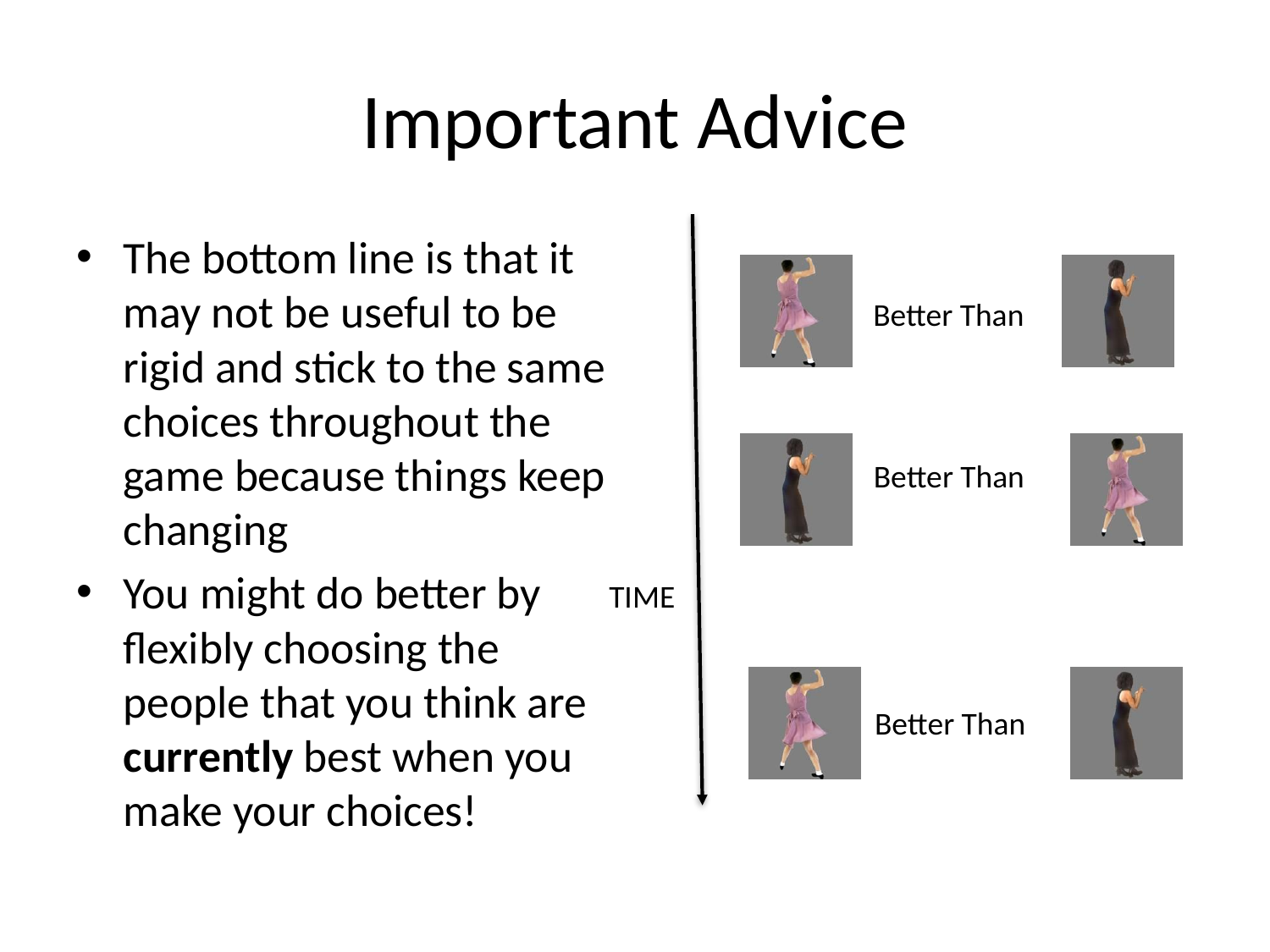

# Important Advice
The bottom line is that it may not be useful to be rigid and stick to the same choices throughout the game because things keep changing
You might do better by flexibly choosing the people that you think are currently best when you make your choices!
Better Than
Better Than
TIME
Better Than

## Slide 24
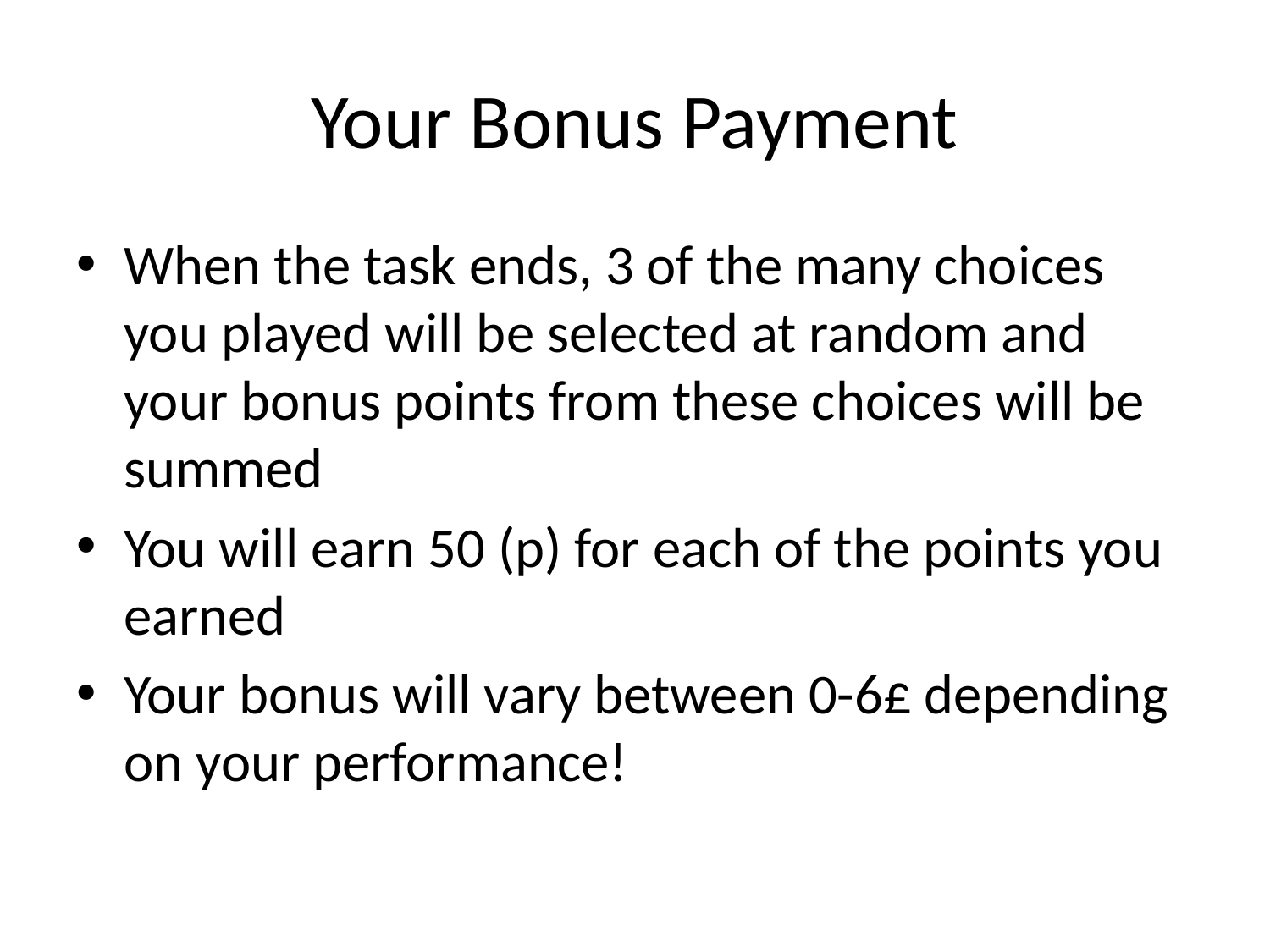

# Your Bonus Payment
When the task ends, 3 of the many choices you played will be selected at random and your bonus points from these choices will be summed
You will earn 50 (p) for each of the points you earned
Your bonus will vary between 0-6£ depending on your performance!

## Slide 25
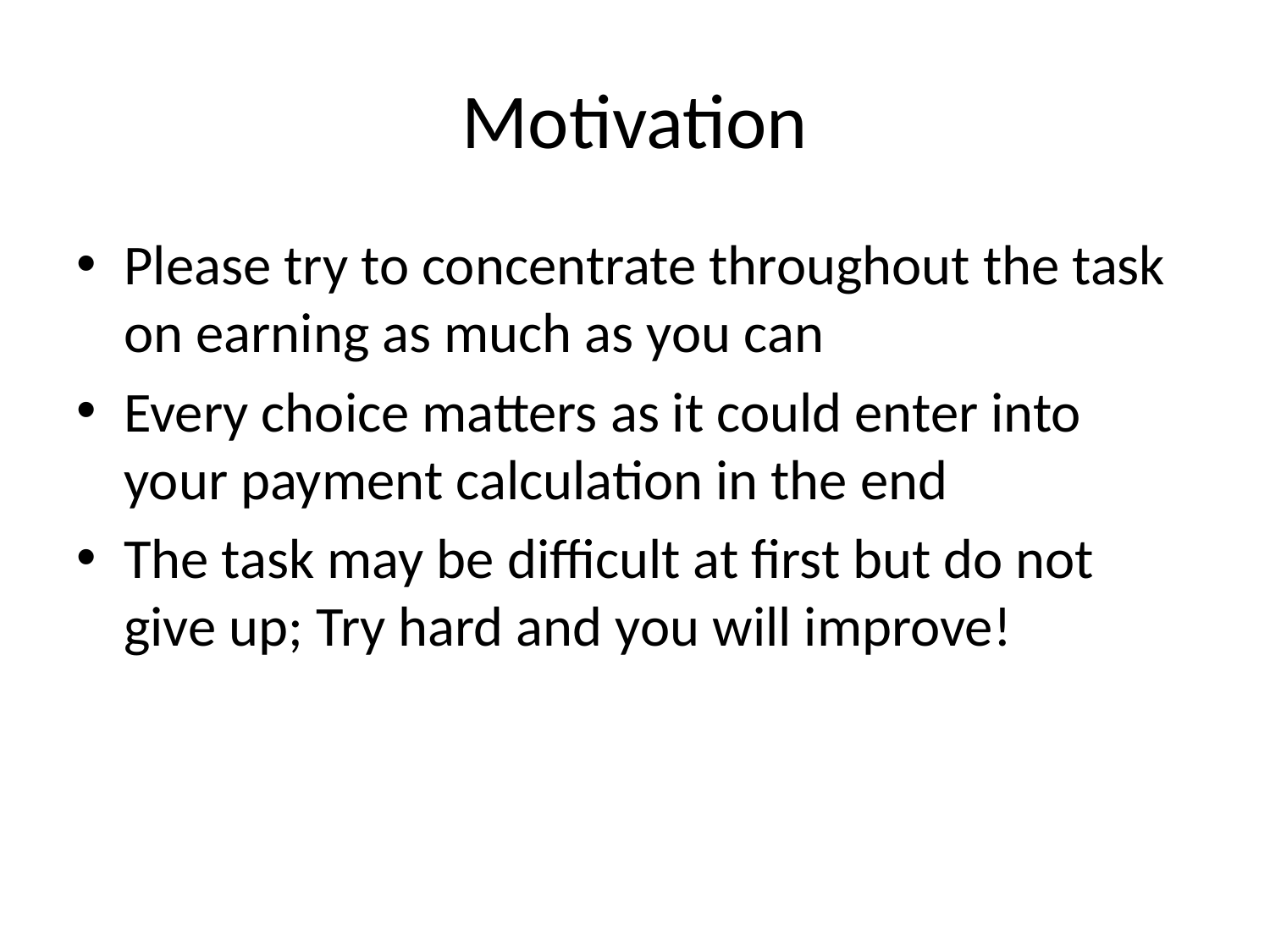

# Motivation
Please try to concentrate throughout the task on earning as much as you can
Every choice matters as it could enter into your payment calculation in the end
The task may be difficult at first but do not give up; Try hard and you will improve!

## Slide 26
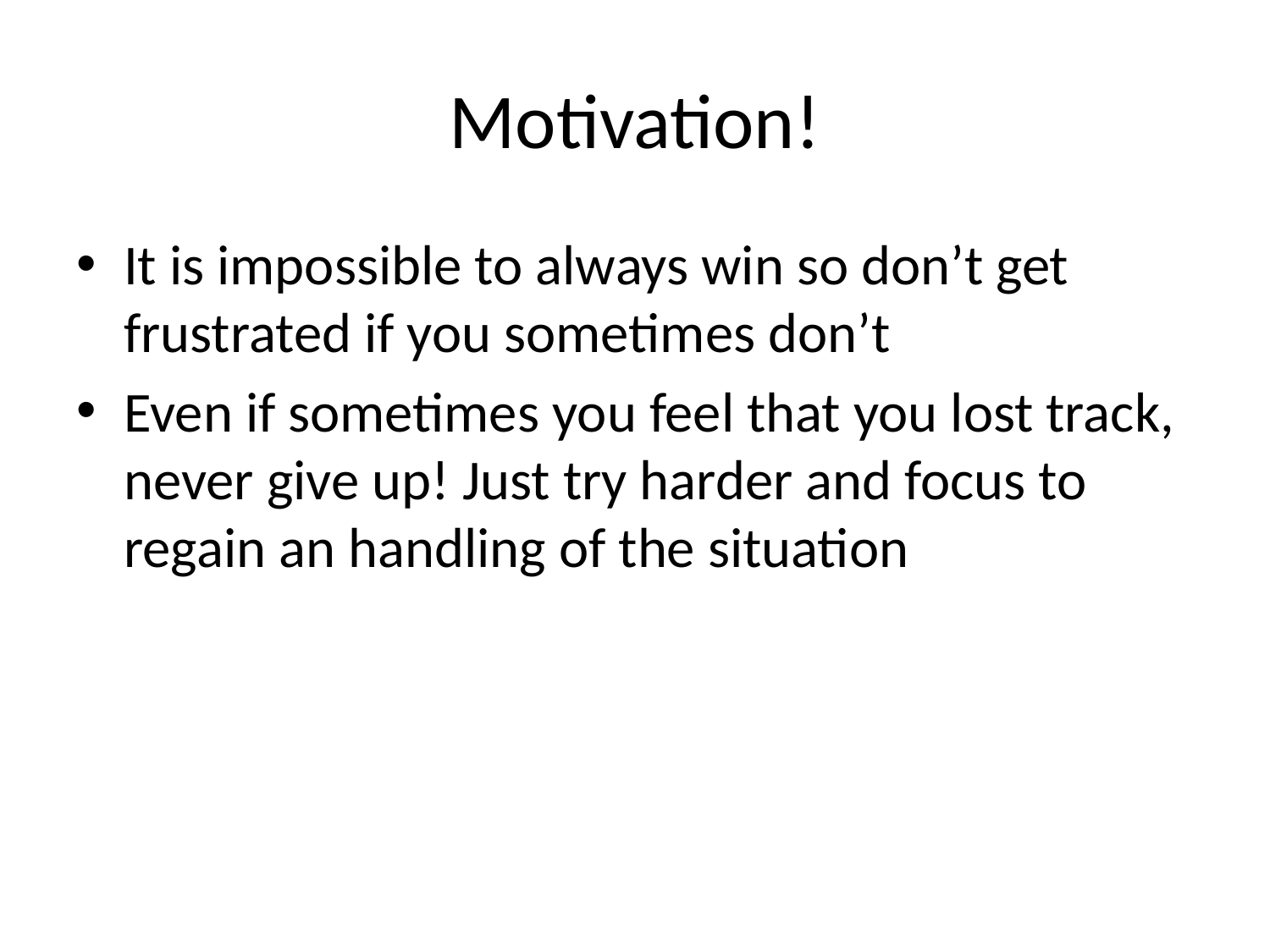

# Motivation!
It is impossible to always win so don’t get frustrated if you sometimes don’t
Even if sometimes you feel that you lost track, never give up! Just try harder and focus to regain an handling of the situation

## Slide 27
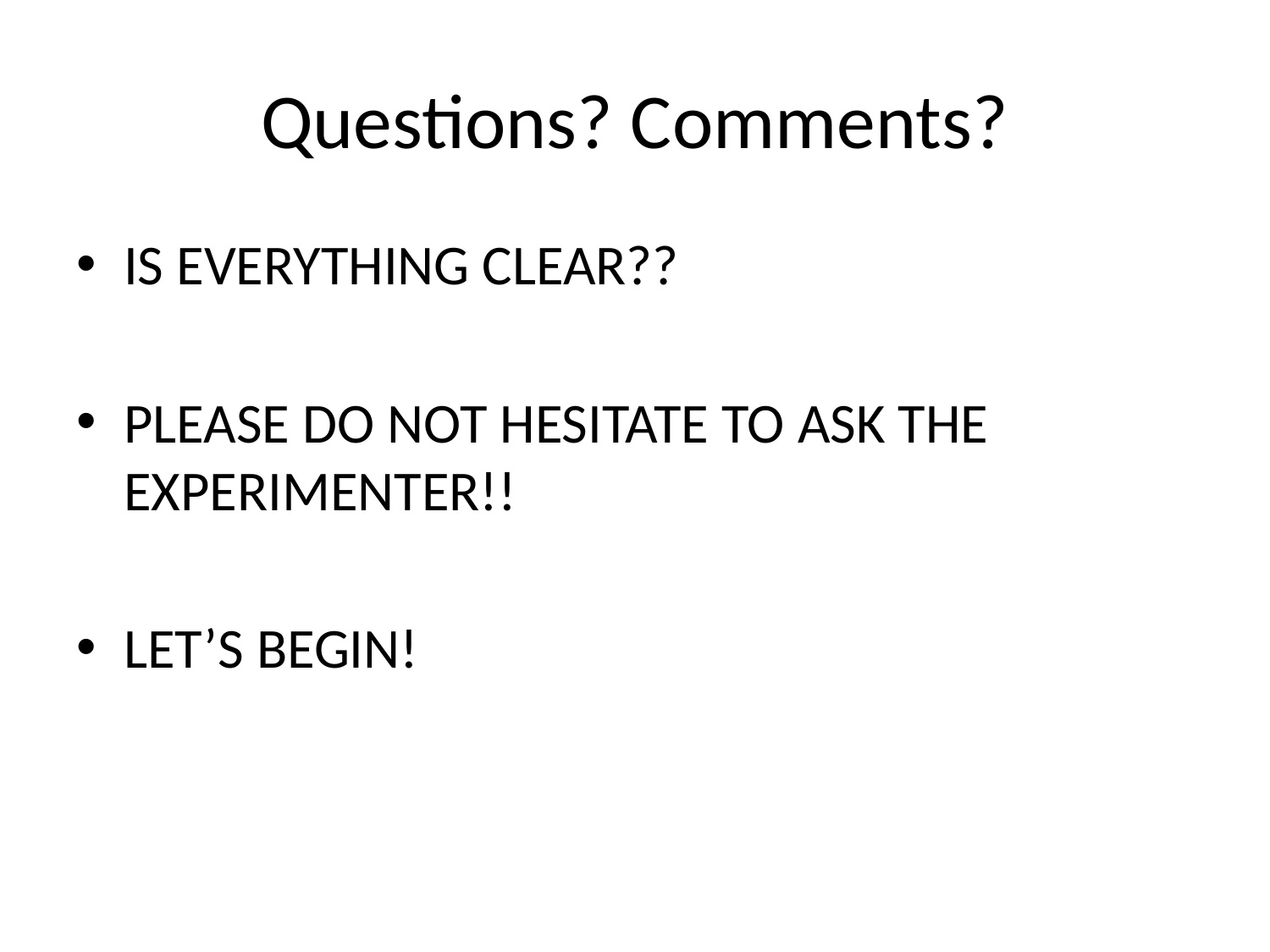

# Questions? Comments?
IS EVERYTHING CLEAR??
PLEASE DO NOT HESITATE TO ASK THE EXPERIMENTER!!
LET’S BEGIN!
