# Supplementary material for: Model based planners reflect on their model-free propensities
Source: PLoS Comput Biol. 2021 Jan 7;17(1):e1008552. doi: 10.1371/journal.pcbi.1008552 (PMC7817042; doi:10.1371/journal.pcbi.1008552)
Supplement: S2 Presentation — (PPTX) [file pcbi.1008552.s009.pptx]

## Slide 1
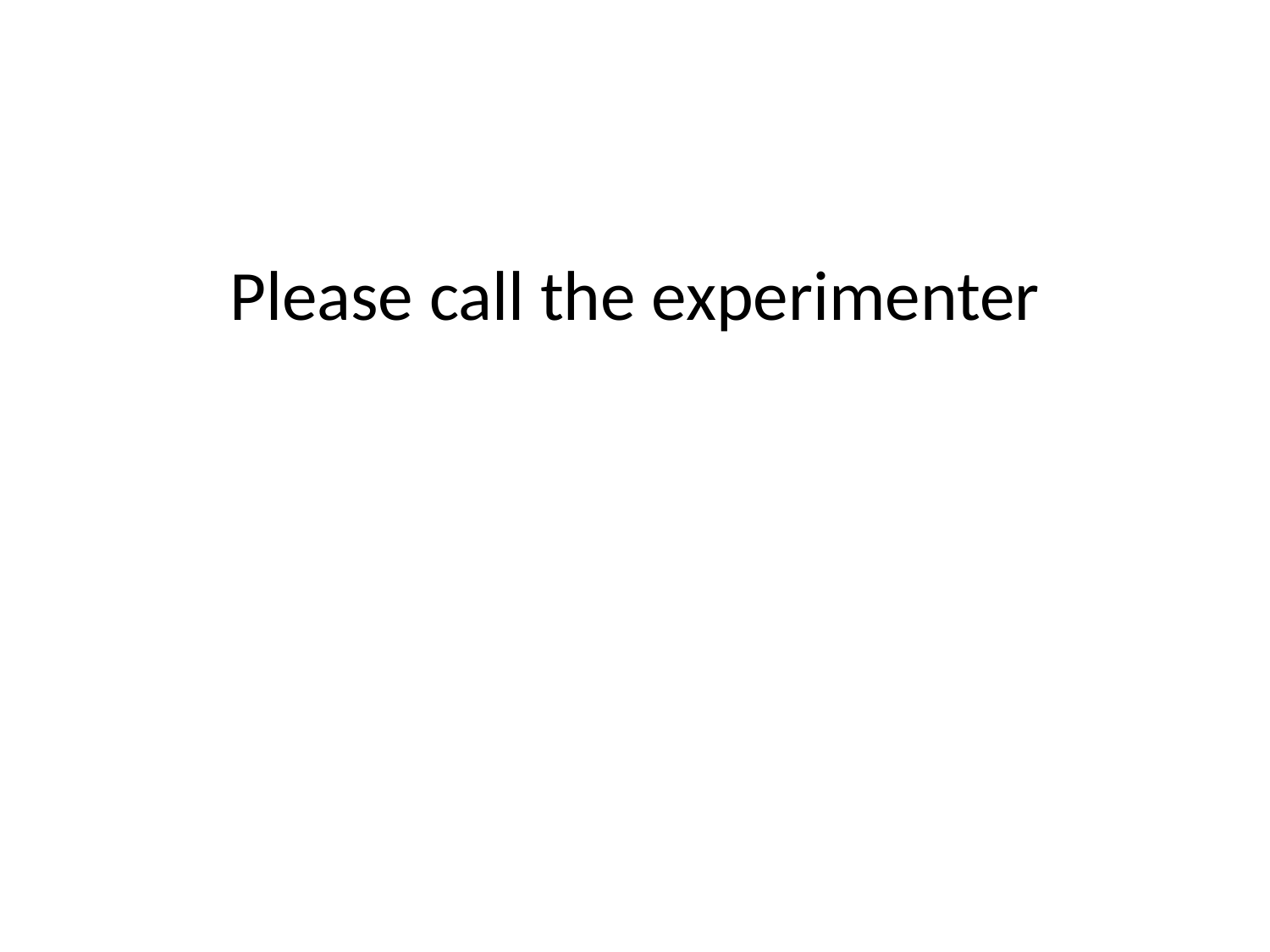

Please call the experimenter

## Slide 2
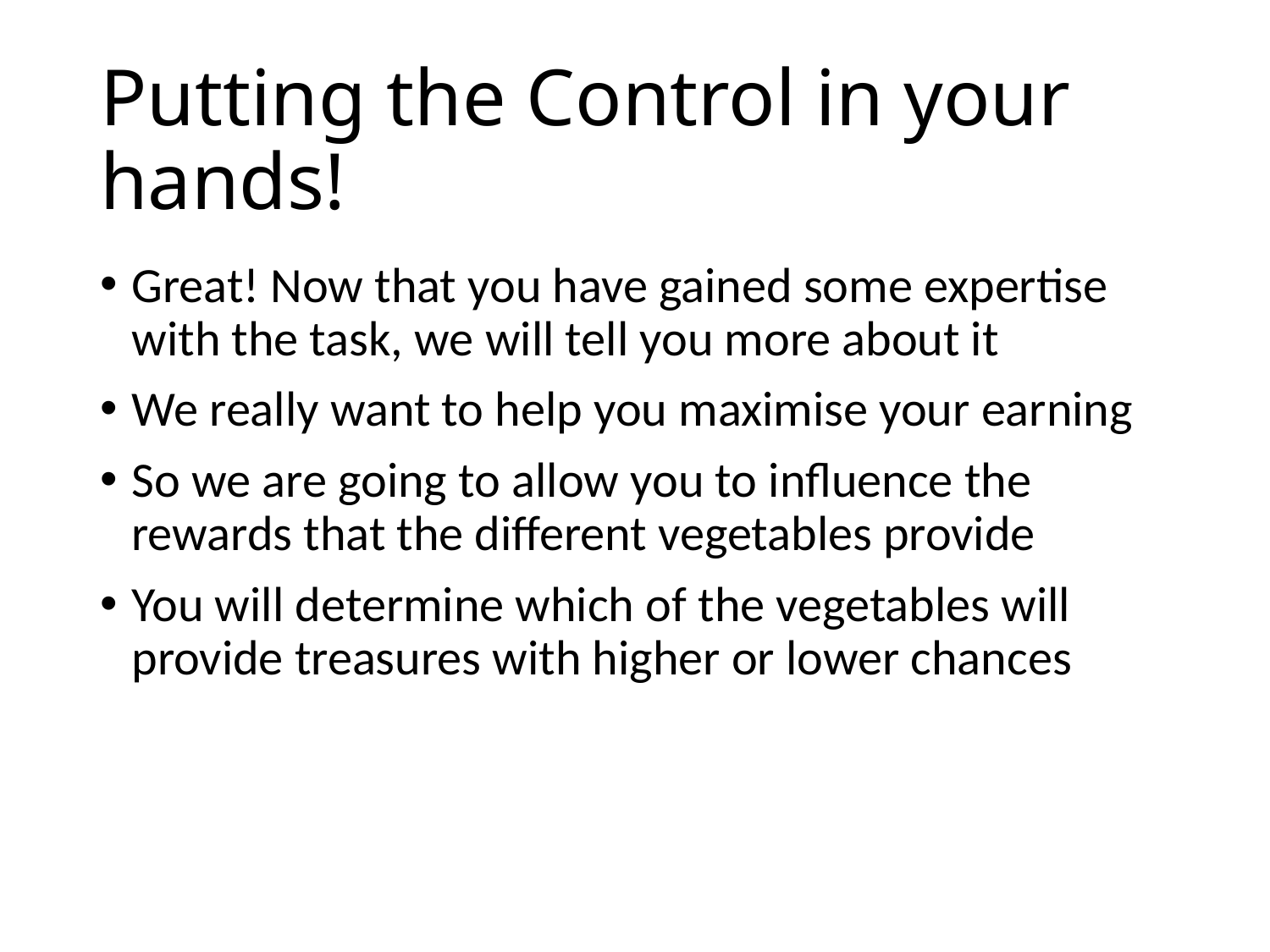

# Putting the Control in your hands!
Great! Now that you have gained some expertise with the task, we will tell you more about it
We really want to help you maximise your earning
So we are going to allow you to influence the rewards that the different vegetables provide
You will determine which of the vegetables will provide treasures with higher or lower chances

## Slide 3
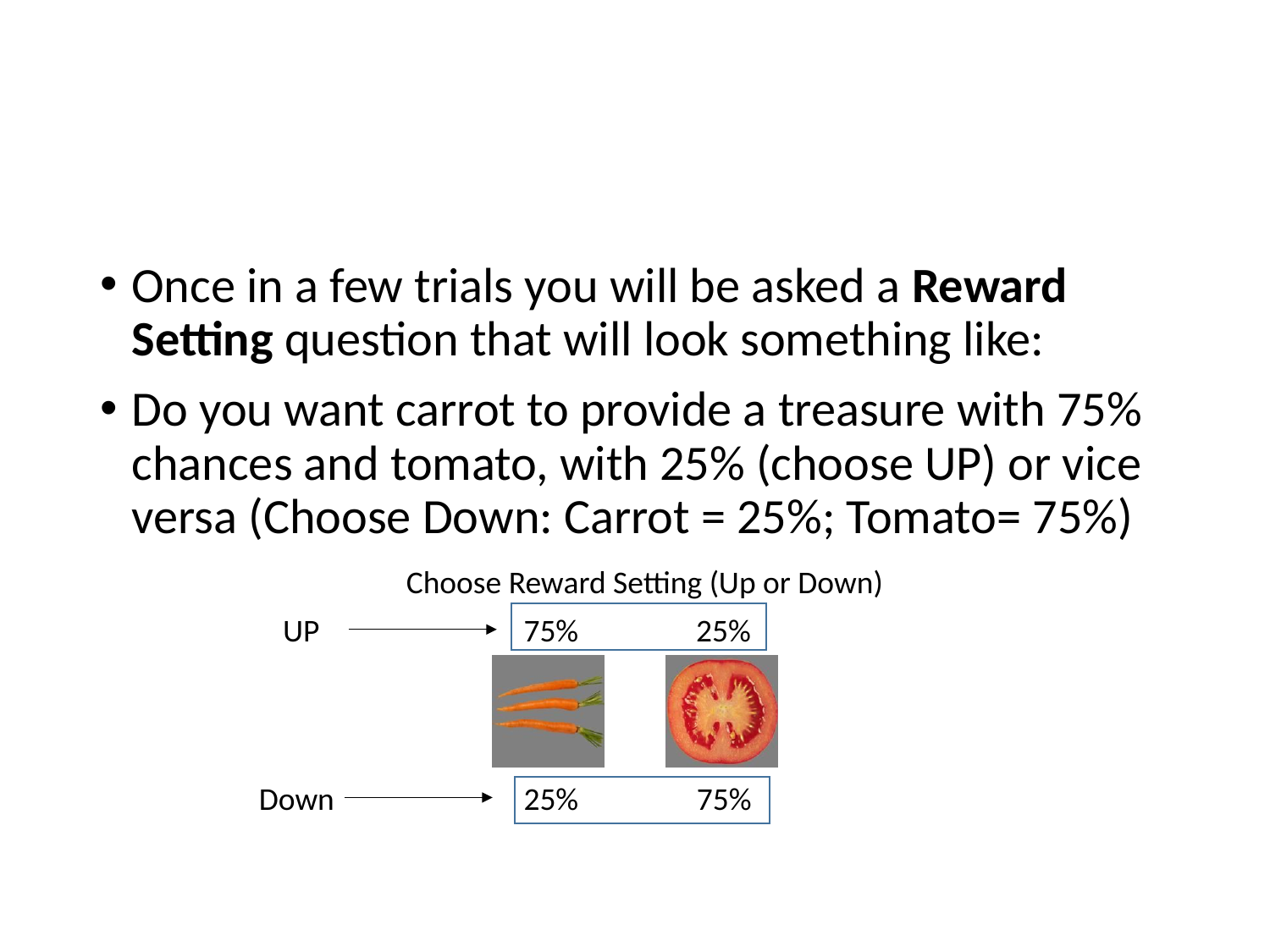

Once in a few trials you will be asked a Reward Setting question that will look something like:
Do you want carrot to provide a treasure with 75% chances and tomato, with 25% (choose UP) or vice versa (Choose Down: Carrot = 25%; Tomato= 75%)
Choose Reward Setting (Up or Down)
UP
75%
25%
Down
25%
75%

## Slide 4
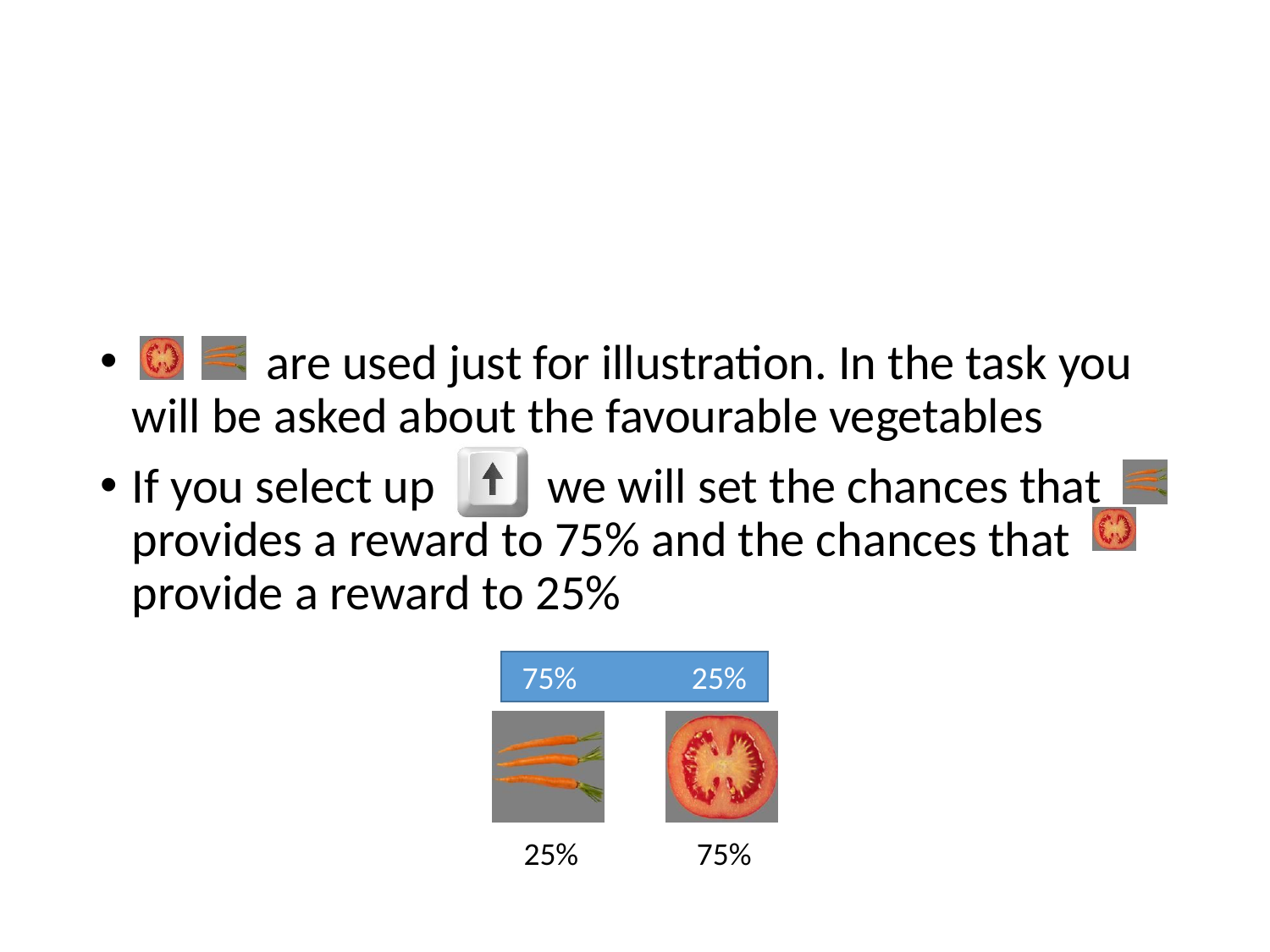

are used just for illustration. In the task you will be asked about the favourable vegetables
If you select up we will set the chances that provides a reward to 75% and the chances that provide a reward to 25%
75% 25%
25%
75%

## Slide 5
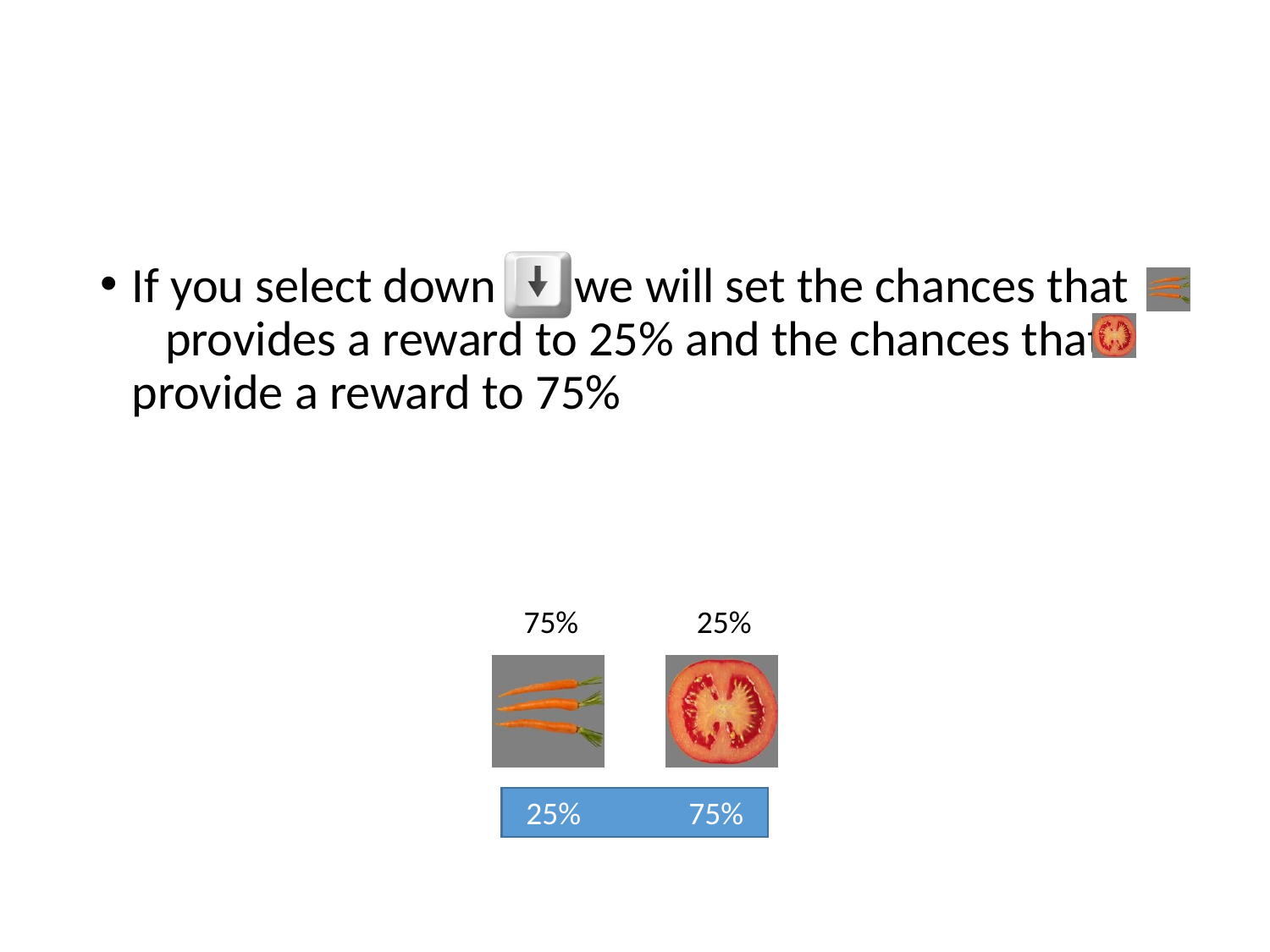

If you select down we will set the chances that provides a reward to 25% and the chances that provide a reward to 75%
75%
25%
25% 75%

## Slide 6
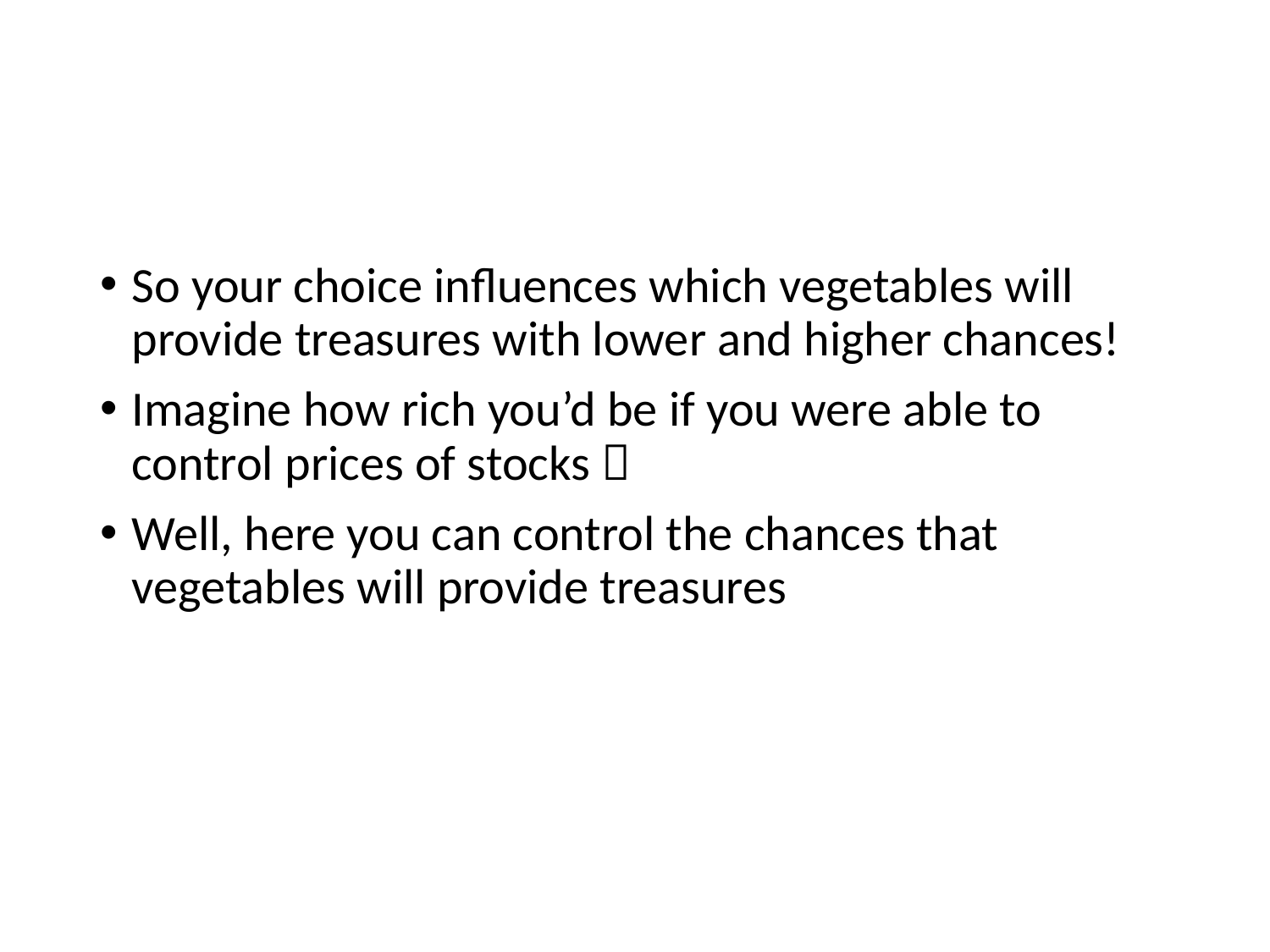

So your choice influences which vegetables will provide treasures with lower and higher chances!
Imagine how rich you’d be if you were able to control prices of stocks 
Well, here you can control the chances that vegetables will provide treasures

## Slide 7
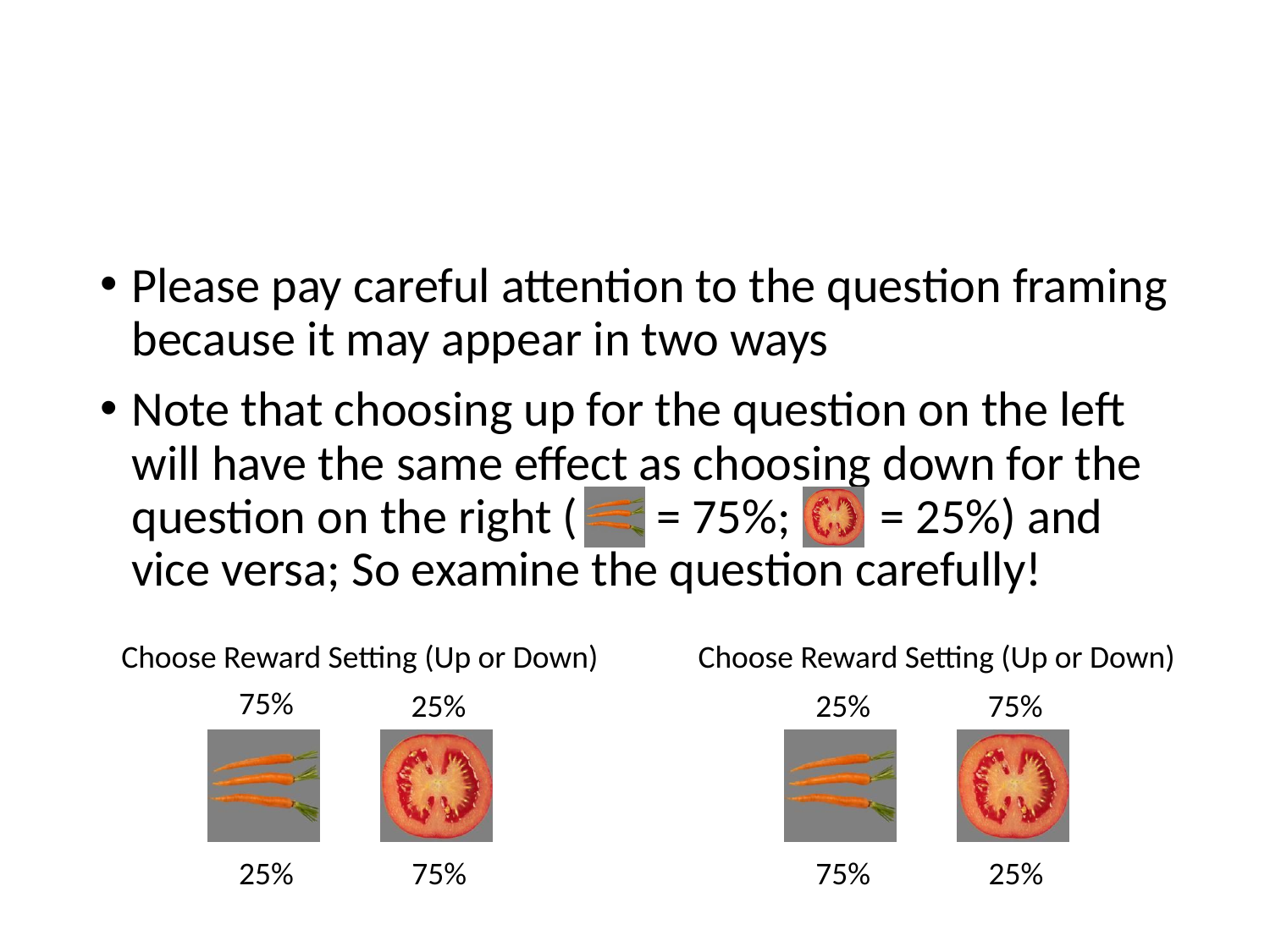

Please pay careful attention to the question framing because it may appear in two ways
Note that choosing up for the question on the left will have the same effect as choosing down for the question on the right ( = 75%; = 25%) and vice versa; So examine the question carefully!
Choose Reward Setting (Up or Down)
Choose Reward Setting (Up or Down)
75%
25%
25%
75%
25%
75%
75%
25%

## Slide 8
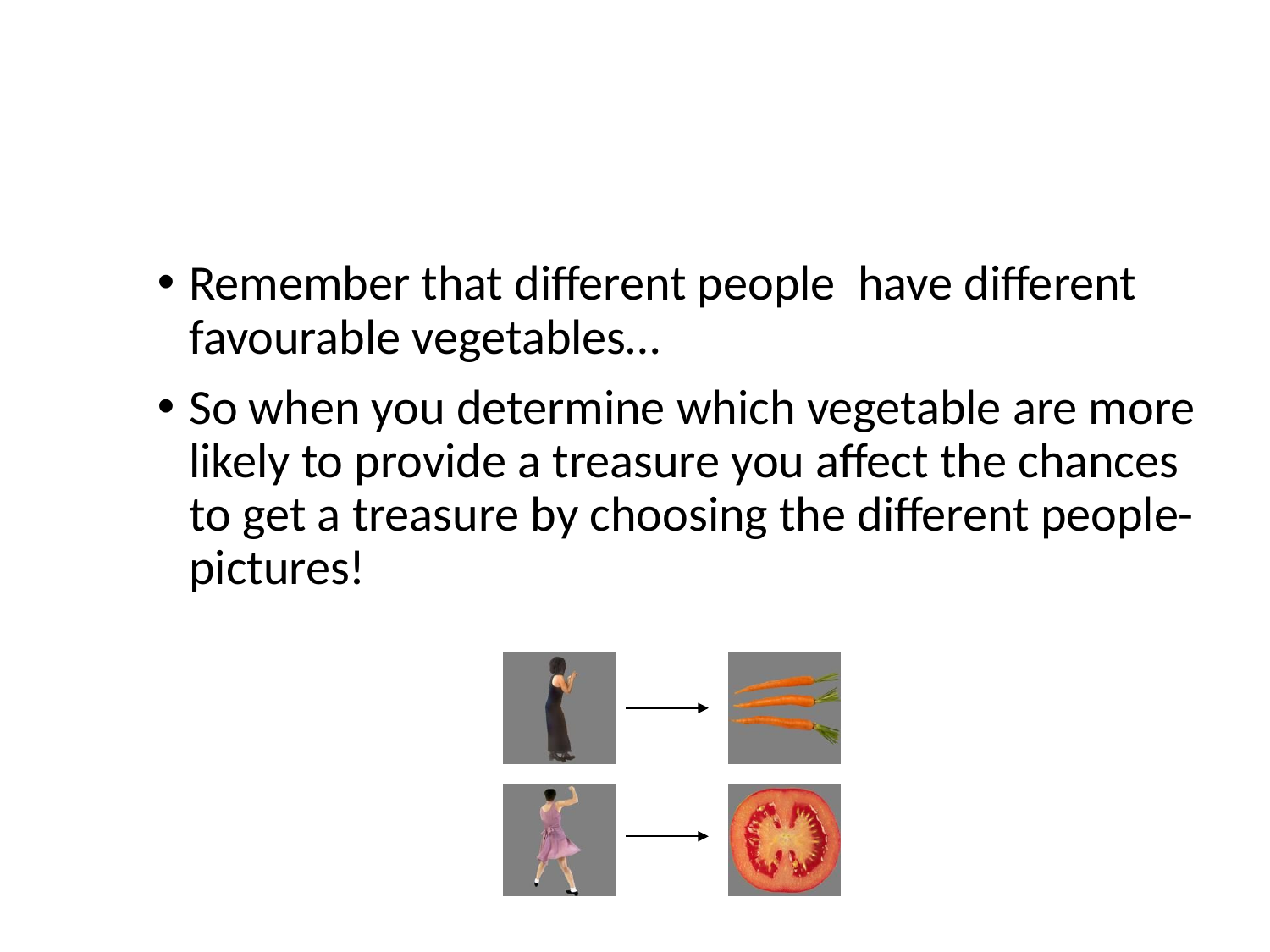

Remember that different people have different favourable vegetables…
So when you determine which vegetable are more likely to provide a treasure you affect the chances to get a treasure by choosing the different people-pictures!

## Slide 9
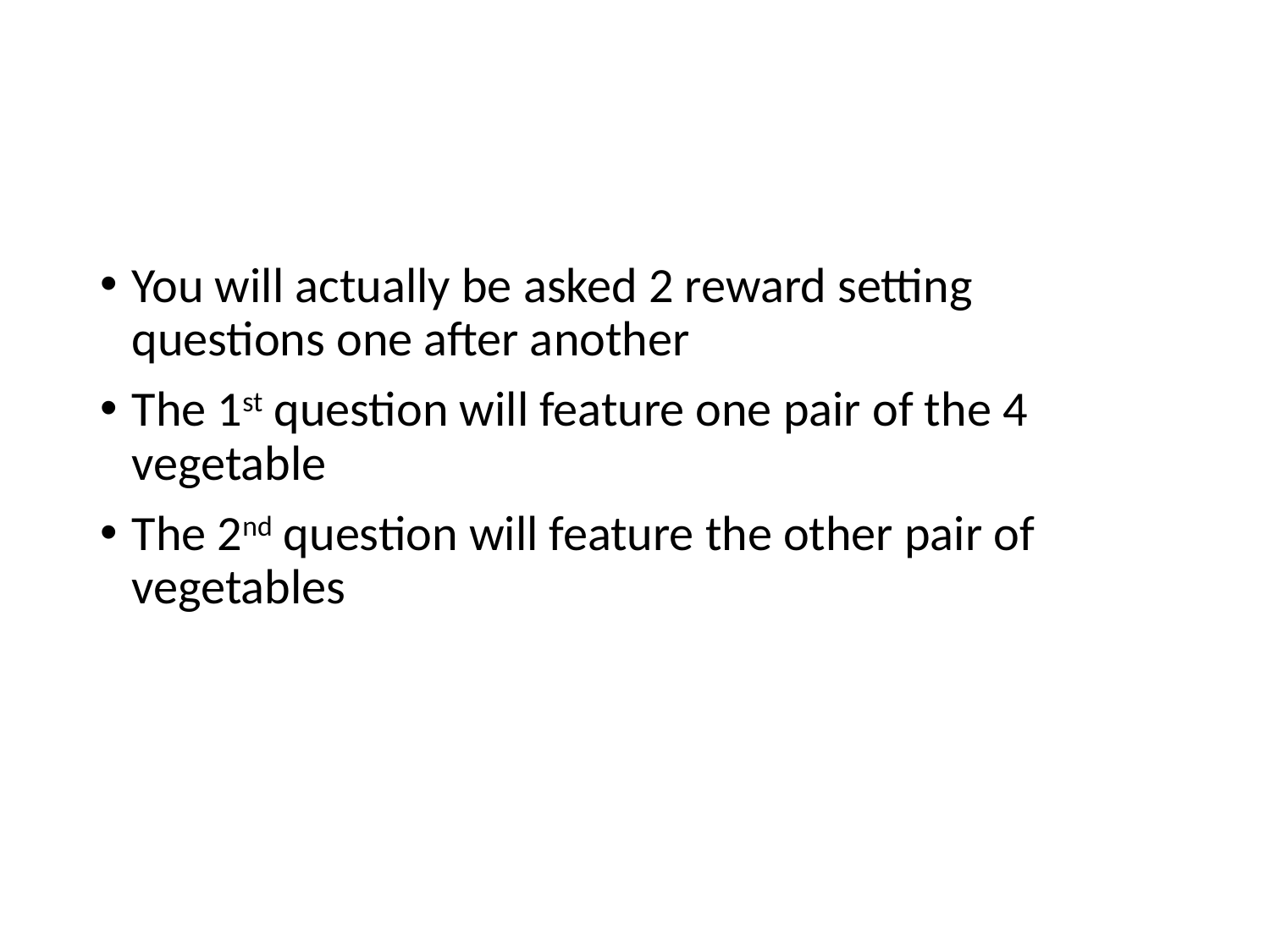

You will actually be asked 2 reward setting questions one after another
The 1st question will feature one pair of the 4 vegetable
The 2nd question will feature the other pair of vegetables

## Slide 10
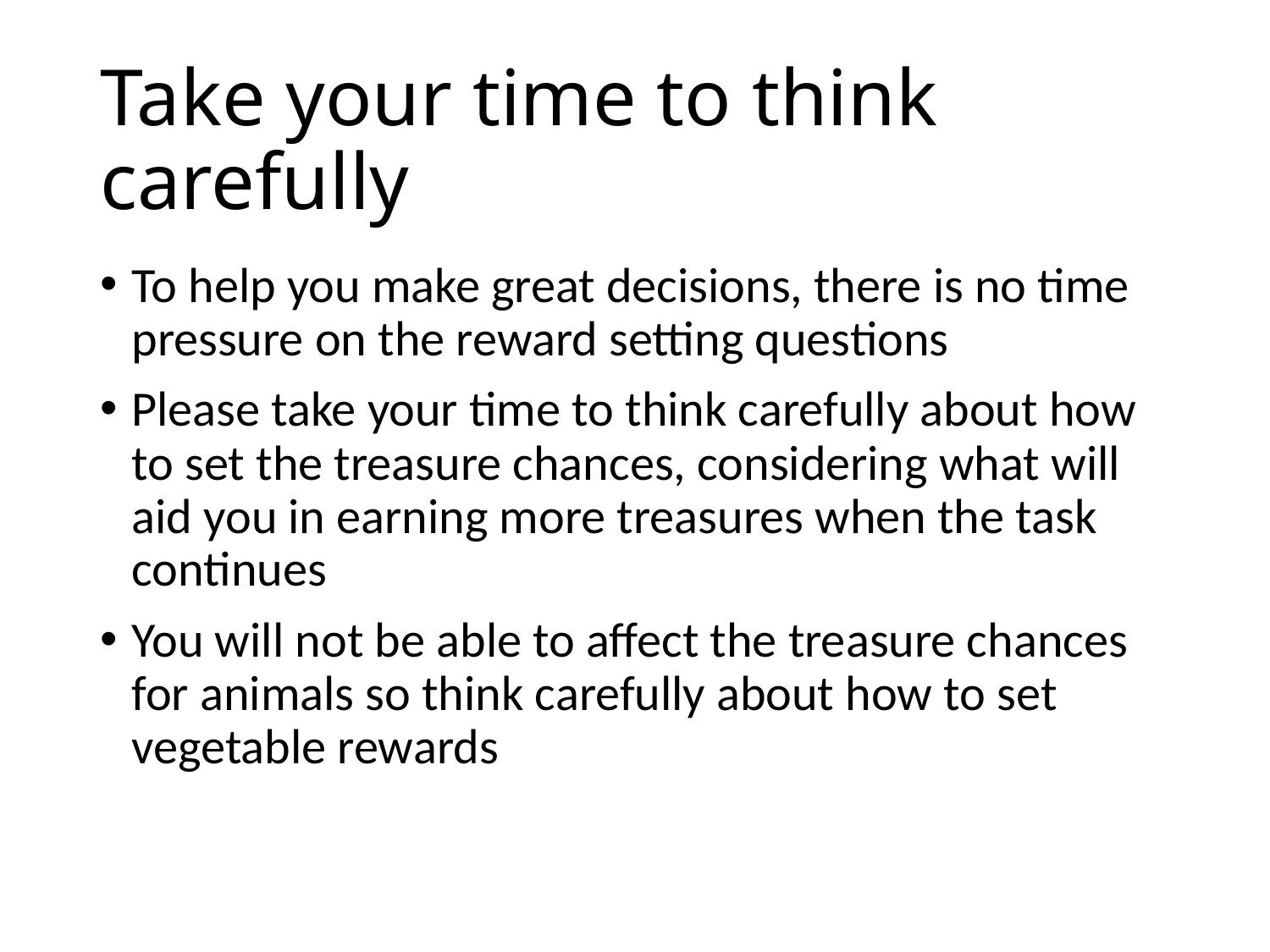

# Take your time to think carefully
To help you make great decisions, there is no time pressure on the reward setting questions
Please take your time to think carefully about how to set the treasure chances, considering what will aid you in earning more treasures when the task continues
You will not be able to affect the treasure chances for animals so think carefully about how to set vegetable rewards

## Slide 11
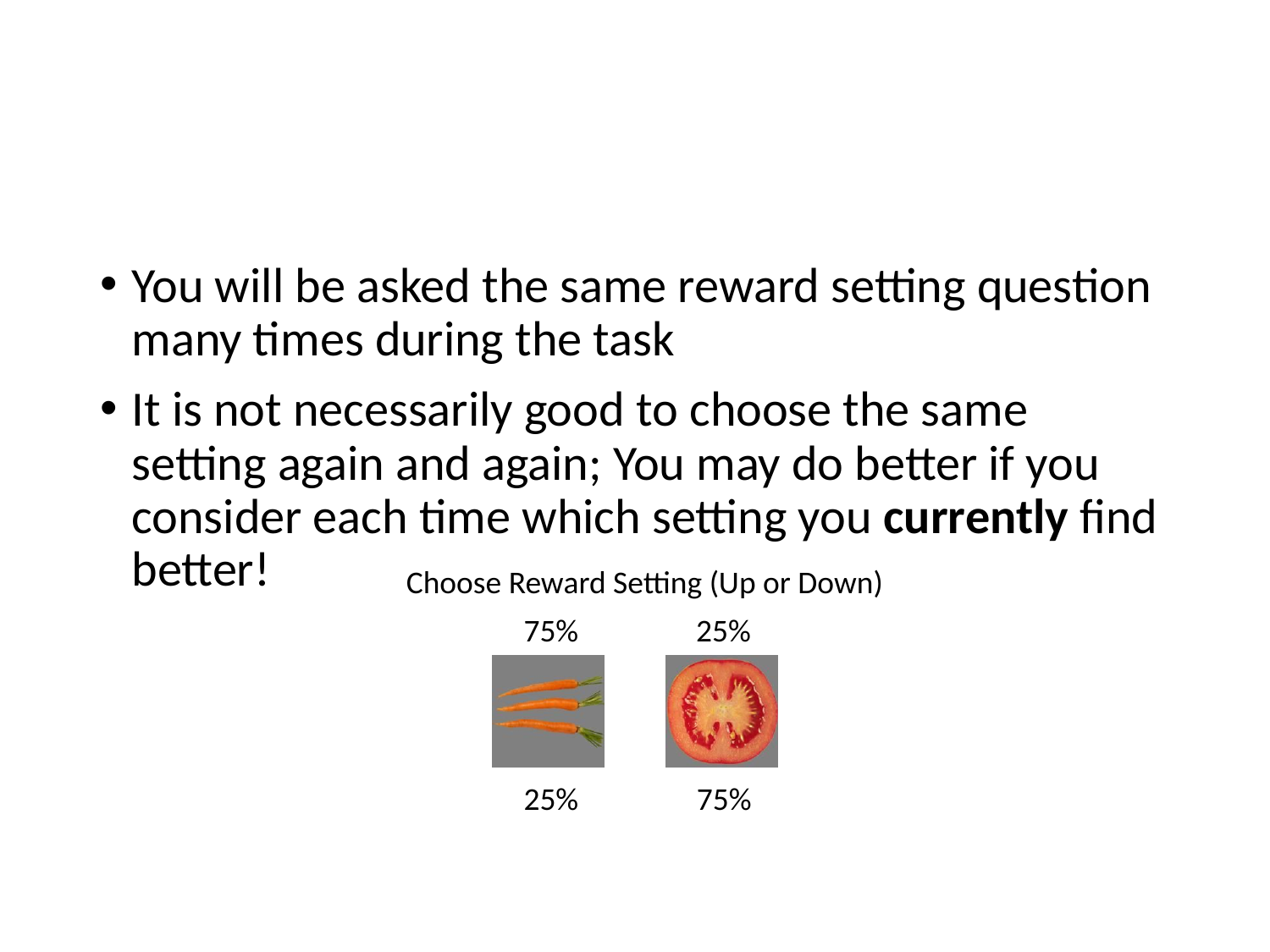

You will be asked the same reward setting question many times during the task
It is not necessarily good to choose the same setting again and again; You may do better if you consider each time which setting you currently find better!
Choose Reward Setting (Up or Down)
75%
25%
25%
75%

## Slide 12
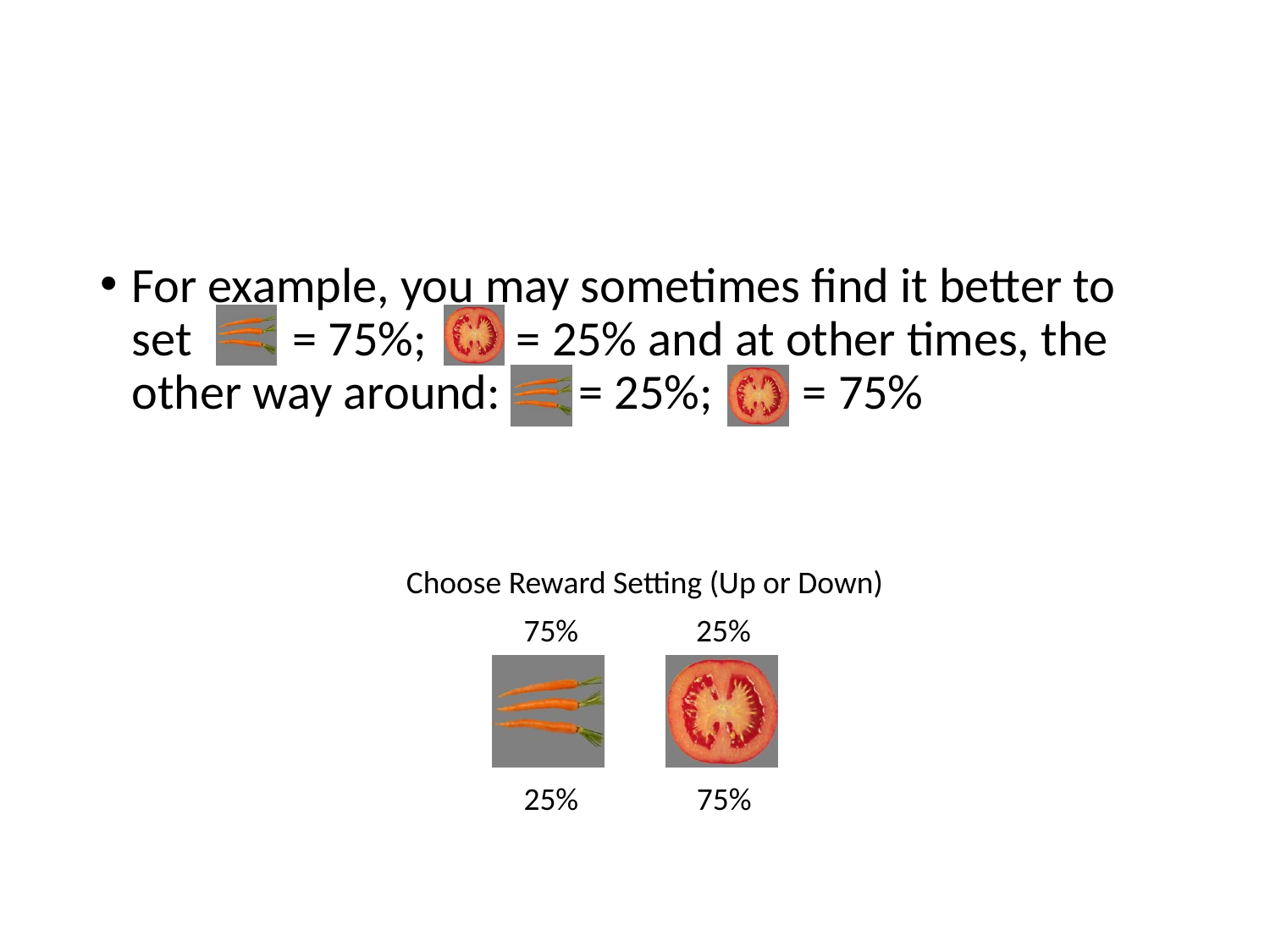

For example, you may sometimes find it better to set = 75%; = 25% and at other times, the other way around: = 25%; = 75%
Choose Reward Setting (Up or Down)
75%
25%
25%
75%

## Slide 13
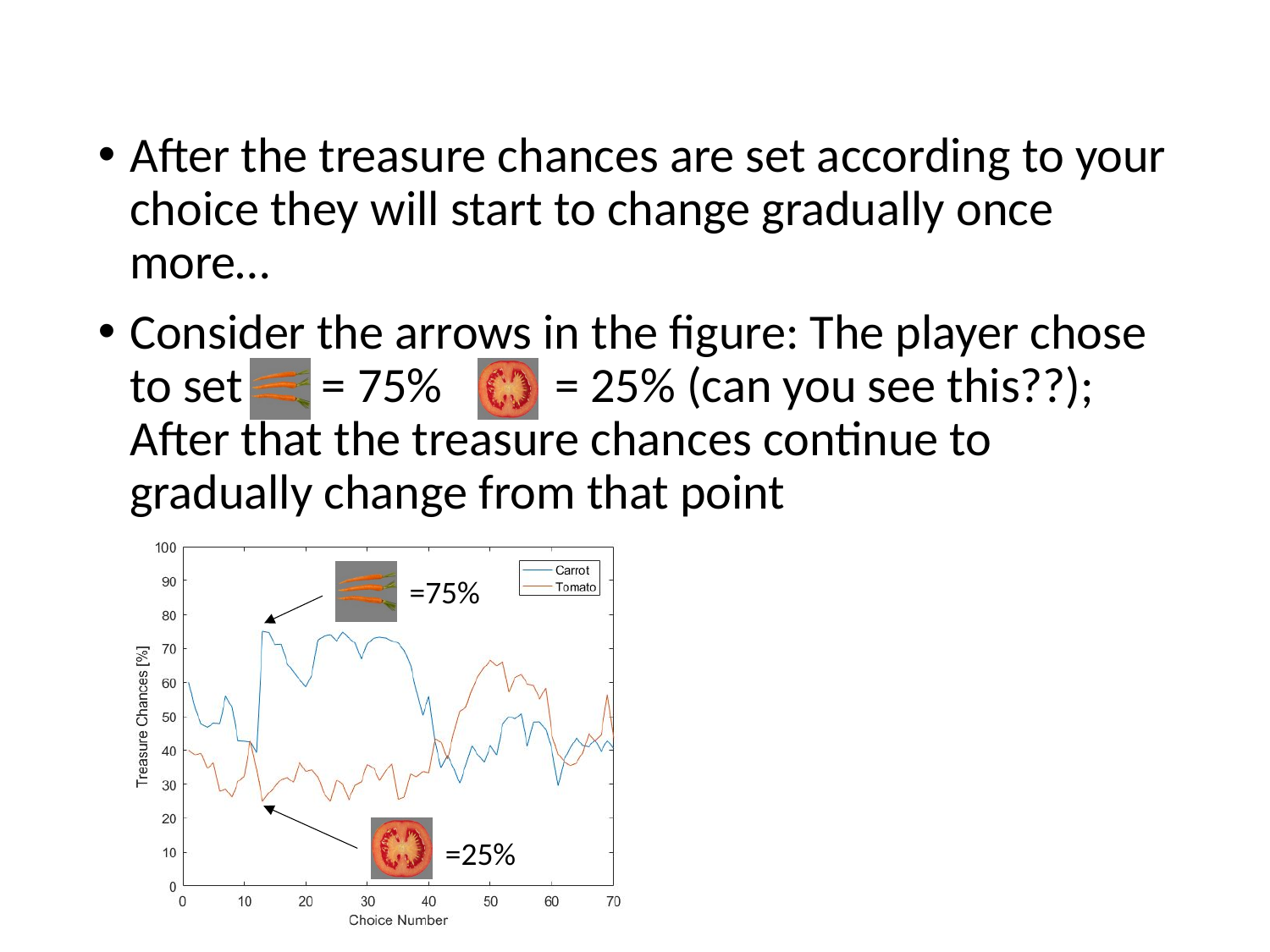

After the treasure chances are set according to your choice they will start to change gradually once more…
Consider the arrows in the figure: The player chose to set = 75% = 25% (can you see this??); After that the treasure chances continue to gradually change from that point
=75%
=25%

## Slide 14
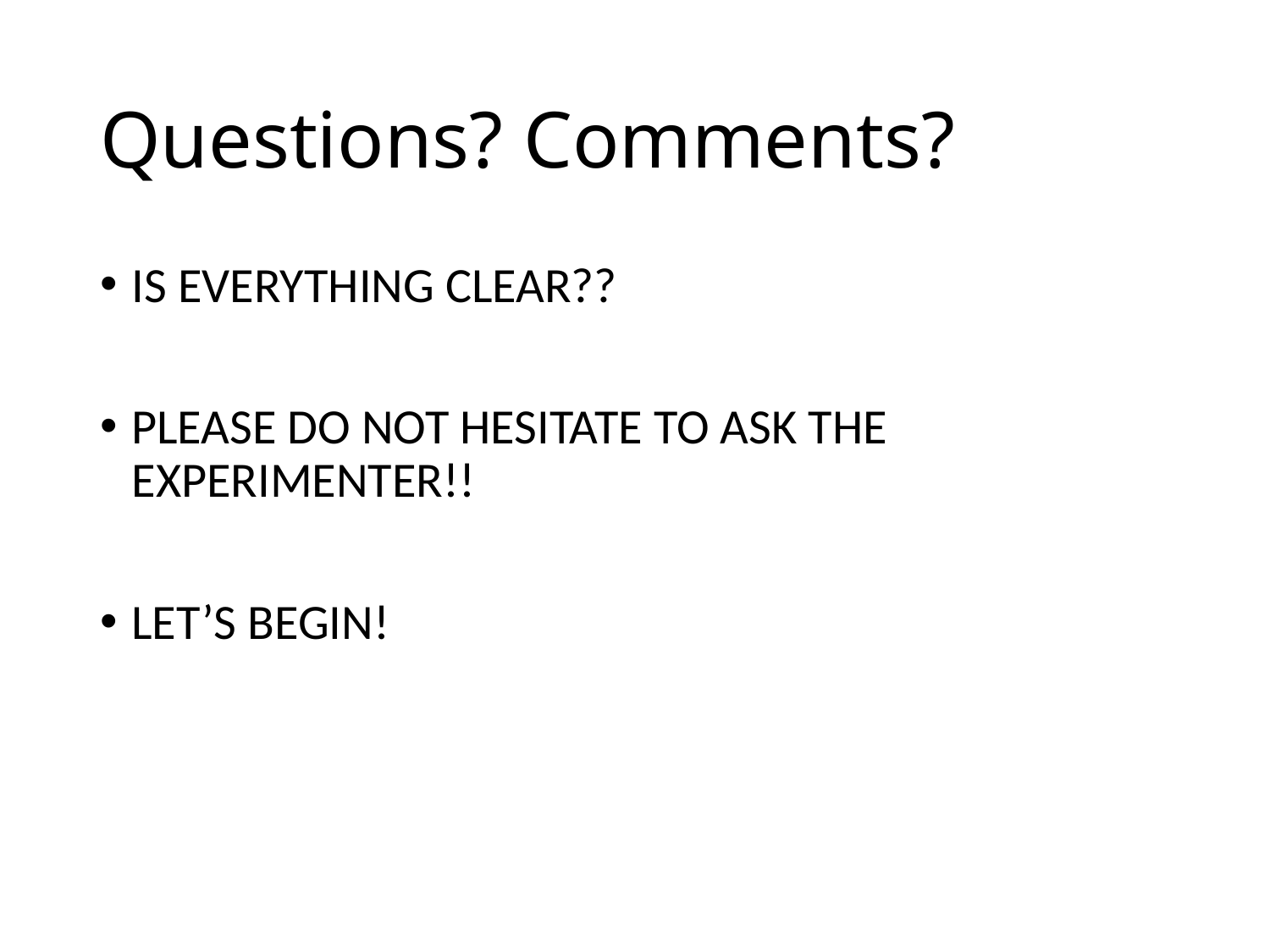

# Questions? Comments?
IS EVERYTHING CLEAR??
PLEASE DO NOT HESITATE TO ASK THE EXPERIMENTER!!
LET’S BEGIN!
